# Have compensatory mutations facilitated the current epidemic of multidrug-resistant tuberculosis?

**DOI:** 10.1038/s41426-018-0101-6

**Published:** 2018-06-06

**Authors:** Qingyun Liu, Tianyu Zuo, Peng Xu, Qi Jiang, Jie Wu, Mingyu Gan, Chongguang Yang, Ravi Prakash, Guofeng Zhu, Howard E. Takiff, Qian Gao

**Affiliations:** 1 Key Laboratory of Medical Molecular Virology at the School of Basic Medical Sciences, Shanghai Medical College, Shanghai Public Health Clinical Center, Fudan University, Shanghai, China; 2 Shenzhen Center for Chronic Disease Control, Shenzhen, China; 3 Key Laboratory of Characteristic Infectious Disease & Bio-safety Development of Guizhou Province Education Department, Zunyi Medical University, Zunyi, Guizhou China; 4 Shanghai Municipal Center for Disease Control and Prevention, Shanghai, China; 5 Department of Epidemiology of Microbial Diseases, School of Public Health, Yale University, New Haven, CT USA; 6 Unité de Génétique Mycobacterienne, Institut Pasteur, Paris, France

## Abstract

Compensatory mutations have been suggested to promote multidrug-resistant tuberculosis (MDR-TB) transmission, but their role in facilitating the recent transmission of MDR-TB is unclear. To investigate the epidemiological significance of compensatory mutations, we analyzed a four-year population-based collection of MDR-TB strains from Shanghai (the most populous city in China) and 1346 published global MDR-TB strains. We report that MDR-TB strains with compensatory mutations in the *rpoA*, *rpoB*, or *rpoC* genes were neither more frequently clustered nor found in larger clusters than those without compensatory mutations. Our results suggest that compensatory mutations are not a major contributor to the current epidemic of MDR-TB.

## Introduction

It had been thought that drug-resistance mutations would introduce a fitness cost into resistant *Mycobacterium tuberculosis* (*M. tb*)^1^, and the resulting reductions in virulence and transmissibility would prevent multidrug-resistant tuberculosis (MDR-TB) strains from disseminating widely^2,3^. Early mathematical models predicted that MDR-TB should remain a local problem^3^, but the steady growth of the MDR-TB epidemic worldwide has contradicted these early expectations. A subsequent study suggested that even if the average fitness of MDR-TB strains is low, a small proportion of resistant strains that are relatively more fit will outcompete the less fit and drug-susceptible strains^4^. These more fit, resistant strains were thought to contain compensatory mutations that would restore fitness and thus constitute an important factor in the spread of MDR-TB strains^5,6^, and MDR-TB outbreaks in HIV-negative patients were regarded as successful examples of compensatory evolution^7^.

Recent studies have investigated the role of compensatory mutations in MDR-TB transmission by comparing the presence of compensatory mutations in clustered or nonclustered strains^8,9^ and found that putative compensatory mutations in *rpoC* and *rpoA* were more common in VNTR-clustered strains. However, these studies did not determine whether the putative compensatory mutations accumulated before or after the MDR-TB strains were transmitted. If these compensatory mutations accumulated after transmission, they should not be considered as factors that promote or facilitate transmission. Moreover, the sampling methods used in these studies were not population based, and the IS6110/MIRU-VNTR defined clusters could have perhaps been further separated by whole-genome sequencing (WGS). To avoid these pitfalls, we examined the role of compensatory mutations in MDR-TB transmission by using WGS to determine transmission clusters in the MDR-TB strains collected in Shanghai over a 4-year period (2009–2012). Our findings contradict the previous inferences and do not support a significant role of compensatory mutations in promoting the ongoing MDR-TB epidemic.

## Results

### Collection of MDR-TB isolates

From 2009 to 2012, a total of 324 MDR-TB isolates were collected from 31 tuberculosis (TB) hospitals in Shanghai, China. All of these isolates were MIRU-VNTR genotyped, and 122 clustered MDR-TB strains had undergone WGS previously^10^. Here, to determine the presence of compensatory mutations in VNTR nonclustered MDR-TB strains, we randomly selected 105 isolates from the remaining 202 VNTR nonclustered MDR-TB isolates to sequence the full-length genes *rpoA*, *rpoB*, and *rpoC*. The combined results of the 227 Shanghai MDR-TB strains were termed the “Shanghai dataset”. Meanwhile, we obtained WGS records of 8331 *M. tb* isolates of global origin from the European Nucleotide Archive (ENA) (Supplementary Table 1), and 1346 of these strains were identified as MDR-TB (Supplementary Table 3). Of these MDR-TB strains, 602 were collected through retrospective cohorts or a population-based approach (Supplementary Table 1) and thus were appropriate for the subsequent analysis. The data from the global MDR-TB isolates were termed the “Global dataset”.

### Identification of putative compensatory mutations and transmission clusters

**Fig. 1:**
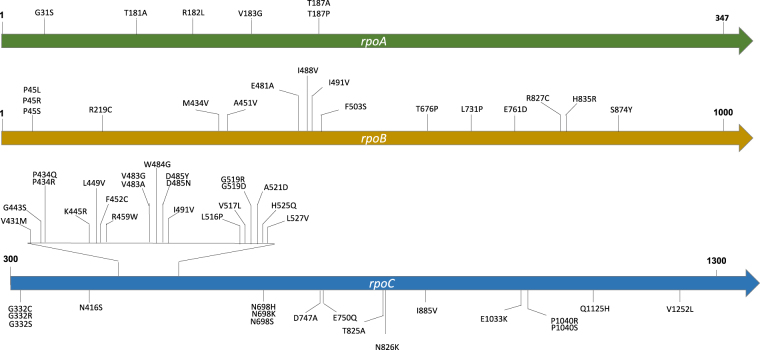
Putative compensatory mutations in the rpoA, rpoB, and rpoC genes identified in this study. Each putative compensatory mutation was supported by at least two independent evolution events

Through phylogenetic reconstruction of the MDR-TB strains obtained above, we found 60 nonsynonymous mutations in the *rpoA*, *rpoB*, or *rpoC* genes that had arisen at least twice in parallel and were identified as putative compensatory mutations (Fig. 1, Supplementary Table 4). Of these mutations, six were in *rpoA*, 16 in *rpoB*, and 38 in *rpoC*. We set “mutation parallelism” as a criterion to exclude lineage mutations or neutral polymorphisms that were fixed due to genetic drift. Although this filter might also exclude some uncommon compensatory mutations, it increased the level of confidence that the mutations identified were truly compensatory mutations. We included *rpoB* in the search for putative compensatory mutations because additional mutations in *rpoB* have been reported to both restore fitness and increase the level of rifampicin resistance^7^. MDR-TB strains with less than 12 Single-nucleotide polymorphisms (SNPs) difference were considered to constitute a transmission cluster. A total of 36 such clusters were found in the Shanghai dataset, and 117 clusters were identified in the Global dataset.

### Three types of transmission clusters

**Fig. 2:**
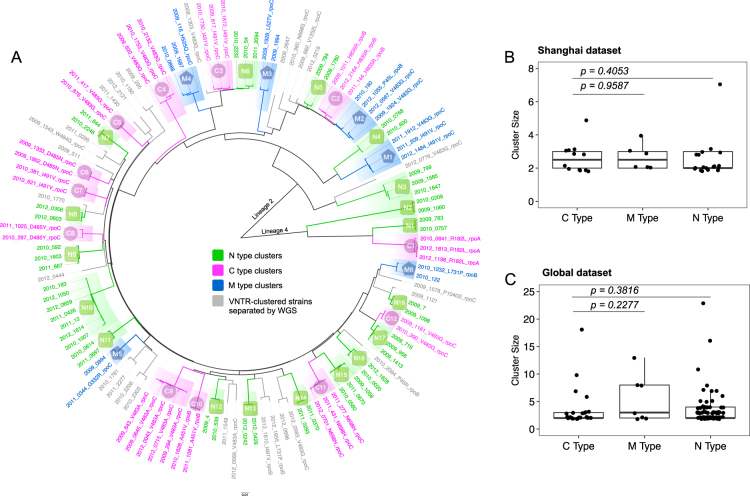
Compensated MDR-TB strains were not associated with larger transmission clusters. **a** A maximum likelihood phylogenetic tree showing genomic clusters in the Shanghai dataset. The strain identifiers were “year, strain number, and compensatory mutation type”. The three genomic cluster types are illustrated with different colors, as indicated. The isolate names in gray represent the VNTR-clustered strains that were separated by WGS. Comparison of cluster sizes in C-type, M-type, and N-type clusters in the Shanghai MDR-TB dataset (**b**) and the Global MDR-TB dataset (**c**); each dot represents a genomic cluster identified by WGS

To distinguish whether the compensatory mutations had occurred before or after transmission, we divided the MDR-TB clusters into three types (Fig. 2a): (1) C (compensated)-type clusters, in which all strains harbor the same compensatory mutation—indicating transmission of a compensated MDR-TB strain (compensatory mutations occurring before transmission); (2) N (noncompensated)-type clusters, containing only strains with no putative compensatory mutation—indicating transmission of a noncompensated MDR-TB strain; (3) M (mixed)-type clusters, in which MDR-TB strains harbored different compensatory mutations or only a proportion of the strains in the cluster contained compensatory mutations, indicating that the compensatory mutations had occurred after transmission (see more details about M-type clusters in the discussion). The groups so defined are theoretically similar to the three prototypical types of clusters used to evaluate the role of drug resistance in transmission^11^. In the Shanghai dataset, 12 transmission clusters were determined as C-type, 18 as N-type, and six as M-type (Fig. 2a); in the Global dataset, 26 transmission clusters were determined as C-type, 84 as N-type, and seven as M-type.

### Compensated MDR-TB strains did not cause larger transmission clusters

If the putative compensatory mutations stimulated the transmission of MDR-TB, then C-type clusters would be expected to be larger than N-type clusters, but in the Shanghai strains, the C-type clusters were not larger than either the N-type or the M-type clusters (Wilcoxon rank-sum test, *P* = 0.4053 and 0.9587, respectively) (Fig. 2b). An analysis of the size of the clusters in the global MDR-TB strains yielded similar results (Wilcoxon rank-sum test, *P* = 0.3816 and 0.2277, respectively) (Fig. 2c). Notably, the largest clusters observed in both the Shanghai and Global datasets (with seven and 23 linked cases, respectively) were caused by MDR-TB strains without compensatory mutations (Fig. 2b, c). Moreover, we did not find any particular compensatory mutation that was associated with larger transmission clusters in either dataset. These results suggest that compensated MDR-TB strains are not prone to generate more secondary cases than noncompensated MDR-TB strains do.

### Compensated MDR-TB strains were not more likely to be clustered

Previous studies suggested that MDR-TB strains with compensatory mutations were more frequently clustered^8,9^. However, these studies treated “compensatory mutations” as a static feature and ignored the possibility that compensatory mutations could occur in secondary cases that were initially infected with noncompensated MDR-TB strains. This is probably what has occurred in the M-type clusters (Fig. 2a), and therefore, it should not be considered as representing the transmission of compensated MDR-TB strains. Accordingly, we excluded M-type clusters from an analysis of the ratios of compensatory mutations in the clustered and nonclustered groups, and compared only C-type and N-type clusters. Surprisingly, no significant difference was observed in the ratios of compensatory mutations in clustered compared to nonclustered groups, indicating that compensated MDR-TB strains were not more frequently clustered (Table 1).

**Table 1:** Ratios of compensated strains in clustered and nonclustered MDR-TB groups

Groups	Total	With CMs^a^	Without CMs^a^	*χ* ^2^	*P* value
Clustered MDR-TB (%)^b^	78	32 (41.0%)	46 (59.0%)	2.260	0.133
Nonclustered MDR-TB (%)	133	41 (30.8%)	92 (69.2%)		

^a^CMs compensatory mutations

^b^Excluded M-type clusters

To allow a comparison with previous studies, we repeated the analysis without excluding the M-type clusters. When the M-type clusters were included, the ratio of compensatory mutations in the clustered group was significantly higher than that in the nonclustered group (Supplementary Table 5), similar to the results of previous studies. In our M clusters, 62.5% (10/16) of MDR-TB strains harbored compensatory mutations, which was much higher than that of either clustered (41.0%) or nonclustered (30.8%) groups. M-type clusters are those in which compensatory mutations are present in only some of the strains in a cluster, presumably because the mutations have occurred during the transmission of the clustered strain. Therefore, when the M-type clusters are included with the clustered MDR-TB strains, the mutations that occurred after transmission result in a higher ratio of compensatory mutations in clustered than in nonclustered MDR-TB strains.

### The dilution effect of recently formed MDR-TB strains

**Fig. 3:**
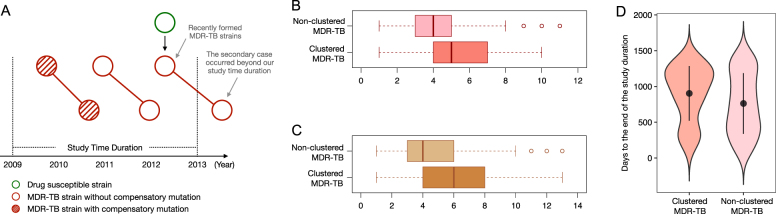
Recently formed MDR-TB strains would decrease the ratio of compensatory mutations in the nonclustered MDR-TB group. **a** A theoretical scheme shows that recently formed MDR-TB strains would be included, while the secondary clustered strains resulting from transmission would occur outside of the study’s observation period. Thus, newly formed MDR-TB strains would be assigned to the nonclustered group. This schematic diagram also shows that transmission that occurred within the first 2 years will be captured in our study, while the secondary cases resulting from transmission that occurred during the last 2 years could be beyond the study’s observation period. **b** Comparison of the number of drugs to which strains are resistant in clustered and nonclustered MDR-TB strains. **c** Comparison of the number of drug-resistance mutations in clustered and nonclustered MDR-TB strains. **d** Comparison of collection time distribution between clustered and nonclustered MDR-TB groups; the collection time of each isolate was counted as “days to the end of the study duration (31 December 2012)”

Even when the M-type clusters were excluded, the clustered group had a nonsignificantly higher ratio of compensatory mutations than that of the nonclustered group (41.0% versus 30.8%). One explanation might be that new MDR-TB strains are continuously emerging during antibiotic treatment, and these new MDR-TB strains might not have had enough time to accumulate compensatory mutations. For the same reason, within the 4-year duration of the study, they might not have had enough time to cause secondary TB cases (Fig. 3a). Thus, these recently formed “young” MDR-TB strains would be assigned to the nonclustered group and thereby decrease the ratio of compensatory mutations in this group. In contrast, all strains in MDR-TB clusters are relatively “older” (had become MDR-TB prior to their transmission), and thus would have had a longer period to accumulate additional drug resistance and compensatory mutations than strains in the nonclustered group. If this is true, then clustered MDR-TB strains should have wider drug-resistance spectra (number of drugs to which they are resistant) than those of nonclustered MDR-TB strains.

To test this inference, we compared the drug-resistance spectra of the clustered versus nonclustered MDR-TB strains in the Global dataset. This analysis demonstrated that the median number of antibiotics to which nonclustered MDR-TB strains were resistant was 4.22 (4.10 ~ 4.34), while for clustered MDR-TB strains it was 5.13 (5.05 ~ 5.21) (Student’s *t*-test, *P* < 0.0001, Fig. 3b). In addition, as a single *M. tb* strain can accumulate multiple mutations that confer resistance to a single antibiotic (evolution of high-level resistance)^12–14^, we further compared the numbers of drug-resistance-conferring mutations in these two groups. Consistent with our prediction, the clustered MDR-TB strains had, on average, more drug-resistance mutations than the nonclustered MDR-TB strains did (Student’s *t*-test, *P* < 0.0001, Fig. 3c).

Finally, to verify whether nonclustered MDR-TB strains tended to occur toward the end of the study period, i.e., were indeed younger, we compared the distributions of the collection times of the initial cultured clinical specimens (counted as days to the end of the study duration) for the isolates in the clustered versus nonclustered groups. We found that the isolates in the nonclustered group had a shorter average time since collection than the isolates in the clustered group did (mean values: 762.08 versus 903.14, *P* < 0.0001, Wilcoxon rank-sum test, Fig. 3d), which suggests that the strains in the nonclustered group tended to be isolated toward the end of the study period. These analyses support our inference that clustered MDR-TB strains differ from nonclustered MDR-TB strains, suggesting a dilution effect from recently formed MDR-TB strains that could in turn provide an explanation for the relatively lower ratio of compensatory mutations in the nonclustered MDR-TB group.

## Discussion

Our findings suggest that, in contrast to published reports^5,15^, MDR-TB strains with compensatory mutations are not more frequently found in clusters, nor are they more likely to belong to larger transmission clusters than the MDR-TB strains without these mutations do. Previous studies have made direct comparisons of the number of strains with putative compensatory mutations in clustered versus nonclustered MDR-TB strains and reported an enrichment of compensatory mutations in the clustered group^8,9^. However, we observed that M-type clusters had an impact on this comparison because inclusion or exclusion of these clusters would dramatically alter the results. Hence, we infer that the previous observations would probably change if they excluded M-type clusters.

We believe in the accuracy of the results of our study because it incorporated several methodological improvements. First, we used WGS to identify transmission clusters, which is more precise than IS*6110* or MIRU-VNTR^16^. In our data, a total of 28 VNTR-clustered strains (23% of the VNTR-clustered strains) were further separated by WGS. Second, we set strict criteria to identify putative compensatory mutations and excluded phylogenetic/lineage mutations or neutral polymorphisms. Third, our analysis discriminated between the accumulation of compensatory mutations before or after the transmission of MDR-TB strains (i.e., C- versus M-type clusters) in the analysis of cluster size and the ratio of compensatory mutations.

The effect of drug resistance on transmission has been long debated^4,17^, and different studies have reported heterogeneous results suggesting that MDR-TB strains can be ten times more or ten times less transmissible than drug-susceptible strains^11,18^. However, the reasons underlying these variations could simply reflect the differences in study settings and methodologies employed^4,18^; the drug-resistant strains reported to have reduced transmissibility were found in settings with effective TB control^11^, while strains with increased transmissibility were reported from regions with high TB burdens^4,18^. Thus, it seems more likely that the heterogeneous and discordant published results serve to demonstrate that TB transmission is a process that is primarily influenced by environmental factors such as TB control policy, time with the illness before diagnosis, treatment efficacy, and general quality of the health-care system^19^.

We consider that there are at least four factors that can obscure the function of compensatory mutations in MDR-TB transmission. First, the efficacy of the TB control program. In countries or regions with high MDR-TB prevalence and poor management of MDR-TB patients, the transmission of MDR-TB strains is highly likely, even in the absence of compensatory mutations. In such circumstances, outbreaks and epidemics of MDR-TB are mainly driven by environmental factors^20,21^. Second, MDR-TB patients are, on average, infectious for longer periods due to the long course of treatment and high treatment failure rates^22,23^. Thus, there might be more chances for MDR-TB patients to generate secondary cases, which could conceivably compensate for a modest decrease in fitness. Third, the most common drug-resistance mutations carried by clinical isolates are associated with the lowest fitness cost in vitro, and such slight decreases in fitness may not be sufficient to affect transmission^1,6^. Moreover, positive epistatic effects between different drug-resistance mutations could further ameliorate the fitness cost imposed by individual drug-resistance mutations^17,24^. Fourth, it is still possible that the fitness cost measured in vitro might not reduce the ability of *M. tb* to transmit and establish in a new host in vivo^25,26^. This idea was supported by a previous study using tuberculin skin testing to trace infection that found an equal prevalence of infection among contacts exposed to patients harboring both drug-resistant and drug-susceptible strains^27^.

The objective of this study was to investigate the effects of compensatory mutations on MDR-TB transmissions that occurred within three or fewer years (recent transmission)^28^, and the 4-year observation window should have captured most of the transmissions that occurred within the first 2 years of the study. For transmissions that occurred during the last 2 years of the study, the secondary cases might have developed TB disease after our observation window. (Fig. 3a). However, we believe that the transmission pattern in the last 2 years should not substantially differ from that in the first 2 years, and therefore, further extending the time frame of our study would not change its major conclusion.

In conclusion, we did not observe a promoting influence of compensatory mutations on the transmission of MDR-TB, and we suggest that the putative contribution of compensatory mutations may be overwhelmed by the complex and more powerful effects of environmental factors.

## Methods

### MDR-TB datasets

A total of 324 MDR-TB isolates were collected from 1 January 2009 to 31 December 2012, in Shanghai (the most populous city in China) during a population-based observational study^10^. MIRU-VNTR genotyping (12 loci) was performed for all MDR-TB strains, and the clustered strains were further subjected to WGS^10^. In the present study, we further sequenced the entire gene sequences of the *rpoA*, *rpoB*, and *rpoC* genes in 105 nonclustered MDR-TB strains to detect putative compensatory mutations. The PCR amplicons were sent for Sanger sequencing, and the results were analyzed to detect putative compensatory mutations using *Geneious* (http://www.geneious.com/).

A total of 8331 published whole-genome sequences of *M. tb* strains were downloaded from the ENA (http://www.ebi.ac.uk/ena). The quality criteria for data inclusion were set as follows: (1) the average sequencing depth should be above 10-fold; (2) the genome coverage rate should be above 95%. To avoid false transmission clustering, we included only the last isolate of longitudinal isolates collected from the same patient. Information about the geographic isolation of these isolates was obtained from the articles or the authors.

### Analysis of WGS data

We used a validated pipeline for the mapping of sequencing reads to the reference genome^10,29^. In brief, the *Sickle*^30^ tool was used for trimming WGS data. Sequencing reads with Phred base quality above 20 and reads length longer than 30 were retained for analysis. The whole-genome sequence of the *M. tb* H37Rv strain (NC_000962.2) was used as the reference template for mapping reads. Sequencing reads were mapped to the reference genome using *Bowtie2* (v2.2.9)^31^. *SAMtools* (v1.3.1)^32^ was used for SNP calling with mapping quality greater than 30. Fixed mutations (frequency ≥75%) were identified using *VarScan* (v2.3.9)^33^ with at least ten reads supporting and strand bias filter option on. We excluded all SNPs located in noise or repetitive regions of the genome (e.g., PPE/PE-PGRS family genes, phage sequences, insertions or mobile genetic elements).

### Identification of putative compensatory mutations

The maximum likelihood method was used to reconstruct the phylogenetic trees with 500 bootstrap repeats using *MEGA* (v6.06)^34^, and the phylogenetic tree was further visualized by FigTree (http://tree.bio.ed.ac.uk/software/figtree/). Putative compensatory mutations in the *rpoA, rpoB*, or *rpoC* genes were identified through the following criteria: (1) nonsynonymous mutations in the *rpoA, rpoB* or *rpoC* genes that were present in only rifampicin-resistant strains; (2) each putative compensatory mutation must have arisen at least twice independently (parallel selection). We wrote a Python script to implement these criteria with the phylogenetic tree and a mutation matrix, and the Python script was uploaded to GitHub.

### Genotypic drug resistance detection and identification of transmission clusters

Drug-resistance-associated mutations in *M. tb* were obtained from the database TBDReaMDB and articles reporting rifampicin-resistance mutations (Supplementary Table 2). *M. tb* isolates carrying any characterized rifampicin-resistance mutation were identified as rifampicin-resistant strains, whereas those isolates with additional isoniazid-resistance mutations were determined as MDR-TB strains. To determine the drug-resistance spectra of the MDR-TB isolates in the Global dataset, we additionally identified drug-resistance-associated mutations for nine drugs (amikacin, capreomycin, ethambutol, ethionamide, fluoroquinolones, isoniazid, kanamycin, para-aminosalicylic acid, pyrazinamide, streptomycin) by inspection of their whole-genome sequences. Any isolates with genetic distances of less than 12 SNPs were classified into a cluster^10^. To compare the collection time distributions between the isolates in the clustered and nonclustered groups, we determined the collection time of each isolate as “days to the end of the study duration (31 December 2012)”. For example, if an isolate was collected on 1 January 2011, then its collection would be counted as “365 (days in 2010) + 366 (days in 2012) = 731 days”.

A Python script was written for screening transmission clusters in both the Shanghai and Global datasets, and this Python script was uploaded to GitHub.

### Statistical analysis

The Wilcoxon nonparametric rank-sum test and Student’s *t*-test were used to compare discrete variables between groups. Chi-square analysis was performed to compare the ratios of compensatory mutations in different groups. All statistical analyses were performed and visualized in RStudio (https://www.rstudio.com/).

### Ethical approval

The institutional review board of the Shanghai CDC approved the analysis with the anonymous dataset.

### Data availability

The analysis scripts that were written and used in this study are available online at GitHub (https://github.com/StopTB/Compen_Muta_Transmit).

## Electronic supplementary material

Supplementary tables
