# Supplementary material for: Have compensatory mutations facilitated the current epidemic of multidrug-resistant tuberculosis?
Source: Emerg Microbes Infect. 2018 Jun 6;7:98. doi: 10.1038/s41426-018-0101-6 (PMC5988693; doi:10.1038/s41426-018-0101-6)
Supplement: Supplementary file 1 — Supplementary tables [file 41426_2018_101_MOESM1_ESM.pdf]

Supplementary Table 1. Data information of the Global dataset.

Continent	Country	Title of the original article	Total strain number	No. of MDR-TB strains	Cohort or population-based collection
Europe	UK	**Whole-genome sequencing for prediction of Mycobacterium tuberculosis drug susceptibility and resistance: a retrospective cohort study.**<br>Whole-genome sequencing for rapid susceptibility testing of M. tuberculosis.<br>Whole-genome sequencing to delineate Mycobacterium tuberculosis outbreaks: a retrospective observational study.<br>Clinical application of whole-genome sequencing to inform treatment for multidrug-resistant tuberculosis cases.<br>Assessment of Mycobacterium tuberculosis transmission in Oxfordshire, UK, 2007-12, with whole pathogen genome sequences: an observational study.	1859	90	72
	Germany	**Whole-genome sequencing for prediction of Mycobacterium tuberculosis drug susceptibility and resistance: a retrospective cohort study.**<br>Whole-genome-based Mycobacterium tuberculosis surveillance: a standardized, portable, and expandable approach.	814	7	7
	Netherlands	Inferring patient to patient transmission of Mycobacterium tuberculosis from whole genome sequencing data.	207	2	
	Switzerland	Tracking a tuberculosis outbreak over 21 years: strain-specific single-nucleotide polymorphism typing combined with targeted whole-genome sequencing.	74	3	
	Russia	Evolution and transmission of drug resistant tuberculosis in a Russian population	1285	546	
Asia	China	Genome sequencing of 161 Mycobacterium tuberculosis isolates from China identifies genes and intergenic regions associated with drug resistance<br>MDR_cluster	283	99	
	Uzbekistan	**Whole-genome sequencing for prediction of Mycobacterium tuberculosis drug susceptibility and resistance: a retrospective cohort study.**	260	237	237
North America	Canada	Population genomics of Mycobacterium tuberculosis in the Inuit.<br>Marked microevolution of a unique Mycobacterium tuberculosis strain in 17 years of ongoing transmission in a high risk population.	221	0	
Africa	South-Africa	**Whole-genome sequencing for prediction of Mycobacterium tuberculosis drug susceptibility and resistance: a retrospective cohort study.**<br>Whole genome sequencing reveals genomic heterogeneity and antibiotic purification in Mycobacterium tuberculosis isolates.<br>**Evolution of Extensively Drug-Resistant Tuberculosis over Four Decades: Whole Genome Sequencing and Dating Analysis of Mycobacterium tuberculosis Isolates from KwaZulu-Natal.**	1021	284	276

Sierra Leone	**Whole-genome sequencing for prediction of Mycobacterium tuberculosis drug susceptibility and resistance: a retrospective cohort study.**	70	10	10
Malawi	Large-scale whole genome sequencing of M. tuberculosis provides insights into transmission in a high prevalence area	2268	33	
Mali	Whole Genome Sequencing of Mycobacterium africanum Strains from Mali Provides Insights into the Mechanisms of Geographic Restriction	91	35	

Supplementary Table 2. Drug-reistant mutations that were used to identify MDR-TB strains in this study.

Antibiotics	Drug resistant genes	Loci in H37Rv	Wild type	Mutant type	Codon change
RIFAMPICIN	rpoB	760314	G	T	V170F
RIFAMPICIN	rpoB	760663	C	T	A286V
RIFAMPICIN	rpoB	761004	A	G	T400A
RIFAMPICIN	rpoB	761033	G	T	Q409H
RIFAMPICIN	rpoB	761033	G	C	Q409H
RIFAMPICIN	rpoB	761074	A	C	E423A
RIFAMPICIN	rpoB	761076	T	C	F424L
RIFAMPICIN	rpoB	761076	T	G	F424V
RIFAMPICIN	rpoB	761077	T	C	F424S
RIFAMPICIN	rpoB	761078	C	A	F424L
RIFAMPICIN	rpoB	761078	C	G	F424L
RIFAMPICIN	rpoB	761082	G	A	G426S
RIFAMPICIN	rpoB	761083	G	A	G426D
RIFAMPICIN	rpoB	761085	A	T	T427S
RIFAMPICIN	rpoB	761085	A	C	T427P
RIFAMPICIN	rpoB	761085	A	G	T427A
RIFAMPICIN	rpoB	761086	C	G	T427S
RIFAMPICIN	rpoB	761088	A	C	S428R
RIFAMPICIN	rpoB	761089	G	T	S428I
RIFAMPICIN	rpoB	761089	G	C	S428T
RIFAMPICIN	rpoB	761090	C	A	S428R
RIFAMPICIN	rpoB	761090	C	G	S428R
RIFAMPICIN	rpoB	761091	C	A	Q429K
RIFAMPICIN	rpoB	761093	G	T	Q429H
RIFAMPICIN	rpoB	761093	G	C	Q429H
RIFAMPICIN	rpoB	761094	C	G	L430V
RIFAMPICIN	rpoB	761095	T	C	L430P
RIFAMPICIN	rpoB	761095	T	G	L430R
RIFAMPICIN	rpoB	761097	A	C	S431R
RIFAMPICIN	rpoB	761097	A	G	S431G
RIFAMPICIN	rpoB	761098	G	T	S431I
RIFAMPICIN	rpoB	761098	G	C	S431T
RIFAMPICIN	rpoB	761099	C	A	S431R
RIFAMPICIN	rpoB	761099	C	G	S431R
RIFAMPICIN	rpoB	761100	C	A	Q432K
RIFAMPICIN	rpoB	761100	C	T	Q432*
RIFAMPICIN	rpoB	761100	C	G	Q432E
RIFAMPICIN	rpoB	761101	A	T	Q432L
RIFAMPICIN	rpoB	761101	A	C	Q432P
RIFAMPICIN	rpoB	761101	A	G	Q432R
RIFAMPICIN	rpoB	761102	A	T	Q432H
RIFAMPICIN	rpoB	761102	A	C	Q432H
RIFAMPICIN	rpoB	761103	T	C	F433L
RIFAMPICIN	rpoB	761103	T	G	F433V
RIFAMPICIN	rpoB	761105	C	A	F433L
RIFAMPICIN	rpoB	761105	C	G	F433L
RIFAMPICIN	rpoB	761107	T	C	M434T
RIFAMPICIN	rpoB	761108	G	A	M434I

RIFAMPICIN	rpoB	761108	G	T	M434I
RIFAMPICIN	rpoB	761108	G	C	M434I
RIFAMPICIN	rpoB	761109	G	A	D435N
RIFAMPICIN	rpoB	761109	G	T	D435Y
RIFAMPICIN	rpoB	761109	G	C	D435H
RIFAMPICIN	rpoB	761110	A	T	D435V
RIFAMPICIN	rpoB	761110	A	C	D435A
RIFAMPICIN	rpoB	761110	A	G	D435G
RIFAMPICIN	rpoB	761111	C	A	D435E
RIFAMPICIN	rpoB	761111	C	G	D435E
RIFAMPICIN	rpoB	761113	A	T	Q436L
RIFAMPICIN	rpoB	761114	G	T	Q436H
RIFAMPICIN	rpoB	761114	G	C	Q436H
RIFAMPICIN	rpoB	761115	A	T	N437Y
RIFAMPICIN	rpoB	761115	A	C	N437H
RIFAMPICIN	rpoB	761115	A	G	N437D
RIFAMPICIN	rpoB	761116	A	T	N437I
RIFAMPICIN	rpoB	761116	A	C	N437T
RIFAMPICIN	rpoB	761120	C	A	N438K
RIFAMPICIN	rpoB	761120	C	G	N438K
RIFAMPICIN	rpoB	761124	C	A	L440M
RIFAMPICIN	rpoB	761125	T	C	L440P
RIFAMPICIN	rpoB	761128	C	T	S441L
RIFAMPICIN	rpoB	761128	C	G	S441W
RIFAMPICIN	rpoB	761130	G	T	G442W
RIFAMPICIN	rpoB	761131	G	C	G442A
RIFAMPICIN	rpoB	761134	T	C	L443S
RIFAMPICIN	rpoB	761134	T	G	L443W
RIFAMPICIN	rpoB	761136	A	C	T444P
RIFAMPICIN	rpoB	761137	C	T	T444I
RIFAMPICIN	rpoB	761139	C	A	H445N
RIFAMPICIN	rpoB	761139	C	T	H445Y
RIFAMPICIN	rpoB	761139	C	G	H445D
RIFAMPICIN	rpoB	761140	A	T	H445L
RIFAMPICIN	rpoB	761140	A	C	H445P
RIFAMPICIN	rpoB	761140	A	G	H445R
RIFAMPICIN	rpoB	761141	C	A	H445Q
RIFAMPICIN	rpoB	761141	C	G	H445Q
RIFAMPICIN	rpoB	761142	A	C	K446Q
RIFAMPICIN	rpoB	761144	G	T	K446N
RIFAMPICIN	rpoB	761144	G	C	K446N
RIFAMPICIN	rpoB	761146	G	A	R447H
RIFAMPICIN	rpoB	761146	G	C	R447P
RIFAMPICIN	rpoB	761155	C	T	S450L
RIFAMPICIN	rpoB	761155	C	G	S450W
RIFAMPICIN	rpoB	761161	T	C	L452P
RIFAMPICIN	rpoB	761166	C	T	P454S
RIFAMPICIN	rpoB	761167	C	A	P454H
RIFAMPICIN	rpoB	761167	C	T	P454L
RIFAMPICIN	rpoB	761167	C	G	P454R
RIFAMPICIN	rpoB	761176	T	C	L457P

RIFAMPICIN	rpoB	761185	A	G	E460G
RIFAMPICIN	rpoB	761196	C	A	L464M
RIFAMPICIN	rpoB	761220	T	G	S472A
RIFAMPICIN	rpoB	761244	A	G	I480V
RIFAMPICIN	rpoB	761245	T	C	I480T
RIFAMPICIN	rpoB	761248	A	G	E481G
RIFAMPICIN	rpoB	761250	A	C	T482P
RIFAMPICIN	rpoB	761254	C	T	P483L
RIFAMPICIN	rpoB	761274	C	G	L490V
RIFAMPICIN	rpoB	761277	A	T	I491F
RIFAMPICIN	rpoB	761284	C	T	S493L
RIFAMPICIN	rpoB	761460	C	T	R552C
RIFAMPICIN	rpoB	761582	G	T	E592D
RIFAMPICIN	rpoB	761582	G	C	E592D
RIFAMPICIN	rpoB	762089	G	T	E761D
RIFAMPICIN	rpoB	762089	G	C	E761D
ISONIAZID	ahpC	2726196	C	T	P2S
ISONIAZID	ahpC	2726206	C	T	T5I
ISONIAZID	ahpC	2726220	T	A	F10I
ISONIAZID	ahpC	2726289	G	A	D33N
ISONIAZID	ahpC	2726409	G	C	D73H
ISONIAZID	ahpC	2726418	G	A	E76K
ISONIAZID	ahpC	2726764	T	G	L191R
ISONIAZID	ahpC_promoter	2726112	C	T	C-81T
ISONIAZID	ahpC_promoter	2726119	G	A	G-74A
ISONIAZID	ahpC_promoter	2726127	G	A	G-66A
ISONIAZID	ahpC_promoter	2726139	C	T	C-54T
ISONIAZID	ahpC_promoter	2726141	C	T	C-52T
ISONIAZID	ahpC_promoter	2726142	G	T	G-51T
ISONIAZID	ahpC_promoter	2726144	T	G	T-49G
ISONIAZID	ahpC_promoter	2726145	G	A	G-48A
ISONIAZID	ahpC_promoter	2726145	G	T	G-48T
ISONIAZID	ahpC_promoter	2726149	T	A	T-44A
ISONIAZID	ahpC_promoter	2726154	C	T	C-39T
ISONIAZID	ahpC_promoter	2726173	C	T	C-20T
ISONIAZID	ahpC_promoter	2726188	G	A	G-5A
ISONIAZID	ahpC_promoter	2726189	A	G	A-4G
ISONIAZID	fabG1_promoter	1673348	A	T	A-92T
ISONIAZID	fabG1_promoter	1673373	G	C	G-67C
ISONIAZID	fabG1_promoter	1673416	G	T	G-24T
ISONIAZID	fabG1_promoter	1673423	G	T	G-17T
ISONIAZID	fabG1_promoter	1673424	A	G	A-16G
ISONIAZID	fabG1_promoter	1673425	C	T	C-15T
ISONIAZID	fabG1_promoter	1673429	A	T	A-11T
ISONIAZID	fabG1_promoter	1673432	T	A	T-8A
ISONIAZID	fabG1_promoter	1673432	T	C	T-8C
ISONIAZID	fabG1_promoter	1673432	T	G	T-8G
ISONIAZID	fabG1_promoter	1673435	T	A	T-5A
ISONIAZID	inhA	1674225	A	T	K8N
ISONIAZID	inhA	1674225	A	C	K8N
ISONIAZID	inhA	1674232	C	G	L11V

ISONIAZID	inhA	1674248	T	C	I16T
ISONIAZID	inhA	1674262	A	G	I21V
ISONIAZID	inhA	1674263	T	A	I21N
ISONIAZID	inhA	1674263	T	C	I21T
ISONIAZID	inhA	1674319	G	T	G40W
ISONIAZID	inhA	1674341	T	C	I47T
ISONIAZID	inhA	1674481	T	G	S94A
ISONIAZID	inhA	1674482	C	T	S94L
ISONIAZID	inhA	1674482	C	G	S94W
ISONIAZID	inhA	1674485	T	C	I95T
ISONIAZID	inhA	1674782	T	C	I194T
ISONIAZID	kasA	2518310	G	A	D66N
ISONIAZID	kasA	2518345	G	A	M77I
ISONIAZID	kasA	2518345	G	T	M77I
ISONIAZID	kasA	2518345	G	C	M77I
ISONIAZID	kasA	2518476	G	A	R121K
ISONIAZID	kasA	2518919	G	A	G269S
ISONIAZID	kasA	2519048	G	A	G312S
ISONIAZID	kasA	2519274	G	A	G387D
ISONIAZID	kasA	2519351	T	C	F413L
ISONIAZID	kasA	2519353	C	A	F413L
ISONIAZID	kasA	2519353	C	G	F413L
ISONIAZID	katG	2153908	T	G	D735A
ISONIAZID	katG	2153909	C	T	D735N
ISONIAZID	katG	2153928	C	A	W728C
ISONIAZID	katG	2153928	C	G	W728C
ISONIAZID	katG	2153932	G	T	A727D
ISONIAZID	katG	2153962	T	G	Q717P
ISONIAZID	katG	2153966	C	G	A716P
ISONIAZID	katG	2153975	C	G	A713P
ISONIAZID	katG	2153983	A	G	V710A
ISONIAZID	katG	2154014	A	G	S700P
ISONIAZID	katG	2154028	T	G	D695A
ISONIAZID	katG	2154059	C	G	G685R
ISONIAZID	katG	2154154	A	G	L653P
ISONIAZID	katG	2154205	G	T	A636E
ISONIAZID	katG	2154212	G	A	L634F
ISONIAZID	katG	2154227	C	T	G629S
ISONIAZID	katG	2154256	A	G	L619P
ISONIAZID	katG	2154285	C	A	M609I
ISONIAZID	katG	2154285	C	T	M609I
ISONIAZID	katG	2154285	C	G	M609I
ISONIAZID	katG	2154293	C	T	E607K
ISONIAZID	katG	2154334	C	T	G593D
ISONIAZID	katG	2154347	G	T	P589T
ISONIAZID	katG	2154352	A	G	L587P
ISONIAZID	katG	2154353	G	T	L587M
ISONIAZID	katG	2154391	G	A	A574V
ISONIAZID	katG	2154391	G	T	A574E
ISONIAZID	katG	2154395	C	T	D573N
ISONIAZID	katG	2154412	A	G	F567S

ISONIAZID	katG	2154460	G	C	A551G
ISONIAZID	katG	2154463	G	T	A550D
ISONIAZID	katG	2154527	T	C	N529D
ISONIAZID	katG	2154538	T	G	Q525P
ISONIAZID	katG	2154569	G	A	R515C
ISONIAZID	katG	2154598	C	G	W505S
ISONIAZID	katG	2154599	A	T	W505R
ISONIAZID	katG	2154599	A	G	W505R
ISONIAZID	katG	2154606	T	A	Q502H
ISONIAZID	katG	2154606	T	G	Q502H
ISONIAZID	katG	2154625	C	A	R496L
ISONIAZID	katG	2154641	C	A	G491C
ISONIAZID	katG	2154648	C	A	K488N
ISONIAZID	katG	2154648	C	G	K488N
ISONIAZID	katG	2154658	C	A	G485V
ISONIAZID	katG	2154662	G	T	R484S
ISONIAZID	katG	2154681	C	T	W477*
ISONIAZID	katG	2154682	C	T	W477*
ISONIAZID	katG	2154695	C	A	V473F
ISONIAZID	katG	2154722	C	A	A464S
ISONIAZID	katG	2154725	G	A	R463W
ISONIAZID	katG	2154727	A	G	I462T
ISONIAZID	katG	2154730	T	G	Q461P
ISONIAZID	katG	2154737	T	A	K459*
ISONIAZID	katG	2154742	C	A	S457I
ISONIAZID	katG	2154767	G	A	L449F
ISONIAZID	katG	2154782	C	T	A444T
ISONIAZID	katG	2154808	G	A	T435I
ISONIAZID	katG	2154812	G	A	Q434*
ISONIAZID	katG	2154827	G	A	P429S
ISONIAZID	katG	2154830	C	T	G428R
ISONIAZID	katG	2154830	C	G	G428R
ISONIAZID	katG	2154841	G	A	A424V
ISONIAZID	katG	2154841	G	T	A424E
ISONIAZID	katG	2154841	G	C	A424G
ISONIAZID	katG	2154857	C	G	D419H
ISONIAZID	katG	2154859	C	A	R418L
ISONIAZID	katG	2154859	C	T	R418Q
ISONIAZID	katG	2154870	C	A	K414N
ISONIAZID	katG	2154870	C	G	K414N
ISONIAZID	katG	2154875	A	G	Y413H
ISONIAZID	katG	2154886	G	T	A409D
ISONIAZID	katG	2154921	C	T	W397*
ISONIAZID	katG	2154922	C	T	W397*
ISONIAZID	katG	2154932	T	C	T394A
ISONIAZID	katG	2154934	A	T	I393N
ISONIAZID	katG	2154961	A	C	L384R
ISONIAZID	katG	2154970	T	C	D381G
ISONIAZID	katG	2154973	G	A	T380I
ISONIAZID	katG	2154976	G	A	A379V
ISONIAZID	katG	2154979	A	G	L378P

ISONIAZID	katG	2155018	G	A	P365L
ISONIAZID	katG	2155030	G	T	A361D
ISONIAZID	katG	2155043	C	T	D357N
ISONIAZID	katG	2155043	C	G	D357H
ISONIAZID	katG	2155058	G	A	Q352*
ISONIAZID	katG	2155064	C	A	A350S
ISONIAZID	katG	2155064	C	T	A350T
ISONIAZID	katG	2155078	T	G	K345T
ISONIAZID	katG	2155090	C	G	W341S
ISONIAZID	katG	2155102	T	A	Y337F
ISONIAZID	katG	2155102	T	C	Y337C
ISONIAZID	katG	2155105	A	C	L336R
ISONIAZID	katG	2155108	A	G	I335T
ISONIAZID	katG	2155109	T	C	I335V
ISONIAZID	katG	2155121	T	A	S331C
ISONIAZID	katG	2155128	C	A	W328C
ISONIAZID	katG	2155128	C	G	W328C
ISONIAZID	katG	2155129	C	A	W328L
ISONIAZID	katG	2155129	C	G	W328S
ISONIAZID	katG	2155130	A	C	W328G
ISONIAZID	katG	2155135	G	A	T326M
ISONIAZID	katG	2155142	T	G	T324P
ISONIAZID	katG	2155149	C	A	W321C
ISONIAZID	katG	2155149	C	T	W321*
ISONIAZID	katG	2155149	C	G	W321C
ISONIAZID	katG	2155150	C	A	W321L
ISONIAZID	katG	2155150	C	T	W321*
ISONIAZID	katG	2155150	C	G	W321S
ISONIAZID	katG	2155151	A	T	W321R
ISONIAZID	katG	2155151	A	G	W321R
ISONIAZID	katG	2155163	T	C	I317V
ISONIAZID	katG	2155163	T	G	I317L
ISONIAZID	katG	2155165	C	T	G316D
ISONIAZID	katG	2155166	C	T	G316S
ISONIAZID	katG	2155167	G	T	S315R
ISONIAZID	katG	2155167	G	C	S315R
ISONIAZID	katG	2155168	C	A	S315I
ISONIAZID	katG	2155168	C	T	S315N
ISONIAZID	katG	2155168	C	G	S315T
ISONIAZID	katG	2155169	T	C	S315G
ISONIAZID	katG	2155169	T	G	S315R
ISONIAZID	katG	2155179	G	T	D311E
ISONIAZID	katG	2155179	G	C	D311E
ISONIAZID	katG	2155180	T	C	D311G
ISONIAZID	katG	2155181	C	A	D311Y
ISONIAZID	katG	2155186	C	T	G309D
ISONIAZID	katG	2155187	C	A	G309C
ISONIAZID	katG	2155187	C	T	G309S
ISONIAZID	katG	2155190	T	G	T308P
ISONIAZID	katG	2155192	C	T	G307E
ISONIAZID	katG	2155192	C	G	G307A

ISONIAZID	katG	2155193	C	T	G307R
ISONIAZID	katG	2155193	C	G	G307R
ISONIAZID	katG	2155198	C	G	G305A
ISONIAZID	katG	2155201	T	G	Y304S
ISONIAZID	katG	2155206	G	T	S302R
ISONIAZID	katG	2155206	G	C	S302R
ISONIAZID	katG	2155208	T	G	S302R
ISONIAZID	katG	2155212	C	T	W300*
ISONIAZID	katG	2155213	C	T	W300*
ISONIAZID	katG	2155214	A	C	W300G
ISONIAZID	katG	2155216	C	G	G299A
ISONIAZID	katG	2155217	C	A	G299C
ISONIAZID	katG	2155222	C	A	G297V
ISONIAZID	katG	2155228	T	G	Q295P
ISONIAZID	katG	2155241	C	G	A291P
ISONIAZID	katG	2155245	C	A	E289D
ISONIAZID	katG	2155245	C	G	E289D
ISONIAZID	katG	2155258	C	T	G285D
ISONIAZID	katG	2155270	G	A	A281V
ISONIAZID	katG	2155276	C	T	G279D
ISONIAZID	katG	2155288	G	C	T275S
ISONIAZID	katG	2155289	T	A	T275S
ISONIAZID	katG	2155289	T	C	T275A
ISONIAZID	katG	2155289	T	G	T275P
ISONIAZID	katG	2155322	C	T	A264T
ISONIAZID	katG	2155327	G	C	T262R
ISONIAZID	katG	2155331	C	T	E261K
ISONIAZID	katG	2155335	G	T	D259E
ISONIAZID	katG	2155335	G	C	D259E
ISONIAZID	katG	2155339	T	C	N258S
ISONIAZID	katG	2155341	C	A	M257I
ISONIAZID	katG	2155341	C	T	M257I
ISONIAZID	katG	2155341	C	G	M257I
ISONIAZID	katG	2155342	A	G	M257T
ISONIAZID	katG	2155356	A	T	F252L
ISONIAZID	katG	2155356	A	C	F252L
ISONIAZID	katG	2155358	A	G	F252L
ISONIAZID	katG	2155360	G	A	T251M
ISONIAZID	katG	2155367	G	A	R249C
ISONIAZID	katG	2155385	C	A	A243S
ISONIAZID	katG	2155398	G	T	N238K
ISONIAZID	katG	2155398	G	C	N238K
ISONIAZID	katG	2155405	T	G	N236T
ISONIAZID	katG	2155411	C	T	G234E
ISONIAZID	katG	2155412	C	T	G234R
ISONIAZID	katG	2155412	C	G	G234R
ISONIAZID	katG	2155423	A	G	V230A
ISONIAZID	katG	2155426	T	A	Y229F
ISONIAZID	katG	2155442	G	C	Q224E
ISONIAZID	katG	2155458	G	T	N218K
ISONIAZID	katG	2155458	G	C	N218K

ISONIAZID	katG	2155514	T	A	K200*
ISONIAZID	katG	2155518	C	T	W198*
ISONIAZID	katG	2155519	C	T	W198*
ISONIAZID	katG	2155529	C	T	E195K
ISONIAZID	katG	2155541	A	T	W191R
ISONIAZID	katG	2155541	A	G	W191R
ISONIAZID	katG	2155555	C	A	G186V
ISONIAZID	katG	2155573	G	T	T180K
ISONIAZID	katG	2155584	C	A	M176I
ISONIAZID	katG	2155584	C	T	M176I
ISONIAZID	katG	2155584	C	G	M176I
ISONIAZID	katG	2155597	G	A	A172V
ISONIAZID	katG	2155598	C	T	A172T
ISONIAZID	katG	2155606	C	G	G169A
ISONIAZID	katG	2155628	C	T	A162T
ISONIAZID	katG	2155633	G	A	S160L
ISONIAZID	katG	2155648	T	C	Y155C
ISONIAZID	katG	2155648	T	G	Y155S
ISONIAZID	katG	2155669	A	C	L148R
ISONIAZID	katG	2155684	T	G	K143T
ISONIAZID	katG	2155687	T	G	D142A
ISONIAZID	katG	2155689	C	A	L141F
ISONIAZID	katG	2155689	C	G	L141F
ISONIAZID	katG	2155692	G	T	S140R
ISONIAZID	katG	2155692	G	C	S140R
ISONIAZID	katG	2155693	C	T	S140N
ISONIAZID	katG	2155694	T	C	S140G
ISONIAZID	katG	2155694	T	G	S140R
ISONIAZID	katG	2155697	C	G	A139P
ISONIAZID	katG	2155699	T	C	N138S
ISONIAZID	katG	2155699	T	G	N138T
ISONIAZID	katG	2155700	T	C	N138D
ISONIAZID	katG	2155700	T	G	N138H
ISONIAZID	katG	2155729	C	G	R128P
ISONIAZID	katG	2155732	T	G	Q127P
ISONIAZID	katG	2155734	C	A	M126I
ISONIAZID	katG	2155734	C	T	M126I
ISONIAZID	katG	2155734	C	G	M126I
ISONIAZID	katG	2155739	C	A	G125C
ISONIAZID	katG	2155750	C	A	G121V
ISONIAZID	katG	2155751	C	A	G121C
ISONIAZID	katG	2155762	T	G	D117A
ISONIAZID	katG	2155783	G	A	A110V
ISONIAZID	katG	2155786	G	A	A109V
ISONIAZID	katG	2155788	G	T	H108Q
ISONIAZID	katG	2155788	G	C	H108Q
ISONIAZID	katG	2155790	G	C	H108D
ISONIAZID	katG	2155791	C	T	W107*
ISONIAZID	katG	2155792	C	T	W107*
ISONIAZID	katG	2155793	A	T	W107R
ISONIAZID	katG	2155793	A	G	W107R

ISONIAZID	katG	2155795	G	A	A106V
ISONIAZID	katG	2155801	C	A	R104L
ISONIAZID	katG	2155801	C	T	R104Q
ISONIAZID	katG	2155816	C	T	G99E
ISONIAZID	katG	2155826	C	A	G96C
ISONIAZID	katG	2155831	T	C	D94G
ISONIAZID	katG	2155831	T	G	D94A
ISONIAZID	katG	2155841	A	T	W91R
ISONIAZID	katG	2155841	A	G	W91R
ISONIAZID	katG	2155842	C	T	W90*
ISONIAZID	katG	2155843	C	T	W90*
ISONIAZID	katG	2155849	T	C	Q88R
ISONIAZID	katG	2155859	T	G	T85P
ISONIAZID	katG	2155860	C	A	M84I
ISONIAZID	katG	2155860	C	T	M84I
ISONIAZID	katG	2155860	C	G	M84I
ISONIAZID	katG	2155891	T	C	D74G
ISONIAZID	katG	2155892	C	A	D74Y
ISONIAZID	katG	2155897	T	C	D72G
ISONIAZID	katG	2155900	A	T	I71N
ISONIAZID	katG	2155916	C	G	A66P
ISONIAZID	katG	2155919	C	T	A65T
ISONIAZID	katG	2155923	G	T	D63E
ISONIAZID	katG	2155923	G	C	D63E
ISONIAZID	katG	2155931	C	T	A61T
ISONIAZID	katG	2155969	A	T	L48Q
ISONIAZID	katG	2155998	C	T	W38*
ISONIAZID	katG	2155999	C	T	W38*
ISONIAZID	katG	2156009	T	C	N35D
ISONIAZID	katG	2156056	C	T	G19D
ISONIAZID	katG	2156062	C	T	S17N
ISONIAZID	katG	2156078	T	G	T12P
ISONIAZID	katG	2156081	T	C	T11A
ISONIAZID	katG	2156108	G	A	P2S
ISONIAZID	katG	2156110	A	G	V1A
ISONIAZID	katG	2156111	C	A	V1L
ISONIAZID	katG	2156111	C	G	V1L
ISONIAZID	katG_promoter	2156118	C	T	C-7T
ISONIAZID	katG_promoter	2156121	T	G	T-10G
ISONIAZID	katG_promoter	2156123	C	T	C-12T
ISONIAZID	inhA	1674434	T	C	V78A

Supplementary Table 3. The 1,468 MDR-TB strains that were included in study.

Strain ID	Rifampicin	Isoniazid	Country origin	Reference
2009_0645	rpoB_S450L	katG_S315T	China	Yang, C, et al. *The Lancet Infectious Diseases* 17.3 (2017): 275-284.
2009_0647	rpoB_H445Y	katG_S315T	China	Yang, C, et al. *The Lancet Infectious Diseases* 17.3 (2017): 275-284.
2009_0994	rpoB_S450L	fabG1_promoter_C1673425T	China	Yang, C, et al. *The Lancet Infectious Diseases* 17.3 (2017): 275-284.
2009_1011	rpoB_S450L	katG_S315T	China	Yang, C, et al. *The Lancet Infectious Diseases* 17.3 (2017): 275-284.
2009_1058	rpoB_S450L	katG_S315T	China	Yang, C, et al. *The Lancet Infectious Diseases* 17.3 (2017): 275-284.
2009_1060	rpoB_S450L	katG_S315T	China	Yang, C, et al. *The Lancet Infectious Diseases* 17.3 (2017): 275-284.
2009_1098	rpoB_S450L	katG_S315T	China	Yang, C, et al. *The Lancet Infectious Diseases* 17.3 (2017): 275-284.
2009_1121	rpoB_L430P,rpoB_H445Q	katG_S315T	China	Yang, C, et al. *The Lancet Infectious Diseases* 17.3 (2017): 275-284.
2009_116	rpoB_S450L	katG_S315T	China	Yang, C, et al. *The Lancet Infectious Diseases* 17.3 (2017): 275-284.
2009_1161	rpoB_S450L	katG_S315T	China	Yang, C, et al. *The Lancet Infectious Diseases* 17.3 (2017): 275-284.
2009_1303	rpoB_S450L	katG_S315T	China	Yang, C, et al. *The Lancet Infectious Diseases* 17.3 (2017): 275-284.
2009_1333	rpoB_S450L	katG_S315T	China	Yang, C, et al. *The Lancet Infectious Diseases* 17.3 (2017): 275-284.
2009_1413	rpoB_H445Y	katG_S315T	China	Yang, C, et al. *The Lancet Infectious Diseases* 17.3 (2017): 275-284.
2009_1543	rpoB_S450L	katG_S315T	China	Yang, C, et al. *The Lancet Infectious Diseases* 17.3 (2017): 275-284.
2009_1578	rpoB_S450L	ahpC_promoter_C2726141T,fabG1_promoter_C1673425T,katG_promoter_T2156121G	China	Yang, C, et al. *The Lancet Infectious Diseases* 17.3 (2017): 275-284.
2009_1585	rpoB_D435V	katG_S315T	China	Yang, C, et al. *The Lancet Infectious Diseases* 17.3 (2017): 275-284.
2009_1687	rpoB_S450L	katG_S315T	China	Yang, C, et al. *The Lancet Infectious Diseases* 17.3 (2017): 275-284.
2009_1780	rpoB_H445Y	katG_S315T	China	Yang, C, et al. *The Lancet Infectious Diseases* 17.3 (2017): 275-284.
2009_1862	rpoB_S450L	katG_S315T	China	Yang, C, et al. *The Lancet Infectious Diseases* 17.3 (2017): 275-284.
2009_1924	rpoB_S450L	katG_S315T	China	Yang, C, et al. *The Lancet Infectious Diseases* 17.3 (2017): 275-284.
2009_1928	rpoB_H445N	katG_S315T	China	Yang, C, et al. *The Lancet Infectious Diseases* 17.3 (2017): 275-284.
2009_1994	rpoB_H445N	katG_S315T	China	Yang, C, et al. *The Lancet Infectious Diseases* 17.3 (2017): 275-284.
2009_294	rpoB_S450L	katG_S315T	China	Yang, C, et al. *The Lancet Infectious Diseases* 17.3 (2017): 275-284.
2009_295	rpoB_S450L	katG_S315T	China	Yang, C, et al. *The Lancet Infectious Diseases* 17.3 (2017): 275-284.
2009_511	rpoB_Q432P	fabG1_promoter_C1673425T	China	Yang, C, et al. *The Lancet Infectious Diseases* 17.3 (2017): 275-284.
2009_620	rpoB_L430P,rpoB_Q432P	katG_S315T	China	Yang, C, et al. *The Lancet Infectious Diseases* 17.3 (2017): 275-284.
2009_643	rpoB_S450L	katG_S315T	China	Yang, C, et al. *The Lancet Infectious Diseases* 17.3 (2017): 275-284.
2009_682	rpoB_S450L	katG_S315T	China	Yang, C, et al. *The Lancet Infectious Diseases* 17.3 (2017): 275-284.
2009_716	rpoB_H445Y	katG_S315T	China	Yang, C, et al. *The Lancet Infectious Diseases* 17.3 (2017): 275-284.
2009_783	rpoB_L430P,rpoB_D435G	katG_S315T	China	Yang, C, et al. *The Lancet Infectious Diseases* 17.3 (2017): 275-284.
2009_784	rpoB_S450L	katG_S315T	China	Yang, C, et al. *The Lancet Infectious Diseases* 17.3 (2017): 275-284.
2009_799	rpoB_D435V	katG_S315T	China	Yang, C, et al. *The Lancet Infectious Diseases* 17.3 (2017): 275-284.
2009_817	rpoB_S450L	katG_S315T	China	Yang, C, et al. *The Lancet Infectious Diseases* 17.3 (2017): 275-284.
2009_956	rpoB_H445Y	katG_S315T	China	Yang, C, et al. *The Lancet Infectious Diseases* 17.3 (2017): 275-284.
2010_0020	rpoB_L430P,rpoB_S431G	katG_S315T,fabG1_promoter_T1673432C	China	Yang, C, et al. *The Lancet Infectious Diseases* 17.3 (2017): 275-284.
2010_0208	rpoB_S450L	katG_S315T	China	Yang, C, et al. *The Lancet Infectious Diseases* 17.3 (2017): 275-284.
2010_0560	rpoB_S450L	katG_S315T	China	Yang, C, et al. *The Lancet Infectious Diseases* 17.3 (2017): 275-284.
2010_0614	rpoB_H445Y	ahpC_promoter_G2726145A	China	Yang, C, et al. *The Lancet Infectious Diseases* 17.3 (2017): 275-284.
2010_0757	rpoB_L430P,rpoB_D435G	katG_S315T	China	Yang, C, et al. *The Lancet Infectious Diseases* 17.3 (2017): 275-284.
2010_0768	rpoB_H445Y	katG_S315T	China	Yang, C, et al. *The Lancet Infectious Diseases* 17.3 (2017): 275-284.
2010_0841	rpoB_S450L	katG_S315T	China	Yang, C, et al. *The Lancet Infectious Diseases* 17.3 (2017): 275-284.
2010_0888	rpoB_S450L	katG_S315T	China	Yang, C, et al. *The Lancet Infectious Diseases* 17.3 (2017): 275-284.
2010_1007	rpoB_S450L	katG_S315T	China	Yang, C, et al. *The Lancet Infectious Diseases* 17.3 (2017): 275-284.
2010_1180	rpoB_H445R	katG_S315T	China	Yang, C, et al. *The Lancet Infectious Diseases* 17.3 (2017): 275-284.
2010_122	rpoB_S450L	katG_S315T	China	Yang, C, et al. *The Lancet Infectious Diseases* 17.3 (2017): 275-284.
2010_1232	rpoB_S450L	katG_S315T	China	Yang, C, et al. *The Lancet Infectious Diseases* 17.3 (2017): 275-284.
2010_1603	rpoB_S450W	katG_S315T	China	Yang, C, et al. *The Lancet Infectious Diseases* 17.3 (2017): 275-284.
2010_1628	rpoB_D435Y	katG_S315T	China	Yang, C, et al. *The Lancet Infectious Diseases* 17.3 (2017): 275-284.
2010_1647	rpoB_D435V	katG_S315T	China	Yang, C, et al. *The Lancet Infectious Diseases* 17.3 (2017): 275-284.
2010_1730	rpoB_S450L	katG_S315T	China	Yang, C, et al. *The Lancet Infectious Diseases* 17.3 (2017): 275-284.
2010_1753	rpoB_L430P,rpoB_Q432P	katG_S315T	China	Yang, C, et al. *The Lancet Infectious Diseases* 17.3 (2017): 275-284.
2010_1770	rpoB_A286V,rpoB_S450L	katG_S315T,fabG1_promoter_T1673432C	China	Yang, C, et al. *The Lancet Infectious Diseases* 17.3 (2017): 275-284.
2010_1781	rpoB_L430P,rpoB_P454H	katG_S315T	China	Yang, C, et al. *The Lancet Infectious Diseases* 17.3 (2017): 275-284.
2010_183	rpoB_S450L	katG_S315T	China	Yang, C, et al. *The Lancet Infectious Diseases* 17.3 (2017): 275-284.

2010_1872	rpoB_S450L	katG_S315T	China	Yang, C, et al. *The Lancet Infectious Diseases* 17.3 (2017): 275-284.
2010_190	rpoB_S450L	katG_S315T	China	Yang, C, et al. *The Lancet Infectious Diseases* 17.3 (2017): 275-284.
2010_2094	rpoB_S450L	katG_Q295P	China	Yang, C, et al. *The Lancet Infectious Diseases* 17.3 (2017): 275-284.
2010_2132	rpoB_L430P,rpoB_Q432P	katG_S315T	China	Yang, C, et al. *The Lancet Infectious Diseases* 17.3 (2017): 275-284.
2010_2232	None	katG_S315T	China	Yang, C, et al. *The Lancet Infectious Diseases* 17.3 (2017): 275-284.
2010_2206	rpoB_H445Y	katG_S315T	China	Yang, C, et al. *The Lancet Infectious Diseases* 17.3 (2017): 275-284.
2010_2246	rpoB_H445Y	katG_S315G,fabG1_promoter_C1673425T	China	Yang, C, et al. *The Lancet Infectious Diseases* 17.3 (2017): 275-284.
2010_2303	rpoB_H445R	katG_S315T	China	Yang, C, et al. *The Lancet Infectious Diseases* 17.3 (2017): 275-284.
2010_297	rpoB_S450L	katG_S315T	China	Yang, C, et al. *The Lancet Infectious Diseases* 17.3 (2017): 275-284.
2010_360	rpoB_S450L	katG_S315T	China	Yang, C, et al. *The Lancet Infectious Diseases* 17.3 (2017): 275-284.
2010_381	rpoB_S450L	katG_S315T	China	Yang, C, et al. *The Lancet Infectious Diseases* 17.3 (2017): 275-284.
2010_390	rpoB_S450L	katG_S315T	China	Yang, C, et al. *The Lancet Infectious Diseases* 17.3 (2017): 275-284.
2010_539	rpoB_H445D	katG_S315T	China	Yang, C, et al. *The Lancet Infectious Diseases* 17.3 (2017): 275-284.
2010_54	rpoB_L452P	katG_S315T	China	Yang, C, et al. *The Lancet Infectious Diseases* 17.3 (2017): 275-284.
2010_592	rpoB_S450W	katG_S315T	China	Yang, C, et al. *The Lancet Infectious Diseases* 17.3 (2017): 275-284.
2010_600	rpoB_H445Y	katG_S315T	China	Yang, C, et al. *The Lancet Infectious Diseases* 17.3 (2017): 275-284.
2010_621	rpoB_S450L	katG_S315T	China	Yang, C, et al. *The Lancet Infectious Diseases* 17.3 (2017): 275-284.
2010_876	rpoB_S450L	katG_S315T	China	Yang, C, et al. *The Lancet Infectious Diseases* 17.3 (2017): 275-284.
2011_0293	None	katG_S315N	China	Yang, C, et al. *The Lancet Infectious Diseases* 17.3 (2017): 275-284.
2011_0295	rpoB_S450L	katG_S315T	China	Yang, C, et al. *The Lancet Infectious Diseases* 17.3 (2017): 275-284.
2011_0344	rpoB_S450L	fabG1_promoter_C1673425T	China	Yang, C, et al. *The Lancet Infectious Diseases* 17.3 (2017): 275-284.
2011_0370	None	katG_S315N	China	Yang, C, et al. *The Lancet Infectious Diseases* 17.3 (2017): 275-284.
2011_0426	rpoB_S450L	katG_S315T	China	Yang, C, et al. *The Lancet Infectious Diseases* 17.3 (2017): 275-284.
2011_0697	rpoB_H445R	ahpC_promoter_G2726145A	China	Yang, C, et al. *The Lancet Infectious Diseases* 17.3 (2017): 275-284.
2011_0870	rpoB_S450L	katG_S315T	China	Yang, C, et al. *The Lancet Infectious Diseases* 17.3 (2017): 275-284.
2011_1025	rpoB_S450L	katG_S315T	China	Yang, C, et al. *The Lancet Infectious Diseases* 17.3 (2017): 275-284.
2011_1081	rpoB_S431R,rpoB_D435Y	katG_S315T	China	Yang, C, et al. *The Lancet Infectious Diseases* 17.3 (2017): 275-284.
2011_12	rpoB_S450L	katG_S315T	China	Yang, C, et al. *The Lancet Infectious Diseases* 17.3 (2017): 275-284.
2011_1430	rpoB_S450L,rpoB_I480V	katG_S315N,ahpC_promoter_C2726112T	China	Yang, C, et al. *The Lancet Infectious Diseases* 17.3 (2017): 275-284.
2011_144	rpoB_S450L	katG_S315T	China	Yang, C, et al. *The Lancet Infectious Diseases* 17.3 (2017): 275-284.
2011_1549	rpoB_H445P	katG_S315T	China	Yang, C, et al. *The Lancet Infectious Diseases* 17.3 (2017): 275-284.
2011_1912	rpoB_S450L	katG_S315N	China	Yang, C, et al. *The Lancet Infectious Diseases* 17.3 (2017): 275-284.
2011_209	rpoB_L452P	katG_S315T	China	Yang, C, et al. *The Lancet Infectious Diseases* 17.3 (2017): 275-284.
2011_2094	rpoB_S450L	katG_S315N	China	Yang, C, et al. *The Lancet Infectious Diseases* 17.3 (2017): 275-284.
2011_2277	rpoB_L430P,rpoB_P454H	katG_S315T	China	Yang, C, et al. *The Lancet Infectious Diseases* 17.3 (2017): 275-284.
2011_277	rpoB_S450L	katG_S315T	China	Yang, C, et al. *The Lancet Infectious Diseases* 17.3 (2017): 275-284.
2011_417	rpoB_S450L	katG_S315T	China	Yang, C, et al. *The Lancet Infectious Diseases* 17.3 (2017): 275-284.
2011_431	rpoB_S450L	katG_S315T	China	Yang, C, et al. *The Lancet Infectious Diseases* 17.3 (2017): 275-284.
2011_644	rpoB_H445Y	katG_S315G,fabG1_promoter_C1673425T	China	Yang, C, et al. *The Lancet Infectious Diseases* 17.3 (2017): 275-284.
2011_867	rpoB_S450W	katG_S315T	China	Yang, C, et al. *The Lancet Infectious Diseases* 17.3 (2017): 275-284.
2012_0219	rpoB_S450L	katG_S315T	China	Yang, C, et al. *The Lancet Infectious Diseases* 17.3 (2017): 275-284.
2012_0043	None	katG_S315T	China	Yang, C, et al. *The Lancet Infectious Diseases* 17.3 (2017): 275-284.
2012_0242	rpoB_L430P,rpoB_H445Y	katG_S315T	China	Yang, C, et al. *The Lancet Infectious Diseases* 17.3 (2017): 275-284.
2012_0358	rpoB_A286V,rpoB_S450L	katG_S315T	China	Yang, C, et al. *The Lancet Infectious Diseases* 17.3 (2017): 275-284.
2012_0429	rpoB_L430P,rpoB_H445Y	katG_S315T	China	Yang, C, et al. *The Lancet Infectious Diseases* 17.3 (2017): 275-284.
2012_0444	rpoB_S450L	katG_S315T	China	Yang, C, et al. *The Lancet Infectious Diseases* 17.3 (2017): 275-284.
2012_0567	rpoB_S450L	katG_S315T	China	Yang, C, et al. *The Lancet Infectious Diseases* 17.3 (2017): 275-284.
2012_0569	rpoB_S450L	katG_S315T	China	Yang, C, et al. *The Lancet Infectious Diseases* 17.3 (2017): 275-284.
2012_0583	rpoB_S450L	katG_S315T	China	Yang, C, et al. *The Lancet Infectious Diseases* 17.3 (2017): 275-284.
2012_0603	rpoB_A286V,rpoB_S450L	katG_S315T	China	Yang, C, et al. *The Lancet Infectious Diseases* 17.3 (2017): 275-284.
2012_0659	rpoB_S450L	katG_S315T	China	Yang, C, et al. *The Lancet Infectious Diseases* 17.3 (2017): 275-284.
2012_0696	rpoB_S450L	katG_S315T	China	Yang, C, et al. *The Lancet Infectious Diseases* 17.3 (2017): 275-284.
2012_0701	rpoB_S450L	katG_S315T	China	Yang, C, et al. *The Lancet Infectious Diseases* 17.3 (2017): 275-284.
2012_0715	rpoB_S450L	katG_S315T	China	Yang, C, et al. *The Lancet Infectious Diseases* 17.3 (2017): 275-284.
2012_0779	rpoB_S450L	katG_S315N	China	Yang, C, et al. *The Lancet Infectious Diseases* 17.3 (2017): 275-284.
2012_1050	rpoB_S450L	katG_S315T	China	Yang, C, et al. *The Lancet Infectious Diseases* 17.3 (2017): 275-284.
2012_1055	rpoB_S450L	katG_S315T	China	Yang, C, et al. *The Lancet Infectious Diseases* 17.3 (2017): 275-284.
2012_1198	rpoB_S450L	katG_S315T	China	Yang, C, et al. *The Lancet Infectious Diseases* 17.3 (2017): 275-284.
2012_1484	rpoB_S450L	katG_S315N	China	Yang, C, et al. *The Lancet Infectious Diseases* 17.3 (2017): 275-284.
2012_1614	rpoB_S450L	katG_S315T	China	Yang, C, et al. *The Lancet Infectious Diseases* 17.3 (2017): 275-284.

2012_1655	rpoB_S450L,rpoB_I480V	fabG1_promoter_C1673425T	China	Yang, C, et al. *The Lancet Infectious Diseases* 17.3 (2017): 275-284.
2012_1810	rpoB_S450L	fabG1_promoter_C1673425T	China	Yang, C, et al. *The Lancet Infectious Diseases* 17.3 (2017): 275-284.
2012_1813	rpoB_S450L	katG_S315T	China	Yang, C, et al. *The Lancet Infectious Diseases* 17.3 (2017): 275-284.
2012_1828	rpoB_L430P,rpoB_S431G	katG_S315T,fabG1_promoter_T1673432C	China	Yang, C, et al. *The Lancet Infectious Diseases* 17.3 (2017): 275-284.
2012_2121	rpoB_H445Y	katG_S315T	China	Yang, C, et al. *The Lancet Infectious Diseases* 17.3 (2017): 275-284.
2012_2164	rpoB_S450L	katG_S315T	China	Yang, C, et al. *The Lancet Infectious Diseases* 17.3 (2017): 275-284.
2009_4	rpoB_H445D	katG_S315T	China	Yang, C, et al. *The Lancet Infectious Diseases* 17.3 (2017): 275-284.
2009_7	rpoB_S450L	katG_S315T	China	Yang, C, et al. *The Lancet Infectious Diseases* 17.3 (2017): 275-284.
SRR671719	rpoB_L452P	katG_S315N	China	Zhang, H, et al. Nature genetics 45.10 (2013): 1255-1260.
SRR671720	rpoB_H445L	fabG1_promoter_C1673425T	China	Zhang, H, et al. Nature genetics 45.10 (2013): 1255-1260.
SRR671721	rpoB_D435G,rpoB_H445D	katG_S315T	China	Zhang, H, et al. Nature genetics 45.10 (2013): 1255-1260.
SRR671722	rpoB_S450L	katG_S315T	China	Zhang, H, et al. Nature genetics 45.10 (2013): 1255-1260.
SRR671724	rpoB_S450L	fabG1_promoter_C1673425T	China	Zhang, H, et al. Nature genetics 45.10 (2013): 1255-1260.
SRR671725	rpoB_S450L	katG_S315N	China	Zhang, H, et al. Nature genetics 45.10 (2013): 1255-1260.
SRR671726	rpoB_S450L	ahpC_promoter_G2726119A	China	Zhang, H, et al. Nature genetics 45.10 (2013): 1255-1260.
SRR671731	rpoB_H445D	katG_S315T	China	Zhang, H, et al. Nature genetics 45.10 (2013): 1255-1260.
SRR671732	rpoB_S450L	katG_S315T	China	Zhang, H, et al. Nature genetics 45.10 (2013): 1255-1260.
SRR671733	rpoB_L452P	katG_S315N	China	Zhang, H, et al. Nature genetics 45.10 (2013): 1255-1260.
SRR671734	rpoB_H445D	katG_S315T	China	Zhang, H, et al. Nature genetics 45.10 (2013): 1255-1260.
SRR671735	rpoB_S450L	fabG1_promoter_C1673425T	China	Zhang, H, et al. Nature genetics 45.10 (2013): 1255-1260.
SRR671736	rpoB_S450L	katG_W191R,fabG1_promoter_C1673425T	China	Zhang, H, et al. Nature genetics 45.10 (2013): 1255-1260.
SRR671737	rpoB_S450L	ahpC_promoter_G2726145A	China	Zhang, H, et al. Nature genetics 45.10 (2013): 1255-1260.
SRR671738	rpoB_Q429H,rpoB_H445Y	katG_S315T	China	Zhang, H, et al. Nature genetics 45.10 (2013): 1255-1260.
SRR671739	rpoB_H445D	katG_S315T	China	Zhang, H, et al. Nature genetics 45.10 (2013): 1255-1260.
SRR671740	rpoB_H445Y	katG_V1A	China	Zhang, H, et al. Nature genetics 45.10 (2013): 1255-1260.
SRR671742	rpoB_H445D	katG_S315T	China	Zhang, H, et al. Nature genetics 45.10 (2013): 1255-1260.
SRR671743	rpoB_S450L	fabG1_promoter_C1673425T	China	Zhang, H, et al. Nature genetics 45.10 (2013): 1255-1260.
SRR671744	rpoB_S450L	katG_W191R,fabG1_promoter_C1673425T	China	Zhang, H, et al. Nature genetics 45.10 (2013): 1255-1260.
SRR671745	rpoB_H445Y	katG_S315T	China	Zhang, H, et al. Nature genetics 45.10 (2013): 1255-1260.
SRR671746	rpoB_H445Y	katG_S315T	China	Zhang, H, et al. Nature genetics 45.10 (2013): 1255-1260.
SRR671748	rpoB_L430R,rpoB_D435G	katG_S315N	China	Zhang, H, et al. Nature genetics 45.10 (2013): 1255-1260.
SRR671749	rpoB_H445R	katG_S315T	China	Zhang, H, et al. Nature genetics 45.10 (2013): 1255-1260.
SRR671751	rpoB_H445R	katG_S315T,fabG1_promoter_T1673432C	China	Zhang, H, et al. Nature genetics 45.10 (2013): 1255-1260.
SRR671752	rpoB_D435Y	katG_S315T	China	Zhang, H, et al. Nature genetics 45.10 (2013): 1255-1260.
SRR671753	rpoB_S450L	katG_S315T	China	Zhang, H, et al. Nature genetics 45.10 (2013): 1255-1260.
SRR671754	rpoB_S450L	katG_S315T	China	Zhang, H, et al. Nature genetics 45.10 (2013): 1255-1260.
SRR671755	rpoB_S450L	katG_S315T	China	Zhang, H, et al. Nature genetics 45.10 (2013): 1255-1260.
SRR671758	rpoB_H445R	ahpC_promoter_C2726141T	China	Zhang, H, et al. Nature genetics 45.10 (2013): 1255-1260.
SRR671759	rpoB_D435Y,rpoB_H445D	katG_S315T,fabG1_promoter_T1673432C	China	Zhang, H, et al. Nature genetics 45.10 (2013): 1255-1260.
SRR671760	rpoB_D435G,rpoB_L452P	katG_D419H	China	Zhang, H, et al. Nature genetics 45.10 (2013): 1255-1260.
SRR671761	rpoB_H445Y,rpoB_E460G	inhA_S94A,katG_S315T,fabG1_promoter_C1673425T	China	Zhang, H, et al. Nature genetics 45.10 (2013): 1255-1260.
SRR671765	rpoB_S441L	katG_S315T,fabG1_promoter_T1673432C	China	Zhang, H, et al. Nature genetics 45.10 (2013): 1255-1260.
SRR671766	rpoB_S450L	katG_S315T	China	Zhang, H, et al. Nature genetics 45.10 (2013): 1255-1260.
SRR671768	rpoB_S450L	katG_S315N	China	Zhang, H, et al. Nature genetics 45.10 (2013): 1255-1260.
SRR671769	rpoB_H445D	katG_Y155C	China	Zhang, H, et al. Nature genetics 45.10 (2013): 1255-1260.
SRR671770	rpoB_V170F	katG_L378P	China	Zhang, H, et al. Nature genetics 45.10 (2013): 1255-1260.
SRR671771	rpoB_L452P	katG_S315T	China	Zhang, H, et al. Nature genetics 45.10 (2013): 1255-1260.
SRR671774	rpoB_H445D	katG_Y155C	China	Zhang, H, et al. Nature genetics 45.10 (2013): 1255-1260.
SRR671779	rpoB_S450L	katG_S315T,fabG1_promoter_C1673425T	China	Zhang, H, et al. Nature genetics 45.10 (2013): 1255-1260.

SRR671781	rpoB_S450L	katG_W191R,inhA_I194T,fabG1_promoter_C1673425T	China	Zhang, H, et al. Nature genetics 45.10 (2013): 1255-1260.
SRR671785	rpoB_D435G,rpoB_L452P	katG_S315T	China	Zhang, H, et al. Nature genetics 45.10 (2013): 1255-1260.
SRR671787	rpoB_H445Y	katG_S315T	China	Zhang, H, et al. Nature genetics 45.10 (2013): 1255-1260.
SRR671789	rpoB_H445L	katG_S315T	China	Zhang, H, et al. Nature genetics 45.10 (2013): 1255-1260.
SRR671791	rpoB_D435V	katG_S315T	China	Zhang, H, et al. Nature genetics 45.10 (2013): 1255-1260.
SRR671794	rpoB_D435G,rpoB_L452P	katG_S315T	China	Zhang, H, et al. Nature genetics 45.10 (2013): 1255-1260.
SRR671795	rpoB_F424L,rpoB_L430P	katG_S315T	China	Zhang, H, et al. Nature genetics 45.10 (2013): 1255-1260.
SRR671796	rpoB_S450L	fabG1_promoter_C1673425T	China	Zhang, H, et al. Nature genetics 45.10 (2013): 1255-1260.
SRR671797	rpoB_S450L	katG_S315T	China	Zhang, H, et al. Nature genetics 45.10 (2013): 1255-1260.
SRR671798	rpoB_S450L	katG_S315T	China	Zhang, H, et al. Nature genetics 45.10 (2013): 1255-1260.
SRR671799	rpoB_S450L	fabG1_promoter_C1673425T	China	Zhang, H, et al. Nature genetics 45.10 (2013): 1255-1260.
SRR671801	rpoB_S450L	katG_S315T	China	Zhang, H, et al. Nature genetics 45.10 (2013): 1255-1260.
SRR671804	rpoB_D435A,rpoB_L452P	katG_S315T	China	Zhang, H, et al. Nature genetics 45.10 (2013): 1255-1260.
SRR671806	rpoB_L452P	katG_S315T	China	Zhang, H, et al. Nature genetics 45.10 (2013): 1255-1260.
SRR671807	rpoB_S450L	katG_S315T	China	Zhang, H, et al. Nature genetics 45.10 (2013): 1255-1260.
SRR671809	rpoB_S450L	katG_S315T	China	Zhang, H, et al. Nature genetics 45.10 (2013): 1255-1260.
SRR671810	rpoB_H445F	katG_S315N	China	Zhang, H, et al. Nature genetics 45.10 (2013): 1255-1260.
SRR671811	rpoB_S450L	katG_S315T	China	Zhang, H, et al. Nature genetics 45.10 (2013): 1255-1260.
SRR671812	rpoB_S450L	katG_S315T	China	Zhang, H, et al. Nature genetics 45.10 (2013): 1255-1260.
SRR671815	rpoB_S450L	katG_S315T,fabG1_promoter_T1673432G	China	Zhang, H, et al. Nature genetics 45.10 (2013): 1255-1260.
SRR671816	rpoB_H445D	katG_S315T	China	Zhang, H, et al. Nature genetics 45.10 (2013): 1255-1260.
SRR671817	rpoB_H445D	katG_S315T	China	Zhang, H, et al. Nature genetics 45.10 (2013): 1255-1260.
SRR671818	rpoB_S450L	katG_S315T	China	Zhang, H, et al. Nature genetics 45.10 (2013): 1255-1260.
SRR671819	rpoB_T427P,rpoB_L430P	katG_W191R,fabG1_promoter_C1673425T,ahpC_promoter_G2726145A	China	Zhang, H, et al. Nature genetics 45.10 (2013): 1255-1260.
SRR671820	rpoB_H445D	katG_S315T	China	Zhang, H, et al. Nature genetics 45.10 (2013): 1255-1260.
SRR671821	rpoB_S450L	katG_S315T	China	Zhang, H, et al. Nature genetics 45.10 (2013): 1255-1260.
SRR671822	rpoB_S450L	katG_S315T	China	Zhang, H, et al. Nature genetics 45.10 (2013): 1255-1260.
SRR671824	rpoB_S450L,rpoB_P454L	fabG1_promoter_C1673425T	China	Zhang, H, et al. Nature genetics 45.10 (2013): 1255-1260.
SRR671825	rpoB_S450L	katG_S315T	China	Zhang, H, et al. Nature genetics 45.10 (2013): 1255-1260.
SRR671826	rpoB_S450L	katG_T380I,ahpC_promoter_C2726139T,fabG1_promoter_C1673425T	China	Zhang, H, et al. Nature genetics 45.10 (2013): 1255-1260.
SRR671828	rpoB_H445D	katG_N138D,fabG1_promoter_T1673432A	China	Zhang, H, et al. Nature genetics 45.10 (2013): 1255-1260.
SRR671829	rpoB_S450L	katG_S315T	China	Zhang, H, et al. Nature genetics 45.10 (2013): 1255-1260.
SRR671831	rpoB_S450L	katG_S315T	China	Zhang, H, et al. Nature genetics 45.10 (2013): 1255-1260.
SRR671834	rpoB_S450L	katG_S315T	China	Zhang, H, et al. Nature genetics 45.10 (2013): 1255-1260.
SRR671837	rpoB_S450L	katG_S315T,ahpC_promoter_C2726141T	China	Zhang, H, et al. Nature genetics 45.10 (2013): 1255-1260.
SRR671838	rpoB_D435V	katG_S315T	China	Zhang, H, et al. Nature genetics 45.10 (2013): 1255-1260.
SRR671839	rpoB_H445Y	katG_S315T,fabG1_promoter_T1673432A	China	Zhang, H, et al. Nature genetics 45.10 (2013): 1255-1260.
SRR671840	rpoB_H445Y	katG_S315N	China	Zhang, H, et al. Nature genetics 45.10 (2013): 1255-1260.
SRR671841	rpoB_H445N,rpoB_P454L	katG_S315T	China	Zhang, H, et al. Nature genetics 45.10 (2013): 1255-1260.
SRR671843	rpoB_S450L	katG_S315T	China	Zhang, H, et al. Nature genetics 45.10 (2013): 1255-1260.
SRR671844	rpoB_S450L	katG_S315T,katG_S315R,katG_S315T	China	Zhang, H, et al. Nature genetics 45.10 (2013): 1255-1260.
SRR671848	rpoB_H445Y	katG_W191R,fabG1_promoter_C1673425T	China	Zhang, H, et al. Nature genetics 45.10 (2013): 1255-1260.
SRR671849	rpoB_S450L	katG_S315T	China	Zhang, H, et al. Nature genetics 45.10 (2013): 1255-1260.
SRR671850	rpoB_S450L	katG_S315T	China	Zhang, H, et al. Nature genetics 45.10 (2013): 1255-1260.
SRR671851	rpoB_L430P,rpoB_S431G	katG_S315T	China	Zhang, H, et al. Nature genetics 45.10 (2013): 1255-1260.
SRR671852	rpoB_H445Y	fabG1_promoter_C1673425T	China	Zhang, H, et al. Nature genetics 45.10 (2013): 1255-1260.
SRR671854	rpoB_S441L	katG_S315T	China	Zhang, H, et al. Nature genetics 45.10 (2013): 1255-1260.
SRR671855	rpoB_S450L	katG_S315T	China	Zhang, H, et al. Nature genetics 45.10 (2013): 1255-1260.
SRR671857	rpoB_S450L	katG_S315T	China	Zhang, H, et al. Nature genetics 45.10 (2013): 1255-1260.
SRR671861	rpoB_S450L	katG_S315N	China	Zhang, H, et al. Nature genetics 45.10 (2013): 1255-1260.

SRR671862	rpoB_S450L	katG_S315N	China	Zhang, H, et al. Nature genetics 45.10 (2013): 1255-1260.
SRR671863	rpoB_H445L	katG_S315T	China	Zhang, H, et al. Nature genetics 45.10 (2013): 1255-1260.
SRR671864	rpoB_H445L	katG_S315T	China	Zhang, H, et al. Nature genetics 45.10 (2013): 1255-1260.
SRR671865	rpoB_S450L	katG_S315T	China	Zhang, H, et al. Nature genetics 45.10 (2013): 1255-1260.
SRR671866	rpoB_S450L	katG_S315T	China	Zhang, H, et al. Nature genetics 45.10 (2013): 1255-1260.
SRR671867	rpoB_D435V	katG_Y155C,fabG1_promoter_C1673425T	China	Zhang, H, et al. Nature genetics 45.10 (2013): 1255-1260.
SRR671868	rpoB_S450L	fabG1_promoter_C1673425T	China	Zhang, H, et al. Nature genetics 45.10 (2013): 1255-1260.
SRR671869	rpoB_H445N	katG_S315G	China	Zhang, H, et al. Nature genetics 45.10 (2013): 1255-1260.
ERR550764	rpoB_S450L	katG_S315T	German	Walker, T.M., et al. The Lancet infectious diseases 15.10 (2015): 1193-1202.
ERR551236	rpoB_S450L	katG_S315T	German	Walker, T.M., et al. The Lancet infectious diseases 15.10 (2015): 1193-1202.
ERR552090	rpoB_S450L	katG_S315T	German	Walker, T.M., et al. The Lancet infectious diseases 15.10 (2015): 1193-1202.
ERR552326	rpoB_S450L	katG_S315T,katG_I335V	German	Walker, T.M., et al. The Lancet infectious diseases 15.10 (2015): 1193-1202.
ERR552391	rpoB_S450L	katG_S315T	German	Walker, T.M., et al. The Lancet infectious diseases 15.10 (2015): 1193-1202.
ERR552539	rpoB_L452P	katG_S315T	German	Walker, T.M., et al. The Lancet infectious diseases 15.10 (2015): 1193-1202.
ERR551957	rpoB_S450L	katG_S315T,katG_I335V	German	Walker, T.M., et al. The Lancet infectious diseases 15.10 (2015): 1193-1202.
ERR036188	rpoB_S450L	katG_S315T	Malawi	Guerra-Assunção, J. A., et al. Elife 4 (2015): e05166.
ERR037486	rpoB_S450L	katG_S315T	Malawi	Guerra-Assunção, J. A., et al. Elife 4 (2015): e05166.
ERR1063841	rpoB_S450L	inhA_I194T,fabG1_promoter_C1673425T	Malawi	Guerra-Assunção, J. A., et al. Elife 4 (2015): e05166.
ERR1063848	rpoB_S450L	katG_S315T	Malawi	Guerra-Assunção, J. A., et al. Elife 4 (2015): e05166.
ERR1063850	rpoB_D435V	katG_S315T	Malawi	Guerra-Assunção, J. A., et al. Elife 4 (2015): e05166.
ERR1063861	rpoB_H445N,rpoB_E460G	inhA_S94A	Malawi	Guerra-Assunção, J. A., et al. Elife 4 (2015): e05166.
ERR1063895	rpoB_H445Y	katG_S315T,fabG1_promoter_C1673425T	Malawi	Guerra-Assunção, J. A., et al. Elife 4 (2015): e05166.
ERR161085	rpoB_H445N	inhA_S94A	Malawi	Guerra-Assunção, J. A., et al. Elife 4 (2015): e05166.
ERR163953	rpoB_D435V	katG_S315T	Malawi	Guerra-Assunção, J. A., et al. Elife 4 (2015): e05166.
ERR163958	rpoB_S450L	katG_S315T	Malawi	Guerra-Assunção, J. A., et al. Elife 4 (2015): e05166.
ERR163962	rpoB_H445N	inhA_S94A	Malawi	Guerra-Assunção, J. A., et al. Elife 4 (2015): e05166.
ERR176629	rpoB_S450L	katG_S315T	Malawi	Guerra-Assunção, J. A., et al. Elife 4 (2015): e05166.
ERR181793	rpoB_H445N	inhA_S94A	Malawi	Guerra-Assunção, J. A., et al. Elife 4 (2015): e05166.
ERR181861	rpoB_S450L	katG_S315T	Malawi	Guerra-Assunção, J. A., et al. Elife 4 (2015): e05166.
ERR181930	rpoB_H445N	inhA_S94A	Malawi	Guerra-Assunção, J. A., et al. Elife 4 (2015): e05166.
ERR181933	rpoB_H445N	inhA_S94A	Malawi	Guerra-Assunção, J. A., et al. Elife 4 (2015): e05166.
ERR181986	rpoB_S450L	katG_S315T	Malawi	Guerra-Assunção, J. A., et al. Elife 4 (2015): e05166.
ERR190370	rpoB_S450L	katG_S315T	Malawi	Guerra-Assunção, J. A., et al. Elife 4 (2015): e05166.
ERR212021	rpoB_S450L	kasA_G269S,katG_S315T	Malawi	Guerra-Assunção, J. A., et al. Elife 4 (2015): e05166.
ERR212054	rpoB_H445N	inhA_S94A	Malawi	Guerra-Assunção, J. A., et al. Elife 4 (2015): e05166.
ERR221596	rpoB_H445N	inhA_S94A	Malawi	Guerra-Assunção, J. A., et al. Elife 4 (2015): e05166.
ERR221616	rpoB_S450L	katG_S315T	Malawi	Guerra-Assunção, J. A., et al. Elife 4 (2015): e05166.
ERR221617	rpoB_S450L	katG_S315T	Malawi	Guerra-Assunção, J. A., et al. Elife 4 (2015): e05166.
ERR221618	rpoB_S450L	katG_S315T	Malawi	Guerra-Assunção, J. A., et al. Elife 4 (2015): e05166.
ERR245651	rpoB_H445N	inhA_S94A	Malawi	Guerra-Assunção, J. A., et al. Elife 4 (2015): e05166.
ERR245670	rpoB_H445N	inhA_S94A	Malawi	Guerra-Assunção, J. A., et al. Elife 4 (2015): e05166.
ERR245829	rpoB_H445N	inhA_S94A	Malawi	Guerra-Assunção, J. A., et al. Elife 4 (2015): e05166.
ERR245836	rpoB_H445N	inhA_S94A	Malawi	Guerra-Assunção, J. A., et al. Elife 4 (2015): e05166.
ERR473327	rpoB_D435G,rpoB_L452P	kasA_G269S,katG_S315T,fabG1_promoter_T1673432A	Malawi	Guerra-Assunção, J. A., et al. Elife 4 (2015): e05166.
ERR736807	rpoB_H445N	inhA_S94A	Malawi	Guerra-Assunção, J. A., et al. Elife 4 (2015): e05166.
ERR736820	rpoB_H445N	inhA_S94A	Malawi	Guerra-Assunção, J. A., et al. Elife 4 (2015): e05166.
ERR773802	rpoB_H445N	inhA_S94A	Malawi	Guerra-Assunção, J. A., et al. Elife 4 (2015): e05166.
ERR779679	rpoB_H445N	inhA_S94A	Malawi	Guerra-Assunção, J. A., et al. Elife 4 (2015): e05166.
SRS485049	rpoB_S450L	inhA_V78A,katG_S315I	Mali	Winglee, K, et al. PLoS neglected tropical diseases 10.1 (2016): e0004332.
SRS485073	rpoB_L452P	katG_S315T	Mali	Winglee, K, et al. PLoS neglected tropical diseases 10.1 (2016): e0004332.
SRS485074	rpoB_S450L	katG_S315T	Mali	Winglee, K, et al. PLoS neglected tropical diseases 10.1 (2016): e0004332.
SRS485075	rpoB_D435V	katG_S315T	Mali	Winglee, K, et al. PLoS neglected tropical diseases 10.1 (2016): e0004332.
SRS485076	rpoB_D435V	katG_S315T	Mali	Winglee, K, et al. PLoS neglected tropical diseases 10.1 (2016): e0004332.
SRS485077	rpoB_S450L	inhA_I21T,fabG1_promoter_C1673425T	Mali	Winglee, K, et al. PLoS neglected tropical diseases 10.1 (2016): e0004332.
SRS485078	rpoB_L452P	katG_S315T	Mali	Winglee, K, et al. PLoS neglected tropical diseases 10.1 (2016): e0004332.
SRS485079	rpoB_D435V	katG_S315T	Mali	Winglee, K, et al. PLoS neglected tropical diseases 10.1 (2016): e0004332.

SRS485080	rpoB_L452P	katG_S315T	Mali	Winglee, K, et al. PLoS neglected tropical diseases 10.1 (2016): e0004332.
SRS485083	rpoB_S450L	katG_S315T	Mali	Winglee, K, et al. PLoS neglected tropical diseases 10.1 (2016): e0004332.
SRS485085	rpoB_H445D	katG_S315T	Mali	Winglee, K, et al. PLoS neglected tropical diseases 10.1 (2016): e0004332.
SRS485086	rpoB_D435V	inhA_S94A,katG_S315T	Mali	Winglee, K, et al. PLoS neglected tropical diseases 10.1 (2016): e0004332.
SRS485087	rpoB_H445D	katG_S315T	Mali	Winglee, K, et al. PLoS neglected tropical diseases 10.1 (2016): e0004332.
SRS485088	rpoB_D435V	katG_S315T	Mali	Winglee, K, et al. PLoS neglected tropical diseases 10.1 (2016): e0004332.
SRS485089	rpoB_S450L	katG_S315T	Mali	Winglee, K, et al. PLoS neglected tropical diseases 10.1 (2016): e0004332.
SRS485090	rpoB_D435V	katG_S315T	Mali	Winglee, K, et al. PLoS neglected tropical diseases 10.1 (2016): e0004332.
SRS485092	rpoB_D435V	katG_S315T,fabG1_promoter_T1673432C	Mali	Winglee, K, et al. PLoS neglected tropical diseases 10.1 (2016): e0004332.
SRS485093	rpoB_S450L	fabG1_promoter_C1673425T	Mali	Winglee, K, et al. PLoS neglected tropical diseases 10.1 (2016): e0004332.
SRS485094	rpoB_H445D	katG_S315T,fabG1_promoter_T1673432C	Mali	Winglee, K, et al. PLoS neglected tropical diseases 10.1 (2016): e0004332.
SRS485095	rpoB_D435V	katG_S315T	Mali	Winglee, K, et al. PLoS neglected tropical diseases 10.1 (2016): e0004332.
SRS485096	rpoB_H445Q,rpoB_L452P	katG_S315T	Mali	Winglee, K, et al. PLoS neglected tropical diseases 10.1 (2016): e0004332.
SRS485097	rpoB_H445D	katG_S315T,fabG1_promoter_T1673432C	Mali	Winglee, K, et al. PLoS neglected tropical diseases 10.1 (2016): e0004332.
SRS485098	rpoB_D435E,rpoB_S441L	katG_S315T	Mali	Winglee, K, et al. PLoS neglected tropical diseases 10.1 (2016): e0004332.
SRS485099	rpoB_S450L	katG_S315T	Mali	Winglee, K, et al. PLoS neglected tropical diseases 10.1 (2016): e0004332.
SRS526835	rpoB_H445L	katG_S315T,fabG1_promoter_T1673432C	Mali	Winglee, K, et al. PLoS neglected tropical diseases 10.1 (2016): e0004332.
SRS526842	rpoB_D435F	inhA_V78A,katG_S315T	Mali	Winglee, K, et al. PLoS neglected tropical diseases 10.1 (2016): e0004332.
SRS526897	rpoB_S450L	katG_W328L	Mali	Winglee, K, et al. PLoS neglected tropical diseases 10.1 (2016): e0004332.
SRS526924	rpoB_D435V	katG_S315T,fabG1_promoter_T1673432C	Mali	Winglee, K, et al. PLoS neglected tropical diseases 10.1 (2016): e0004332.
SRS526927	rpoB_S450L	katG_S315T	Mali	Winglee, K, et al. PLoS neglected tropical diseases 10.1 (2016): e0004332.
SRS526934	rpoB_H445D	katG_S315T,fabG1_promoter_T1673432C	Mali	Winglee, K, et al. PLoS neglected tropical diseases 10.1 (2016): e0004332.
SRS526977	rpoB_S450L	katG_S315T,fabG1_promoter_C1673425T	Mali	Winglee, K, et al. PLoS neglected tropical diseases 10.1 (2016): e0004332.
SRS526988	rpoB_H445R	katG_S315T	Mali	Winglee, K, et al. PLoS neglected tropical diseases 10.1 (2016): e0004332.
SRS703247	rpoB_S450L	katG_S315T	Mali	Winglee, K, et al. PLoS neglected tropical diseases 10.1 (2016): e0004332.
SRS703250	rpoB_D435V	katG_S315T	Mali	Winglee, K, et al. PLoS neglected tropical diseases 10.1 (2016): e0004332.
SRS703269	rpoB_S450L	inhA_V78A,katG_S315T	Mali	Winglee, K, et al. PLoS neglected tropical diseases 10.1 (2016): e0004332.
ERR024350	rpoB_S450L	katG_S315T	Netherlands	Bryant, J.M., et al. BMC infectious diseases 13.1 (2013): 110.
ERR024351	rpoB_S450L	katG_S315T	Netherlands	Bryant, J.M., et al. BMC infectious diseases 13.1 (2013): 110.
ERR015610	rpoB_S450L	katG_S315T	Russia	Casali, N, et al. Nature genetics 46.3 (2014): 279-286.
ERR015611	rpoB_S450L	katG_S315T	Russia	Casali, N, et al. Nature genetics 46.3 (2014): 279-286.
ERR015614	rpoB_S450L	katG_S315T	Russia	Casali, N, et al. Nature genetics 46.3 (2014): 279-286.
ERR015616	rpoB_L430P,rpoB_S431G,rpoB_H445Q	katG_S315T	Russia	Casali, N, et al. Nature genetics 46.3 (2014): 279-286.
ERR047880	rpoB_S450L	katG_S315T,fabG1_promoter_C1673425T	Russia	Casali, N, et al. Nature genetics 46.3 (2014): 279-286.
ERR047881	rpoB_S450L	kasA_G269S,katG_S315T	Russia	Casali, N, et al. Nature genetics 46.3 (2014): 279-286.
ERR047882	rpoB_H445Y	katG_S315T,fabG1_promoter_T1673432C	Russia	Casali, N, et al. Nature genetics 46.3 (2014): 279-286.
ERR047883	rpoB_S450L	kasA_G269S,katG_S315T	Russia	Casali, N, et al. Nature genetics 46.3 (2014): 279-286.
ERR047884	rpoB_S450L	katG_S315T,fabG1_promoter_C1673425T	Russia	Casali, N, et al. Nature genetics 46.3 (2014): 279-286.
ERR047885	rpoB_H445Y	katG_S315T,fabG1_promoter_T1673432C	Russia	Casali, N, et al. Nature genetics 46.3 (2014): 279-286.
ERR047886	rpoB_L452P	katG_S315T	Russia	Casali, N, et al. Nature genetics 46.3 (2014): 279-286.
ERR047887	rpoB_H445Y	katG_S315T,fabG1_promoter_T1673432C	Russia	Casali, N, et al. Nature genetics 46.3 (2014): 279-286.
ERR047888	rpoB_H445Y	katG_S315T,fabG1_promoter_T1673432C	Russia	Casali, N, et al. Nature genetics 46.3 (2014): 279-286.
ERR047889	rpoB_H445Y	katG_S315T,fabG1_promoter_T1673432C	Russia	Casali, N, et al. Nature genetics 46.3 (2014): 279-286.
ERR047890	rpoB_H445Y	katG_S315T,fabG1_promoter_T1673432C	Russia	Casali, N, et al. Nature genetics 46.3 (2014): 279-286.
ERR047891	rpoB_L452P	katG_S315T	Russia	Casali, N, et al. Nature genetics 46.3 (2014): 279-286.
ERR067576	rpoB_S450L	katG_S315T	Russia	Casali, N, et al. Nature genetics 46.3 (2014): 279-286.
ERR067577	rpoB_S450L	katG_S315T	Russia	Casali, N, et al. Nature genetics 46.3 (2014): 279-286.
ERR067578	rpoB_S450L	katG_S315T	Russia	Casali, N, et al. Nature genetics 46.3 (2014): 279-286.

ERR067581	rpoB_S450L	katG_S315T	Russia	Casali, N, et al. Nature genetics 46.3 (2014): 279-286.
ERR067583	rpoB_S450L	katG_S315T	Russia	Casali, N, et al. Nature genetics 46.3 (2014): 279-286.
ERR067584	rpoB_S450L	katG_S315T	Russia	Casali, N, et al. Nature genetics 46.3 (2014): 279-286.
ERR067587	rpoB_S450L	katG_S315T	Russia	Casali, N, et al. Nature genetics 46.3 (2014): 279-286.
ERR067588	rpoB_S450L	katG_S315T	Russia	Casali, N, et al. Nature genetics 46.3 (2014): 279-286.
ERR067590	rpoB_S450L	katG_S315T	Russia	Casali, N, et al. Nature genetics 46.3 (2014): 279-286.
ERR067592	rpoB_S450L	katG_S315T	Russia	Casali, N, et al. Nature genetics 46.3 (2014): 279-286.
ERR067593	rpoB_S450L	fabG1_promoter_C1673425T	Russia	Casali, N, et al. Nature genetics 46.3 (2014): 279-286.
ERR067598	rpoB_S450L	katG_S315T	Russia	Casali, N, et al. Nature genetics 46.3 (2014): 279-286.
ERR067599	rpoB_H445L	kasA_G269S,katG_S315T,fabG1_promoter_C1673425T	Russia	Casali, N, et al. Nature genetics 46.3 (2014): 279-286.
ERR067603	rpoB_S450L	katG_S315T,katG_S315R,katG_S315T	Russia	Casali, N, et al. Nature genetics 46.3 (2014): 279-286.
ERR067605	rpoB_S450L	katG_S315T	Russia	Casali, N, et al. Nature genetics 46.3 (2014): 279-286.
ERR067609	rpoB_S450L	katG_S315T	Russia	Casali, N, et al. Nature genetics 46.3 (2014): 279-286.
ERR067612	rpoB_Q432P	kasA_G269S	Russia	Casali, N, et al. Nature genetics 46.3 (2014): 279-286.
ERR067613	rpoB_S450L	katG_S315T	Russia	Casali, N, et al. Nature genetics 46.3 (2014): 279-286.
ERR067615	rpoB_S450L	katG_S315T	Russia	Casali, N, et al. Nature genetics 46.3 (2014): 279-286.
ERR067618	rpoB_S450L	katG_S315T	Russia	Casali, N, et al. Nature genetics 46.3 (2014): 279-286.
ERR067619	rpoB_S450L	katG_S315T	Russia	Casali, N, et al. Nature genetics 46.3 (2014): 279-286.
ERR067620	rpoB_S450L	katG_S315T	Russia	Casali, N, et al. Nature genetics 46.3 (2014): 279-286.
ERR067621	rpoB_S450L	katG_S315T	Russia	Casali, N, et al. Nature genetics 46.3 (2014): 279-286.
ERR067622	rpoB_S450L	katG_S315T	Russia	Casali, N, et al. Nature genetics 46.3 (2014): 279-286.
ERR067623	rpoB_S450L	katG_S315T	Russia	Casali, N, et al. Nature genetics 46.3 (2014): 279-286.
ERR067625	rpoB_S450L	katG_S315T	Russia	Casali, N, et al. Nature genetics 46.3 (2014): 279-286.
ERR067626	rpoB_S450L	katG_S315T	Russia	Casali, N, et al. Nature genetics 46.3 (2014): 279-286.
ERR067627	rpoB_S450W	katG_S315T	Russia	Casali, N, et al. Nature genetics 46.3 (2014): 279-286.
ERR067628	rpoB_S450L	katG_S315T	Russia	Casali, N, et al. Nature genetics 46.3 (2014): 279-286.
ERR067629	rpoB_S450L	katG_S315T	Russia	Casali, N, et al. Nature genetics 46.3 (2014): 279-286.
ERR067630	rpoB_S450L	katG_S315T	Russia	Casali, N, et al. Nature genetics 46.3 (2014): 279-286.
ERR067635	rpoB_S450L	katG_S315T	Russia	Casali, N, et al. Nature genetics 46.3 (2014): 279-286.
ERR067641	rpoB_S450L	katG_S315T	Russia	Casali, N, et al. Nature genetics 46.3 (2014): 279-286.
ERR067643	rpoB_S450L	katG_S315T	Russia	Casali, N, et al. Nature genetics 46.3 (2014): 279-286.
ERR067644	rpoB_S450L	katG_S315T	Russia	Casali, N, et al. Nature genetics 46.3 (2014): 279-286.
ERR067645	rpoB_H445D	katG_S315T,fabG1_promoter_C1673425T	Russia	Casali, N, et al. Nature genetics 46.3 (2014): 279-286.
ERR067647	rpoB_S450L	katG_S315T,katG_I335V	Russia	Casali, N, et al. Nature genetics 46.3 (2014): 279-286.
ERR067649	rpoB_S450L	katG_S315T	Russia	Casali, N, et al. Nature genetics 46.3 (2014): 279-286.
ERR067650	rpoB_S450L	katG_S315T	Russia	Casali, N, et al. Nature genetics 46.3 (2014): 279-286.
ERR067651	rpoB_S450L	katG_S315T	Russia	Casali, N, et al. Nature genetics 46.3 (2014): 279-286.
ERR067652	rpoB_S450L	katG_S315T	Russia	Casali, N, et al. Nature genetics 46.3 (2014): 279-286.
ERR067653	rpoB_S450L	katG_S315T	Russia	Casali, N, et al. Nature genetics 46.3 (2014): 279-286.
ERR067654	rpoB_S450L	katG_S315T	Russia	Casali, N, et al. Nature genetics 46.3 (2014): 279-286.
ERR067656	rpoB_S450L	katG_S315T	Russia	Casali, N, et al. Nature genetics 46.3 (2014): 279-286.
ERR067657	rpoB_S450L	katG_S315T	Russia	Casali, N, et al. Nature genetics 46.3 (2014): 279-286.
ERR067659	rpoB_S450L	katG_S315T	Russia	Casali, N, et al. Nature genetics 46.3 (2014): 279-286.
ERR067660	rpoB_S450L	katG_S315T	Russia	Casali, N, et al. Nature genetics 46.3 (2014): 279-286.
ERR067662	rpoB_S450L	katG_S315T	Russia	Casali, N, et al. Nature genetics 46.3 (2014): 279-286.
ERR067663	rpoB_S450L	katG_S315T	Russia	Casali, N, et al. Nature genetics 46.3 (2014): 279-286.
ERR067665	rpoB_S450L	katG_S315T	Russia	Casali, N, et al. Nature genetics 46.3 (2014): 279-286.
ERR067667	rpoB_S450L	katG_S315T	Russia	Casali, N, et al. Nature genetics 46.3 (2014): 279-286.
ERR067668	rpoB_S450L	katG_S315T	Russia	Casali, N, et al. Nature genetics 46.3 (2014): 279-286.
ERR067671	rpoB_S450L	katG_S315T,fabG1_promoter_T1673432C	Russia	Casali, N, et al. Nature genetics 46.3 (2014): 279-286.
ERR067672	rpoB_S450L	katG_S315T	Russia	Casali, N, et al. Nature genetics 46.3 (2014): 279-286.
ERR067673	rpoB_S450L	katG_S315T	Russia	Casali, N, et al. Nature genetics 46.3 (2014): 279-286.
ERR067674	rpoB_S450L	katG_S315T	Russia	Casali, N, et al. Nature genetics 46.3 (2014): 279-286.
ERR067677	rpoB_S450L	katG_S315T,fabG1_promoter_C1673425T	Russia	Casali, N, et al. Nature genetics 46.3 (2014): 279-286.
ERR067679	rpoB_S450L	kasA_G269S,katG_S315T,fabG1_promoter_C1673425T	Russia	Casali, N, et al. Nature genetics 46.3 (2014): 279-286.
ERR067687	rpoB_S450L	katG_S315T	Russia	Casali, N, et al. Nature genetics 46.3 (2014): 279-286.

ERR067691	rpoB_S450L	katG_S315T	Russia	Casali, N, et al. Nature genetics 46.3 (2014): 279-286.
ERR067692	rpoB_S450L	katG_S315T	Russia	Casali, N, et al. Nature genetics 46.3 (2014): 279-286.
ERR067694	rpoB_S450L	katG_S315T	Russia	Casali, N, et al. Nature genetics 46.3 (2014): 279-286.
ERR067695	rpoB_S450L	katG_S315T	Russia	Casali, N, et al. Nature genetics 46.3 (2014): 279-286.
ERR067696	rpoB_S450L	katG_S315T	Russia	Casali, N, et al. Nature genetics 46.3 (2014): 279-286.
ERR067698	rpoB_S450L	katG_S315T	Russia	Casali, N, et al. Nature genetics 46.3 (2014): 279-286.
ERR067705	rpoB_S450L	katG_S315T	Russia	Casali, N, et al. Nature genetics 46.3 (2014): 279-286.
ERR067707	rpoB_S450L	katG_S315T	Russia	Casali, N, et al. Nature genetics 46.3 (2014): 279-286.
ERR067709	rpoB_S450L	katG_S315T,fabG1_promoter_C1673425T	Russia	Casali, N, et al. Nature genetics 46.3 (2014): 279-286.
ERR067710	rpoB_S450L	katG_S315T	Russia	Casali, N, et al. Nature genetics 46.3 (2014): 279-286.
ERR067711	rpoB_S450L	katG_S315T	Russia	Casali, N, et al. Nature genetics 46.3 (2014): 279-286.
ERR067714	rpoB_S450L	katG_S315T	Russia	Casali, N, et al. Nature genetics 46.3 (2014): 279-286.
ERR067717	rpoB_S450L	katG_S315T	Russia	Casali, N, et al. Nature genetics 46.3 (2014): 279-286.
ERR067718	rpoB_S450L	katG_S315T	Russia	Casali, N, et al. Nature genetics 46.3 (2014): 279-286.
ERR067720	rpoB_S450L	katG_S315T	Russia	Casali, N, et al. Nature genetics 46.3 (2014): 279-286.
ERR067721	rpoB_S450L	katG_S315T	Russia	Casali, N, et al. Nature genetics 46.3 (2014): 279-286.
ERR067722	rpoB_S450L	fabG1_promoter_C1673425T	Russia	Casali, N, et al. Nature genetics 46.3 (2014): 279-286.
ERR067723	rpoB_S450L	katG_S315T	Russia	Casali, N, et al. Nature genetics 46.3 (2014): 279-286.
ERR067724	rpoB_S450L	inhA_S94A,fabG1_promoter_C1673425T	Russia	Casali, N, et al. Nature genetics 46.3 (2014): 279-286.
ERR067728	rpoB_S450L	katG_S315T	Russia	Casali, N, et al. Nature genetics 46.3 (2014): 279-286.
ERR067733	rpoB_S450L	katG_S315T	Russia	Casali, N, et al. Nature genetics 46.3 (2014): 279-286.
ERR067735	rpoB_S450L	katG_S315N	Russia	Casali, N, et al. Nature genetics 46.3 (2014): 279-286.
ERR067739	rpoB_S450L	katG_S315T	Russia	Casali, N, et al. Nature genetics 46.3 (2014): 279-286.
ERR067741	rpoB_S450L	katG_S315T	Russia	Casali, N, et al. Nature genetics 46.3 (2014): 279-286.
ERR067742	rpoB_S450L	katG_S315T	Russia	Casali, N, et al. Nature genetics 46.3 (2014): 279-286.
ERR067744	rpoB_S450L	katG_S315T	Russia	Casali, N, et al. Nature genetics 46.3 (2014): 279-286.
ERR067745	rpoB_L430P	katG_S315T	Russia	Casali, N, et al. Nature genetics 46.3 (2014): 279-286.
ERR067748	rpoB_S450L	katG_S315T	Russia	Casali, N, et al. Nature genetics 46.3 (2014): 279-286.
ERR067749	rpoB_S450L	katG_S315T	Russia	Casali, N, et al. Nature genetics 46.3 (2014): 279-286.
ERR067750	rpoB_S450L	katG_S315T	Russia	Casali, N, et al. Nature genetics 46.3 (2014): 279-286.
ERR067752	rpoB_S450L	katG_S315T,fabG1_promoter_C1673425T	Russia	Casali, N, et al. Nature genetics 46.3 (2014): 279-286.
ERR067754	rpoB_S450L	katG_S315T	Russia	Casali, N, et al. Nature genetics 46.3 (2014): 279-286.
ERR067755	rpoB_S450L	katG_S315T	Russia	Casali, N, et al. Nature genetics 46.3 (2014): 279-286.
ERR067758	rpoB_S450L	katG_S315T	Russia	Casali, N, et al. Nature genetics 46.3 (2014): 279-286.
ERR067760	rpoB_S450L	katG_S315T	Russia	Casali, N, et al. Nature genetics 46.3 (2014): 279-286.
ERR067761	rpoB_S450L	katG_S315T,fabG1_promoter_C1673425T	Russia	Casali, N, et al. Nature genetics 46.3 (2014): 279-286.
ERR067762	rpoB_S450L	katG_S315T	Russia	Casali, N, et al. Nature genetics 46.3 (2014): 279-286.
ERR067764	rpoB_S450L	katG_S315T	Russia	Casali, N, et al. Nature genetics 46.3 (2014): 279-286.
ERR067765	rpoB_S450L	katG_S315T	Russia	Casali, N, et al. Nature genetics 46.3 (2014): 279-286.
ERR108420	rpoB_S450L	katG_S315T,ahpC_promoter_G2726145A	Russia	Casali, N, et al. Nature genetics 46.3 (2014): 279-286.
ERR108421	rpoB_S450L	katG_S315T	Russia	Casali, N, et al. Nature genetics 46.3 (2014): 279-286.
ERR108422	rpoB_D435Y	kasA_G269S,katG_S315T,fabG1_promoter_T1673432A	Russia	Casali, N, et al. Nature genetics 46.3 (2014): 279-286.
ERR108425	rpoB_S450L	katG_S315T	Russia	Casali, N, et al. Nature genetics 46.3 (2014): 279-286.
ERR108426	rpoB_S450L	katG_S315T	Russia	Casali, N, et al. Nature genetics 46.3 (2014): 279-286.
ERR108428	rpoB_S450L	katG_S315T	Russia	Casali, N, et al. Nature genetics 46.3 (2014): 279-286.
ERR108435	rpoB_S450L	katG_S315T	Russia	Casali, N, et al. Nature genetics 46.3 (2014): 279-286.
ERR108436	rpoB_S450L	katG_S315T	Russia	Casali, N, et al. Nature genetics 46.3 (2014): 279-286.
ERR108439	rpoB_S450L	katG_S315T	Russia	Casali, N, et al. Nature genetics 46.3 (2014): 279-286.
ERR108443	rpoB_S450L	katG_S315T	Russia	Casali, N, et al. Nature genetics 46.3 (2014): 279-286.
ERR108450	rpoB_S450L	katG_S315T	Russia	Casali, N, et al. Nature genetics 46.3 (2014): 279-286.
ERR108453	rpoB_S450L	katG_S315T	Russia	Casali, N, et al. Nature genetics 46.3 (2014): 279-286.
ERR108454	rpoB_S450L	katG_S315T	Russia	Casali, N, et al. Nature genetics 46.3 (2014): 279-286.
ERR108455	rpoB_S450L	katG_S315T	Russia	Casali, N, et al. Nature genetics 46.3 (2014): 279-286.
ERR108458	rpoB_S450L	katG_S315T	Russia	Casali, N, et al. Nature genetics 46.3 (2014): 279-286.
ERR108462	rpoB_S450L	katG_S315T	Russia	Casali, N, et al. Nature genetics 46.3 (2014): 279-286.
ERR108467	rpoB_S450L	katG_S315T	Russia	Casali, N, et al. Nature genetics 46.3 (2014): 279-286.
ERR108469	rpoB_S450L	katG_S315T	Russia	Casali, N, et al. Nature genetics 46.3 (2014): 279-286.
ERR108473	rpoB_S450L	katG_S315T	Russia	Casali, N, et al. Nature genetics 46.3 (2014): 279-286.

ERR108474	rpoB_S450L	katG_S315T,katG_S315R,katG_S315T	Russia	Casali, N, et al. Nature genetics 46.3 (2014): 279-286.
ERR108475	rpoB_S450L	katG_S315T	Russia	Casali, N, et al. Nature genetics 46.3 (2014): 279-286.
ERR108477	rpoB_S450L	katG_S315T	Russia	Casali, N, et al. Nature genetics 46.3 (2014): 279-286.
ERR108480	rpoB_S450L	katG_S315T,katG_S315R,katG_S315T	Russia	Casali, N, et al. Nature genetics 46.3 (2014): 279-286.
ERR108482	rpoB_S450L	katG_S315T	Russia	Casali, N, et al. Nature genetics 46.3 (2014): 279-286.
ERR108484	rpoB_S450L	katG_S315T	Russia	Casali, N, et al. Nature genetics 46.3 (2014): 279-286.
ERR108488	rpoB_S450L	katG_S315T	Russia	Casali, N, et al. Nature genetics 46.3 (2014): 279-286.
ERR108489	rpoB_S450L	katG_S315T	Russia	Casali, N, et al. Nature genetics 46.3 (2014): 279-286.
ERR108491	rpoB_S450L	katG_S315T	Russia	Casali, N, et al. Nature genetics 46.3 (2014): 279-286.
ERR108494	rpoB_D435V	katG_S315T	Russia	Casali, N, et al. Nature genetics 46.3 (2014): 279-286.
ERR108495	rpoB_S450L	katG_S315T	Russia	Casali, N, et al. Nature genetics 46.3 (2014): 279-286.
ERR108498	rpoB_S450L	katG_S315T	Russia	Casali, N, et al. Nature genetics 46.3 (2014): 279-286.
ERR108499	rpoB_S450L	katG_S315T	Russia	Casali, N, et al. Nature genetics 46.3 (2014): 279-286.
ERR108500	rpoB_H445N,rpoB_L452P	katG_S315T,fabG1_promoter_C1673425T	Russia	Casali, N, et al. Nature genetics 46.3 (2014): 279-286.
ERR108502	rpoB_S450L	katG_S315T	Russia	Casali, N, et al. Nature genetics 46.3 (2014): 279-286.
ERR108503	rpoB_S450L	katG_S315T	Russia	Casali, N, et al. Nature genetics 46.3 (2014): 279-286.
ERR108507	rpoB_S450L	katG_S315T	Russia	Casali, N, et al. Nature genetics 46.3 (2014): 279-286.
ERR108510	rpoB_S450L	katG_S315T	Russia	Casali, N, et al. Nature genetics 46.3 (2014): 279-286.
ERR108511	rpoB_S450L	inhA_S94A,fabG1_promoter_C1673425T	Russia	Casali, N, et al. Nature genetics 46.3 (2014): 279-286.
ERR108512	rpoB_H445Y	kasA_G269S,katG_S315T,fabG1_promoter_C1673425T	Russia	Casali, N, et al. Nature genetics 46.3 (2014): 279-286.
ERR108514	rpoB_S450L	katG_S315T,fabG1_promoter_T1673432A	Russia	Casali, N, et al. Nature genetics 46.3 (2014): 279-286.
ERR108515	rpoB_S450L	katG_S315T	Russia	Casali, N, et al. Nature genetics 46.3 (2014): 279-286.
ERR117452	rpoB_D435V	kasA_G269S,katG_S315T,fabG1_promoter_C1673425T	Russia	Casali, N, et al. Nature genetics 46.3 (2014): 279-286.
ERR117453	rpoB_S450L	katG_S315T	Russia	Casali, N, et al. Nature genetics 46.3 (2014): 279-286.
ERR117454	rpoB_S450L	katG_S315T	Russia	Casali, N, et al. Nature genetics 46.3 (2014): 279-286.
ERR117456	rpoB_L452P	katG_S315T	Russia	Casali, N, et al. Nature genetics 46.3 (2014): 279-286.
ERR117459	rpoB_L430P	katG_S315T	Russia	Casali, N, et al. Nature genetics 46.3 (2014): 279-286.
ERR117460	rpoB_S450L	katG_S315T	Russia	Casali, N, et al. Nature genetics 46.3 (2014): 279-286.
ERR117461	rpoB_S450L	katG_S315T	Russia	Casali, N, et al. Nature genetics 46.3 (2014): 279-286.
ERR117462	rpoB_L430R,rpoB_D435Y	katG_S315T	Russia	Casali, N, et al. Nature genetics 46.3 (2014): 279-286.
ERR117463	rpoB_S450L	katG_S315T	Russia	Casali, N, et al. Nature genetics 46.3 (2014): 279-286.
ERR117464	rpoB_S450L	katG_S315T	Russia	Casali, N, et al. Nature genetics 46.3 (2014): 279-286.
ERR117466	rpoB_D435Y	kasA_G269S,katG_S315T,fabG1_promoter_T1673432A	Russia	Casali, N, et al. Nature genetics 46.3 (2014): 279-286.
ERR117468	rpoB_S450L	katG_S315T	Russia	Casali, N, et al. Nature genetics 46.3 (2014): 279-286.
ERR117470	rpoB_S450L	katG_S315T	Russia	Casali, N, et al. Nature genetics 46.3 (2014): 279-286.
ERR133800	rpoB_S450L	katG_S315T,katG_S315R,katG_S315T	Russia	Casali, N, et al. Nature genetics 46.3 (2014): 279-286.
ERR133805	rpoB_S450L	katG_S315T	Russia	Casali, N, et al. Nature genetics 46.3 (2014): 279-286.
ERR133806	rpoB_S450L	katG_S315T	Russia	Casali, N, et al. Nature genetics 46.3 (2014): 279-286.
ERR133812	rpoB_L452P	katG_S315T,fabG1_promoter_C1673425T	Russia	Casali, N, et al. Nature genetics 46.3 (2014): 279-286.
ERR133816	rpoB_S450L	katG_S315T	Russia	Casali, N, et al. Nature genetics 46.3 (2014): 279-286.
ERR133826	rpoB_S450L	katG_S315T	Russia	Casali, N, et al. Nature genetics 46.3 (2014): 279-286.
ERR133827	rpoB_S450L	katG_S315T	Russia	Casali, N, et al. Nature genetics 46.3 (2014): 279-286.
ERR133831	rpoB_S450L	katG_S315T	Russia	Casali, N, et al. Nature genetics 46.3 (2014): 279-286.
ERR133834	rpoB_S450L	katG_S315T	Russia	Casali, N, et al. Nature genetics 46.3 (2014): 279-286.
ERR133839	rpoB_S450L	katG_S315T	Russia	Casali, N, et al. Nature genetics 46.3 (2014): 279-286.
ERR133844	rpoB_S450L	katG_S315T	Russia	Casali, N, et al. Nature genetics 46.3 (2014): 279-286.
ERR133845	rpoB_S450L	katG_S315T	Russia	Casali, N, et al. Nature genetics 46.3 (2014): 279-286.
ERR133848	rpoB_S450L	katG_S315T	Russia	Casali, N, et al. Nature genetics 46.3 (2014): 279-286.
ERR133849	rpoB_S450L	katG_S315T	Russia	Casali, N, et al. Nature genetics 46.3 (2014): 279-286.
ERR133852	rpoB_S450L	fabG1_promoter_C1673425T	Russia	Casali, N, et al. Nature genetics 46.3 (2014): 279-286.
ERR133853	rpoB_S450L	katG_S315T	Russia	Casali, N, et al. Nature genetics 46.3 (2014): 279-286.
ERR133854	rpoB_S450L	katG_S315T	Russia	Casali, N, et al. Nature genetics 46.3 (2014): 279-286.
ERR133855	rpoB_S450L	katG_S315T	Russia	Casali, N, et al. Nature genetics 46.3 (2014): 279-286.

ERR133856	rpoB_L430P	katG_S315T	Russia	Casali, N, et al. Nature genetics 46.3 (2014): 279-286.
ERR133858	rpoB_S450L	katG_S315T	Russia	Casali, N, et al. Nature genetics 46.3 (2014): 279-286.
ERR133860	rpoB_S450L	katG_S315T	Russia	Casali, N, et al. Nature genetics 46.3 (2014): 279-286.
ERR133861	rpoB_S450L	katG_S315T	Russia	Casali, N, et al. Nature genetics 46.3 (2014): 279-286.
ERR133866	rpoB_S450L	katG_S315T	Russia	Casali, N, et al. Nature genetics 46.3 (2014): 279-286.
ERR133867	rpoB_L430R,rpoB_D435Y	katG_S315T	Russia	Casali, N, et al. Nature genetics 46.3 (2014): 279-286.
ERR133868	rpoB_S450L	katG_S315T	Russia	Casali, N, et al. Nature genetics 46.3 (2014): 279-286.
ERR133873	rpoB_L430P,rpoB_D435G	katG_S315T	Russia	Casali, N, et al. Nature genetics 46.3 (2014): 279-286.
ERR133878	rpoB_S450L	katG_S315T,katG_S315R,katG_S315T	Russia	Casali, N, et al. Nature genetics 46.3 (2014): 279-286.
ERR133879	rpoB_S450L	katG_S315T	Russia	Casali, N, et al. Nature genetics 46.3 (2014): 279-286.
ERR133880	rpoB_S450L	katG_S315T	Russia	Casali, N, et al. Nature genetics 46.3 (2014): 279-286.
ERR133884	rpoB_S450L	katG_S315T	Russia	Casali, N, et al. Nature genetics 46.3 (2014): 279-286.
ERR133885	rpoB_S450L	katG_S315T	Russia	Casali, N, et al. Nature genetics 46.3 (2014): 279-286.
ERR133886	rpoB_S450L	katG_S315T	Russia	Casali, N, et al. Nature genetics 46.3 (2014): 279-286.
ERR133887	rpoB_S450L	kasA_G269S,katG_S315T,fabG1_promoter_C1673425T	Russia	Casali, N, et al. Nature genetics 46.3 (2014): 279-286.
ERR133889	rpoB_S450L	katG_S315T	Russia	Casali, N, et al. Nature genetics 46.3 (2014): 279-286.
ERR133890	rpoB_S450L	katG_S315T	Russia	Casali, N, et al. Nature genetics 46.3 (2014): 279-286.
ERR133892	rpoB_S450L	katG_S315T	Russia	Casali, N, et al. Nature genetics 46.3 (2014): 279-286.
ERR133895	rpoB_S450L	katG_S315T	Russia	Casali, N, et al. Nature genetics 46.3 (2014): 279-286.
ERR133896	rpoB_S450L	katG_S315T	Russia	Casali, N, et al. Nature genetics 46.3 (2014): 279-286.
ERR133899	rpoB_S450L	katG_S315T	Russia	Casali, N, et al. Nature genetics 46.3 (2014): 279-286.
ERR133901	rpoB_S450L	katG_S315T	Russia	Casali, N, et al. Nature genetics 46.3 (2014): 279-286.
ERR133902	rpoB_S450L	katG_S315T	Russia	Casali, N, et al. Nature genetics 46.3 (2014): 279-286.
ERR133903	rpoB_D435V	kasA_G269S,katG_S315T,fabG1_promoter_C1673425T	Russia	Casali, N, et al. Nature genetics 46.3 (2014): 279-286.
ERR133906	rpoB_S450L	katG_S315T	Russia	Casali, N, et al. Nature genetics 46.3 (2014): 279-286.
ERR133907	rpoB_S450L	katG_S315T	Russia	Casali, N, et al. Nature genetics 46.3 (2014): 279-286.
ERR133908	rpoB_S450L	katG_S315T	Russia	Casali, N, et al. Nature genetics 46.3 (2014): 279-286.
ERR133910	rpoB_S450L	katG_S315T,fabG1_promoter_C1673425T	Russia	Casali, N, et al. Nature genetics 46.3 (2014): 279-286.
ERR133915	rpoB_S450L	katG_S315T	Russia	Casali, N, et al. Nature genetics 46.3 (2014): 279-286.
ERR133918	rpoB_S450L	katG_S315T	Russia	Casali, N, et al. Nature genetics 46.3 (2014): 279-286.
ERR133919	rpoB_S450L	katG_S315T	Russia	Casali, N, et al. Nature genetics 46.3 (2014): 279-286.
ERR133922	rpoB_S450L	kasA_G269S,katG_S315T,fabG1_promoter_C1673425T	Russia	Casali, N, et al. Nature genetics 46.3 (2014): 279-286.
ERR133927	rpoB_S450L	katG_S315T	Russia	Casali, N, et al. Nature genetics 46.3 (2014): 279-286.
ERR133930	rpoB_S450L	katG_S315T	Russia	Casali, N, et al. Nature genetics 46.3 (2014): 279-286.
ERR133931	rpoB_S450L	katG_S315T	Russia	Casali, N, et al. Nature genetics 46.3 (2014): 279-286.
ERR133933	rpoB_D435G	katG_S315T	Russia	Casali, N, et al. Nature genetics 46.3 (2014): 279-286.
ERR133935	rpoB_L430R,rpoB_D435Y	katG_S315T	Russia	Casali, N, et al. Nature genetics 46.3 (2014): 279-286.
ERR133936	rpoB_S450L	katG_S315T	Russia	Casali, N, et al. Nature genetics 46.3 (2014): 279-286.
ERR133937	rpoB_S450L	katG_S315T	Russia	Casali, N, et al. Nature genetics 46.3 (2014): 279-286.
ERR133942	rpoB_S450L	katG_S315T,katG_S315R,katG_S315T	Russia	Casali, N, et al. Nature genetics 46.3 (2014): 279-286.
ERR133944	rpoB_S450L	katG_S315T	Russia	Casali, N, et al. Nature genetics 46.3 (2014): 279-286.
ERR133945	rpoB_L430P	ahpC_promoter_G2726145A	Russia	Casali, N, et al. Nature genetics 46.3 (2014): 279-286.
ERR133951	rpoB_S450L	katG_S315T	Russia	Casali, N, et al. Nature genetics 46.3 (2014): 279-286.
ERR133953	rpoB_S450L	katG_S315T	Russia	Casali, N, et al. Nature genetics 46.3 (2014): 279-286.
ERR133954	rpoB_S450L	katG_S315T	Russia	Casali, N, et al. Nature genetics 46.3 (2014): 279-286.
ERR133957	rpoB_S450L	katG_S315T	Russia	Casali, N, et al. Nature genetics 46.3 (2014): 279-286.
ERR133959	rpoB_S450L	katG_S315T	Russia	Casali, N, et al. Nature genetics 46.3 (2014): 279-286.
ERR133966	rpoB_S450L	katG_S315T	Russia	Casali, N, et al. Nature genetics 46.3 (2014): 279-286.
ERR133970	rpoB_L430P	katG_S315T	Russia	Casali, N, et al. Nature genetics 46.3 (2014): 279-286.
ERR133981	rpoB_S450Q	ahpC_promoter_C2726112T	Russia	Casali, N, et al. Nature genetics 46.3 (2014): 279-286.
ERR133984	rpoB_S450L	katG_S315T	Russia	Casali, N, et al. Nature genetics 46.3 (2014): 279-286.
ERR137192	rpoB_S450L	katG_S315T	Russia	Casali, N, et al. Nature genetics 46.3 (2014): 279-286.
ERR137196	rpoB_H445D	katG_S315T	Russia	Casali, N, et al. Nature genetics 46.3 (2014): 279-286.
ERR137204	rpoB_S450L	katG_S315T	Russia	Casali, N, et al. Nature genetics 46.3 (2014): 279-286.
ERR137206	rpoB_H445N,rpoB_L452P	katG_S315T,fabG1_promoter_C1673425T	Russia	Casali, N, et al. Nature genetics 46.3 (2014): 279-286.

ERR137208	rpoB_S450L	katG_S315T	Russia	Casali, N, et al. Nature genetics 46.3 (2014): 279-286.
ERR137209	rpoB_S450L	kasA_G269S,katG_S315T,fabG1_promoter_C1673425T	Russia	Casali, N, et al. Nature genetics 46.3 (2014): 279-286.
ERR137210	rpoB_S450L	katG_S315T	Russia	Casali, N, et al. Nature genetics 46.3 (2014): 279-286.
ERR137211	rpoB_S450L	katG_S315T	Russia	Casali, N, et al. Nature genetics 46.3 (2014): 279-286.
ERR137217	rpoB_S450L	katG_S315T	Russia	Casali, N, et al. Nature genetics 46.3 (2014): 279-286.
ERR137218	rpoB_S450L	katG_S315T	Russia	Casali, N, et al. Nature genetics 46.3 (2014): 279-286.
ERR137219	rpoB_S450L	katG_S315T	Russia	Casali, N, et al. Nature genetics 46.3 (2014): 279-286.
ERR137220	rpoB_S450L	katG_S315T	Russia	Casali, N, et al. Nature genetics 46.3 (2014): 279-286.
ERR137222	rpoB_S450L	katG_S315T	Russia	Casali, N, et al. Nature genetics 46.3 (2014): 279-286.
ERR137223	rpoB_S450L	katG_S315T	Russia	Casali, N, et al. Nature genetics 46.3 (2014): 279-286.
ERR137225	rpoB_S450L	katG_S315T	Russia	Casali, N, et al. Nature genetics 46.3 (2014): 279-286.
ERR137226	rpoB_S450L	katG_S315T	Russia	Casali, N, et al. Nature genetics 46.3 (2014): 279-286.
ERR137229	rpoB_S450L	katG_S315T	Russia	Casali, N, et al. Nature genetics 46.3 (2014): 279-286.
ERR137230	rpoB_S450L	katG_S315T	Russia	Casali, N, et al. Nature genetics 46.3 (2014): 279-286.
ERR137231	rpoB_S450L	katG_S315T	Russia	Casali, N, et al. Nature genetics 46.3 (2014): 279-286.
ERR137234	rpoB_S450L	katG_S315T	Russia	Casali, N, et al. Nature genetics 46.3 (2014): 279-286.
ERR137236	rpoB_S450L	katG_S315T	Russia	Casali, N, et al. Nature genetics 46.3 (2014): 279-286.
ERR137237	rpoB_S450L	katG_S315T	Russia	Casali, N, et al. Nature genetics 46.3 (2014): 279-286.
ERR137243	rpoB_S450L	katG_S315T,fabG1_promoter_C1673425T	Russia	Casali, N, et al. Nature genetics 46.3 (2014): 279-286.
ERR137244	rpoB_S450L	katG_S315T	Russia	Casali, N, et al. Nature genetics 46.3 (2014): 279-286.
ERR137246	rpoB_S450L	katG_S315T	Russia	Casali, N, et al. Nature genetics 46.3 (2014): 279-286.
ERR137247	rpoB_S450L	katG_S315T	Russia	Casali, N, et al. Nature genetics 46.3 (2014): 279-286.
ERR137248	rpoB_S450L	katG_S315T	Russia	Casali, N, et al. Nature genetics 46.3 (2014): 279-286.
ERR137250	rpoB_H445R,rpoB_H445Y	katG_S315T	Russia	Casali, N, et al. Nature genetics 46.3 (2014): 279-286.
ERR137254	rpoB_S450L	katG_S315T	Russia	Casali, N, et al. Nature genetics 46.3 (2014): 279-286.
ERR137255	rpoB_S450L	katG_S315T	Russia	Casali, N, et al. Nature genetics 46.3 (2014): 279-286.
ERR137257	rpoB_S450L	katG_S315T	Russia	Casali, N, et al. Nature genetics 46.3 (2014): 279-286.
ERR137259	rpoB_S450L	katG_S315T	Russia	Casali, N, et al. Nature genetics 46.3 (2014): 279-286.
ERR137260	rpoB_S450L	katG_S315T	Russia	Casali, N, et al. Nature genetics 46.3 (2014): 279-286.
ERR137262	rpoB_S450L	katG_S315T	Russia	Casali, N, et al. Nature genetics 46.3 (2014): 279-286.
ERR137264	rpoB_S450L	katG_S315T	Russia	Casali, N, et al. Nature genetics 46.3 (2014): 279-286.
ERR137265	rpoB_S450L	katG_S315T	Russia	Casali, N, et al. Nature genetics 46.3 (2014): 279-286.
ERR137269	rpoB_S450L	katG_S315T	Russia	Casali, N, et al. Nature genetics 46.3 (2014): 279-286.
ERR137271	rpoB_S450L	katG_S315T	Russia	Casali, N, et al. Nature genetics 46.3 (2014): 279-286.
ERR137272	rpoB_S450L	katG_S315T	Russia	Casali, N, et al. Nature genetics 46.3 (2014): 279-286.
ERR137273	rpoB_S450L	katG_S315T,fabG1_promoter_T1673432A	Russia	Casali, N, et al. Nature genetics 46.3 (2014): 279-286.
ERR137274	rpoB_S450L	katG_S315T	Russia	Casali, N, et al. Nature genetics 46.3 (2014): 279-286.
ERR137275	rpoB_S450L	katG_S315T	Russia	Casali, N, et al. Nature genetics 46.3 (2014): 279-286.
ERR137277	rpoB_S450L	katG_S315T	Russia	Casali, N, et al. Nature genetics 46.3 (2014): 279-286.
ERR137281	rpoB_S450L	katG_S315T	Russia	Casali, N, et al. Nature genetics 46.3 (2014): 279-286.
ERR137282	rpoB_S450L	katG_S315T	Russia	Casali, N, et al. Nature genetics 46.3 (2014): 279-286.
ERR137283	rpoB_S450L	katG_S315T	Russia	Casali, N, et al. Nature genetics 46.3 (2014): 279-286.
ERR137284	rpoB_D435V	kasA_G269S,katG_S315T,fabG1_promoter_C1673425T	Russia	Casali, N, et al. Nature genetics 46.3 (2014): 279-286.
ERR144543	rpoB_S450L	katG_S315T	Russia	Casali, N, et al. Nature genetics 46.3 (2014): 279-286.
ERR144548	rpoB_S450L	katG_S315T	Russia	Casali, N, et al. Nature genetics 46.3 (2014): 279-286.
ERR144549	rpoB_S450L	katG_S315T	Russia	Casali, N, et al. Nature genetics 46.3 (2014): 279-286.
ERR144551	rpoB_S450L	katG_S315T	Russia	Casali, N, et al. Nature genetics 46.3 (2014): 279-286.
ERR144553	rpoB_S450L	katG_S315T	Russia	Casali, N, et al. Nature genetics 46.3 (2014): 279-286.
ERR144554	rpoB_S450L	katG_S315T	Russia	Casali, N, et al. Nature genetics 46.3 (2014): 279-286.
ERR144556	rpoB_S450L	katG_S315T	Russia	Casali, N, et al. Nature genetics 46.3 (2014): 279-286.
ERR144558	rpoB_L430P	katG_S315T	Russia	Casali, N, et al. Nature genetics 46.3 (2014): 279-286.
ERR144560	rpoB_S450L	katG_S315T	Russia	Casali, N, et al. Nature genetics 46.3 (2014): 279-286.
ERR144561	rpoB_S450L	katG_S315T	Russia	Casali, N, et al. Nature genetics 46.3 (2014): 279-286.
ERR144562	rpoB_S450L	katG_S315T	Russia	Casali, N, et al. Nature genetics 46.3 (2014): 279-286.
ERR144563	rpoB_S450L	katG_S315T,fabG1_promoter_C1673425T	Russia	Casali, N, et al. Nature genetics 46.3 (2014): 279-286.
ERR144566	rpoB_S450L	katG_S315T	Russia	Casali, N, et al. Nature genetics 46.3 (2014): 279-286.
ERR144567	rpoB_S450L	katG_S315T,katG_I335V	Russia	Casali, N, et al. Nature genetics 46.3 (2014): 279-286.
ERR144570	rpoB_S450L	katG_S315T	Russia	Casali, N, et al. Nature genetics 46.3 (2014): 279-286.

ERR144571	rpoB_S450L	katG_S315T	Russia	Casali, N, et al. Nature genetics 46.3 (2014): 279-286.
ERR144572	rpoB_H445L	katG_S315T	Russia	Casali, N, et al. Nature genetics 46.3 (2014): 279-286.
ERR144573	rpoB_S450L	katG_S315T	Russia	Casali, N, et al. Nature genetics 46.3 (2014): 279-286.
ERR144574	rpoB_S450L	katG_S315T,fabG1_promoter_T1673432C	Russia	Casali, N, et al. Nature genetics 46.3 (2014): 279-286.
ERR144575	rpoB_S450L	katG_S315T	Russia	Casali, N, et al. Nature genetics 46.3 (2014): 279-286.
ERR144581	rpoB_S450L	katG_S315T	Russia	Casali, N, et al. Nature genetics 46.3 (2014): 279-286.
ERR144584	rpoB_S450L	katG_S315T	Russia	Casali, N, et al. Nature genetics 46.3 (2014): 279-286.
ERR144589	rpoB_S450L	katG_S315T	Russia	Casali, N, et al. Nature genetics 46.3 (2014): 279-286.
ERR144590	rpoB_S450L	katG_S315T	Russia	Casali, N, et al. Nature genetics 46.3 (2014): 279-286.
ERR144593	rpoB_S450L	katG_S315T	Russia	Casali, N, et al. Nature genetics 46.3 (2014): 279-286.
ERR144595	rpoB_S450L	katG_S315T	Russia	Casali, N, et al. Nature genetics 46.3 (2014): 279-286.
ERR144596	rpoB_L430P	katG_S315T	Russia	Casali, N, et al. Nature genetics 46.3 (2014): 279-286.
ERR144597	rpoB_S450L	katG_S315T	Russia	Casali, N, et al. Nature genetics 46.3 (2014): 279-286.
ERR144600	rpoB_S450L	katG_S315T	Russia	Casali, N, et al. Nature genetics 46.3 (2014): 279-286.
ERR144601	rpoB_S450L	katG_S315T	Russia	Casali, N, et al. Nature genetics 46.3 (2014): 279-286.
ERR144607	rpoB_S450L	katG_S315T	Russia	Casali, N, et al. Nature genetics 46.3 (2014): 279-286.
ERR144608	rpoB_L430P	katG_S315T	Russia	Casali, N, et al. Nature genetics 46.3 (2014): 279-286.
ERR144611	rpoB_S450L	katG_S315T	Russia	Casali, N, et al. Nature genetics 46.3 (2014): 279-286.
ERR144615	rpoB_S450L	katG_S315T	Russia	Casali, N, et al. Nature genetics 46.3 (2014): 279-286.
ERR144617	rpoB_S450L	katG_S315T	Russia	Casali, N, et al. Nature genetics 46.3 (2014): 279-286.
ERR144619	rpoB_S450L	katG_S315T,fabG1_promoter_C1673425T	Russia	Casali, N, et al. Nature genetics 46.3 (2014): 279-286.
ERR144620	rpoB_S450L	katG_S315T	Russia	Casali, N, et al. Nature genetics 46.3 (2014): 279-286.
ERR144622	rpoB_H445L	katG_S315T,fabG1_promoter_C1673425T	Russia	Casali, N, et al. Nature genetics 46.3 (2014): 279-286.
ERR144623	rpoB_S450L	katG_S315T	Russia	Casali, N, et al. Nature genetics 46.3 (2014): 279-286.
ERR144624	rpoB_S450L	katG_S315T,fabG1_promoter_C1673425T	Russia	Casali, N, et al. Nature genetics 46.3 (2014): 279-286.
ERR144628	rpoB_S450L	katG_S315T	Russia	Casali, N, et al. Nature genetics 46.3 (2014): 279-286.
ERR144631	rpoB_S450L	fabG1_promoter_C1673425T	Russia	Casali, N, et al. Nature genetics 46.3 (2014): 279-286.
ERR144633	rpoB_S450L	katG_S315T	Russia	Casali, N, et al. Nature genetics 46.3 (2014): 279-286.
ERR144634	rpoB_S450L	katG_S315T,fabG1_promoter_C1673425T	Russia	Casali, N, et al. Nature genetics 46.3 (2014): 279-286.
ERR144635	rpoB_S450L	katG_S315T	Russia	Casali, N, et al. Nature genetics 46.3 (2014): 279-286.
ERR158570	rpoB_D435V	katG_S315T	Russia	Casali, N, et al. Nature genetics 46.3 (2014): 279-286.
ERR158576	rpoB_S450L	katG_S315T	Russia	Casali, N, et al. Nature genetics 46.3 (2014): 279-286.
ERR158578	rpoB_S450L	katG_S315T,fabG1_promoter_C1673425T	Russia	Casali, N, et al. Nature genetics 46.3 (2014): 279-286.
ERR158579	rpoB_D435V	kasA_G269S,katG_S315T,fabG1_promoter_C1673425T	Russia	Casali, N, et al. Nature genetics 46.3 (2014): 279-286.
ERR158580	rpoB_S450L	katG_S315T	Russia	Casali, N, et al. Nature genetics 46.3 (2014): 279-286.
ERR158581	rpoB_S450L	katG_S315T	Russia	Casali, N, et al. Nature genetics 46.3 (2014): 279-286.
ERR158585	rpoB_S450L	katG_S315T	Russia	Casali, N, et al. Nature genetics 46.3 (2014): 279-286.
ERR158586	rpoB_S450L	katG_S315T	Russia	Casali, N, et al. Nature genetics 46.3 (2014): 279-286.
ERR158587	rpoB_S450L	katG_S315T	Russia	Casali, N, et al. Nature genetics 46.3 (2014): 279-286.
ERR158588	rpoB_S450L	katG_S315T	Russia	Casali, N, et al. Nature genetics 46.3 (2014): 279-286.
ERR158592	rpoB_S450L	katG_S315T	Russia	Casali, N, et al. Nature genetics 46.3 (2014): 279-286.
ERR158595	rpoB_S450W	kasA_G269S,katG_S315T,fabG1_promoter_G1673423T	Russia	Casali, N, et al. Nature genetics 46.3 (2014): 279-286.
ERR158596	rpoB_S450L	katG_S315T	Russia	Casali, N, et al. Nature genetics 46.3 (2014): 279-286.
ERR158597	rpoB_S450L	katG_S315T	Russia	Casali, N, et al. Nature genetics 46.3 (2014): 279-286.
ERR158598	rpoB_S450L	katG_S315T	Russia	Casali, N, et al. Nature genetics 46.3 (2014): 279-286.
ERR158602	rpoB_S450L	katG_S315T	Russia	Casali, N, et al. Nature genetics 46.3 (2014): 279-286.
ERR158606	rpoB_H445Y	katG_S315T	Russia	Casali, N, et al. Nature genetics 46.3 (2014): 279-286.
ERR158607	rpoB_S450L	katG_S315T,fabG1_promoter_C1673425T	Russia	Casali, N, et al. Nature genetics 46.3 (2014): 279-286.
ERR158608	rpoB_S450L	katG_S315T	Russia	Casali, N, et al. Nature genetics 46.3 (2014): 279-286.
ERR158609	rpoB_S450L	katG_S315T	Russia	Casali, N, et al. Nature genetics 46.3 (2014): 279-286.
ERR158611	rpoB_S450L	katG_S315T	Russia	Casali, N, et al. Nature genetics 46.3 (2014): 279-286.
ERR158613	rpoB_S450L	katG_S315T	Russia	Casali, N, et al. Nature genetics 46.3 (2014): 279-286.
ERR158616	rpoB_S450L	katG_S315T,katG_I335V	Russia	Casali, N, et al. Nature genetics 46.3 (2014): 279-286.
ERR227975	rpoB_S450L	katG_S315T	Russia	Casali, N, et al. Nature genetics 46.3 (2014): 279-286.

ERR227976	rpoB_S450L	katG_S315T	Russia	Casali, N, et al. Nature genetics 46.3 (2014): 279-286.
ERR227977	rpoB_S450L	katG_S315T	Russia	Casali, N, et al. Nature genetics 46.3 (2014): 279-286.
ERR227978	rpoB_S450L	katG_S315T	Russia	Casali, N, et al. Nature genetics 46.3 (2014): 279-286.
ERR227981	rpoB_S450L	katG_S315T	Russia	Casali, N, et al. Nature genetics 46.3 (2014): 279-286.
ERR227982	rpoB_S450L	katG_S315T	Russia	Casali, N, et al. Nature genetics 46.3 (2014): 279-286.
ERR227983	rpoB_S450L	inhA_S94A,fabG1_promoter_C1673425T	Russia	Casali, N, et al. Nature genetics 46.3 (2014): 279-286.
ERR227985	rpoB_S450L	katG_S315T,fabG1_promoter_C1673425T	Russia	Casali, N, et al. Nature genetics 46.3 (2014): 279-286.
ERR227986	rpoB_S450L	katG_S315T,fabG1_promoter_C1673425T	Russia	Casali, N, et al. Nature genetics 46.3 (2014): 279-286.
ERR227987	rpoB_S450L	katG_S315T	Russia	Casali, N, et al. Nature genetics 46.3 (2014): 279-286.
ERR227995	rpoB_S450L	katG_S315T	Russia	Casali, N, et al. Nature genetics 46.3 (2014): 279-286.
ERR228001	rpoB_S450L	katG_S315T,fabG1_promoter_C1673425T	Russia	Casali, N, et al. Nature genetics 46.3 (2014): 279-286.
ERR228003	rpoB_S450L	katG_S315T	Russia	Casali, N, et al. Nature genetics 46.3 (2014): 279-286.
ERR228006	rpoB_S450L	katG_S315T	Russia	Casali, N, et al. Nature genetics 46.3 (2014): 279-286.
ERR228009	rpoB_S450L	kasA_G269S,katG_S315T	Russia	Casali, N, et al. Nature genetics 46.3 (2014): 279-286.
ERR228012	rpoB_S450L	katG_S315T	Russia	Casali, N, et al. Nature genetics 46.3 (2014): 279-286.
ERR228013	rpoB_S450L	katG_S315T	Russia	Casali, N, et al. Nature genetics 46.3 (2014): 279-286.
ERR228017	rpoB_S450L	katG_S315T	Russia	Casali, N, et al. Nature genetics 46.3 (2014): 279-286.
ERR228019	rpoB_S450L	katG_S315T	Russia	Casali, N, et al. Nature genetics 46.3 (2014): 279-286.
ERR228020	rpoB_S450L	katG_S315T	Russia	Casali, N, et al. Nature genetics 46.3 (2014): 279-286.
ERR228024	rpoB_S450L	katG_S315T	Russia	Casali, N, et al. Nature genetics 46.3 (2014): 279-286.
ERR228025	rpoB_S450L	katG_S315N	Russia	Casali, N, et al. Nature genetics 46.3 (2014): 279-286.
ERR228028	rpoB_S450L	katG_S315T	Russia	Casali, N, et al. Nature genetics 46.3 (2014): 279-286.
ERR228029	rpoB_S450L	katG_S315T	Russia	Casali, N, et al. Nature genetics 46.3 (2014): 279-286.
ERR228032	rpoB_S450L	fabG1_promoter_C1673425T	Russia	Casali, N, et al. Nature genetics 46.3 (2014): 279-286.
ERR228036	rpoB_H445D	kasA_G269S,katG_S315T,fabG1_promoter_C1673425T	Russia	Casali, N, et al. Nature genetics 46.3 (2014): 279-286.
ERR228037	rpoB_S450L	katG_S315T	Russia	Casali, N, et al. Nature genetics 46.3 (2014): 279-286.
ERR228039	rpoB_S450L	katG_S315T	Russia	Casali, N, et al. Nature genetics 46.3 (2014): 279-286.
ERR228042	rpoB_S450L	katG_S315T	Russia	Casali, N, et al. Nature genetics 46.3 (2014): 279-286.
ERR228043	rpoB_S450L	katG_S315T	Russia	Casali, N, et al. Nature genetics 46.3 (2014): 279-286.
ERR228046	rpoB_S450L	katG_S315T	Russia	Casali, N, et al. Nature genetics 46.3 (2014): 279-286.
ERR228049	rpoB_S450L	katG_S315T	Russia	Casali, N, et al. Nature genetics 46.3 (2014): 279-286.
ERR228050	rpoB_S450L	katG_S315T	Russia	Casali, N, et al. Nature genetics 46.3 (2014): 279-286.
ERR228051	rpoB_S450L	inhA_S94A,katG_S315T	Russia	Casali, N, et al. Nature genetics 46.3 (2014): 279-286.
ERR228054	rpoB_S450L	katG_S315T	Russia	Casali, N, et al. Nature genetics 46.3 (2014): 279-286.
ERR228056	rpoB_S450L	katG_S315T	Russia	Casali, N, et al. Nature genetics 46.3 (2014): 279-286.
ERR228059	rpoB_S450L	katG_S315T	Russia	Casali, N, et al. Nature genetics 46.3 (2014): 279-286.
ERR228061	rpoB_S450L	katG_S315T	Russia	Casali, N, et al. Nature genetics 46.3 (2014): 279-286.
ERR228062	rpoB_S450L	katG_S315T,katG_I335V	Russia	Casali, N, et al. Nature genetics 46.3 (2014): 279-286.
ERR228064	rpoB_S450L	katG_S315T	Russia	Casali, N, et al. Nature genetics 46.3 (2014): 279-286.
ERR229915	rpoB_S450L	katG_S315T	Russia	Casali, N, et al. Nature genetics 46.3 (2014): 279-286.
ERR229916	rpoB_S450L	katG_S315T	Russia	Casali, N, et al. Nature genetics 46.3 (2014): 279-286.
ERR229918	rpoB_S450L	katG_S315T	Russia	Casali, N, et al. Nature genetics 46.3 (2014): 279-286.
ERR229921	rpoB_S450L	katG_S315T	Russia	Casali, N, et al. Nature genetics 46.3 (2014): 279-286.
ERR229922	rpoB_S450L	katG_S315T	Russia	Casali, N, et al. Nature genetics 46.3 (2014): 279-286.
ERR229923	rpoB_S450L	katG_S315T	Russia	Casali, N, et al. Nature genetics 46.3 (2014): 279-286.
ERR229924	rpoB_S450L	katG_S315T	Russia	Casali, N, et al. Nature genetics 46.3 (2014): 279-286.
ERR229926	rpoB_S450L	katG_S315T	Russia	Casali, N, et al. Nature genetics 46.3 (2014): 279-286.
ERR229927	rpoB_S450L	kasA_G269S,katG_S315T	Russia	Casali, N, et al. Nature genetics 46.3 (2014): 279-286.
ERR229929	rpoB_S450L	katG_S315T	Russia	Casali, N, et al. Nature genetics 46.3 (2014): 279-286.
ERR229930	rpoB_S450L	katG_S315T	Russia	Casali, N, et al. Nature genetics 46.3 (2014): 279-286.
ERR229931	rpoB_S450L	katG_S315T	Russia	Casali, N, et al. Nature genetics 46.3 (2014): 279-286.
ERR229932	rpoB_S450L	katG_S315T	Russia	Casali, N, et al. Nature genetics 46.3 (2014): 279-286.
ERR229933	rpoB_S450L	katG_S315T	Russia	Casali, N, et al. Nature genetics 46.3 (2014): 279-286.
ERR229936	rpoB_S450L	katG_S315T	Russia	Casali, N, et al. Nature genetics 46.3 (2014): 279-286.
ERR229940	rpoB_H445R	katG_S315T	Russia	Casali, N, et al. Nature genetics 46.3 (2014): 279-286.
ERR229946	rpoB_S450L	katG_S315T	Russia	Casali, N, et al. Nature genetics 46.3 (2014): 279-286.

ERR229947	rpoB_L430R,rpoB_D435Y	katG_S315T	Russia	Casali, N, et al. Nature genetics 46.3 (2014): 279-286.
ERR229949	rpoB_S450L	katG_S315T	Russia	Casali, N, et al. Nature genetics 46.3 (2014): 279-286.
ERR229951	rpoB_S450L	katG_S315T	Russia	Casali, N, et al. Nature genetics 46.3 (2014): 279-286.
ERR229953	rpoB_S450L	katG_S315T	Russia	Casali, N, et al. Nature genetics 46.3 (2014): 279-286.
ERR229955	rpoB_S450L	katG_S315T	Russia	Casali, N, et al. Nature genetics 46.3 (2014): 279-286.
ERR229960	rpoB_S450L	katG_S315T	Russia	Casali, N, et al. Nature genetics 46.3 (2014): 279-286.
ERR229961	rpoB_D435Y	katG_S315T	Russia	Casali, N, et al. Nature genetics 46.3 (2014): 279-286.
ERR229962	rpoB_S450L	katG_S315T	Russia	Casali, N, et al. Nature genetics 46.3 (2014): 279-286.
ERR229964	rpoB_D435G	katG_S315T	Russia	Casali, N, et al. Nature genetics 46.3 (2014): 279-286.
ERR229965	rpoB_S450L	inhA_S94A,fabG1_promoter_C1673425T	Russia	Casali, N, et al. Nature genetics 46.3 (2014): 279-286.
ERR229966	rpoB_S450L	katG_S315T	Russia	Casali, N, et al. Nature genetics 46.3 (2014): 279-286.
ERR229968	rpoB_S450L	katG_S315T	Russia	Casali, N, et al. Nature genetics 46.3 (2014): 279-286.
ERR229969	rpoB_S450L	katG_S315T	Russia	Casali, N, et al. Nature genetics 46.3 (2014): 279-286.
ERR229971	rpoB_S450L	katG_S315T	Russia	Casali, N, et al. Nature genetics 46.3 (2014): 279-286.
ERR229974	rpoB_S450L	katG_S315T	Russia	Casali, N, et al. Nature genetics 46.3 (2014): 279-286.
ERR229976	rpoB_S450L	katG_S315T	Russia	Casali, N, et al. Nature genetics 46.3 (2014): 279-286.
ERR229978	rpoB_S450L	katG_S315T	Russia	Casali, N, et al. Nature genetics 46.3 (2014): 279-286.
ERR229980	rpoB_S450L	katG_S315T	Russia	Casali, N, et al. Nature genetics 46.3 (2014): 279-286.
ERR229982	rpoB_S450L	katG_S315T	Russia	Casali, N, et al. Nature genetics 46.3 (2014): 279-286.
ERR229984	rpoB_S450L	katG_S315T	Russia	Casali, N, et al. Nature genetics 46.3 (2014): 279-286.
ERR229988	rpoB_S450L	katG_S315T	Russia	Casali, N, et al. Nature genetics 46.3 (2014): 279-286.
ERR229993	rpoB_S450L	katG_S315T	Russia	Casali, N, et al. Nature genetics 46.3 (2014): 279-286.
ERR229994	rpoB_S450L	katG_S315T	Russia	Casali, N, et al. Nature genetics 46.3 (2014): 279-286.
ERR229995	rpoB_S450L	katG_S315T	Russia	Casali, N, et al. Nature genetics 46.3 (2014): 279-286.
ERR229997	rpoB_L430R,rpoB_D435Y	katG_S315T	Russia	Casali, N, et al. Nature genetics 46.3 (2014): 279-286.
ERR229998	rpoB_S450L	katG_S315T	Russia	Casali, N, et al. Nature genetics 46.3 (2014): 279-286.
ERR230000	rpoB_S450L	katG_S315T	Russia	Casali, N, et al. Nature genetics 46.3 (2014): 279-286.
ERR230003	rpoB_H445L	katG_S315T	Russia	Casali, N, et al. Nature genetics 46.3 (2014): 279-286.
ERR230005	rpoB_S450L	katG_S315T	Russia	Casali, N, et al. Nature genetics 46.3 (2014): 279-286.
ERR230008	rpoB_S450L	katG_S315T	Russia	Casali, N, et al. Nature genetics 46.3 (2014): 279-286.
ERR230009	rpoB_S450L	katG_S315T	Russia	Casali, N, et al. Nature genetics 46.3 (2014): 279-286.
ERR234556	rpoB_H445N,rpoB_L452P	katG_S315T,fabG1_promoter_C1673425T	Russia	Casali, N, et al. Nature genetics 46.3 (2014): 279-286.
ERR234560	rpoB_S450L	katG_S315T	Russia	Casali, N, et al. Nature genetics 46.3 (2014): 279-286.
ERR234562	rpoB_V170F	katG_S315T	Russia	Casali, N, et al. Nature genetics 46.3 (2014): 279-286.
ERR234563	rpoB_S450L	katG_S315T	Russia	Casali, N, et al. Nature genetics 46.3 (2014): 279-286.
ERR234565	rpoB_S450L	katG_S315T	Russia	Casali, N, et al. Nature genetics 46.3 (2014): 279-286.
ERR234568	rpoB_S450L	katG_S315T	Russia	Casali, N, et al. Nature genetics 46.3 (2014): 279-286.
ERR234570	rpoB_S450L	katG_S315T	Russia	Casali, N, et al. Nature genetics 46.3 (2014): 279-286.
ERR234573	rpoB_S450L	katG_S315T	Russia	Casali, N, et al. Nature genetics 46.3 (2014): 279-286.
ERR234574	rpoB_S450L	katG_S315T	Russia	Casali, N, et al. Nature genetics 46.3 (2014): 279-286.
ERR234576	rpoB_S450L	katG_S315T	Russia	Casali, N, et al. Nature genetics 46.3 (2014): 279-286.
ERR234584	rpoB_S450L	katG_S315T	Russia	Casali, N, et al. Nature genetics 46.3 (2014): 279-286.
ERR234589	rpoB_S450L	katG_S315T	Russia	Casali, N, et al. Nature genetics 46.3 (2014): 279-286.
ERR234593	rpoB_S450L	katG_S315T	Russia	Casali, N, et al. Nature genetics 46.3 (2014): 279-286.
ERR234594	rpoB_H445Y	katG_S315T,fabG1_promoter_T1673432G	Russia	Casali, N, et al. Nature genetics 46.3 (2014): 279-286.
ERR234596	rpoB_S450L	katG_S315T	Russia	Casali, N, et al. Nature genetics 46.3 (2014): 279-286.
ERR234597	rpoB_S450L	katG_S315T	Russia	Casali, N, et al. Nature genetics 46.3 (2014): 279-286.
ERR234598	rpoB_S450L	katG_S315T	Russia	Casali, N, et al. Nature genetics 46.3 (2014): 279-286.
ERR234600	rpoB_S450L	katG_S315T	Russia	Casali, N, et al. Nature genetics 46.3 (2014): 279-286.
ERR234602	rpoB_S450L	katG_S315T	Russia	Casali, N, et al. Nature genetics 46.3 (2014): 279-286.
ERR234606	rpoB_S450L	katG_S315T	Russia	Casali, N, et al. Nature genetics 46.3 (2014): 279-286.
ERR234607	rpoB_S450L	katG_S315T	Russia	Casali, N, et al. Nature genetics 46.3 (2014): 279-286.
ERR234608	rpoB_S450L	katG_S315T	Russia	Casali, N, et al. Nature genetics 46.3 (2014): 279-286.
ERR234609	rpoB_S450L	katG_S315T	Russia	Casali, N, et al. Nature genetics 46.3 (2014): 279-286.
ERR234610	rpoB_S450L	katG_S315T	Russia	Casali, N, et al. Nature genetics 46.3 (2014): 279-286.
ERR234612	rpoB_H445L	katG_S315T,fabG1_promoter_C1673425T	Russia	Casali, N, et al. Nature genetics 46.3 (2014): 279-286.
ERR234615	rpoB_S450L	katG_S315T	Russia	Casali, N, et al. Nature genetics 46.3 (2014): 279-286.
ERR234618	rpoB_S450L	katG_S315T	Russia	Casali, N, et al. Nature genetics 46.3 (2014): 279-286.
ERR234621	rpoB_S450L	katG_S315T	Russia	Casali, N, et al. Nature genetics 46.3 (2014): 279-286.
ERR234623	rpoB_S450L	katG_S315T	Russia	Casali, N, et al. Nature genetics 46.3 (2014): 279-286.

ERR234627	rpoB_H445Y	katG_S315T,fabG1_promoter_T1673432G	Russia	Casali, N, et al. Nature genetics 46.3 (2014): 279-286.
ERR234628	rpoB_S450L	katG_S315T	Russia	Casali, N, et al. Nature genetics 46.3 (2014): 279-286.
ERR234630	rpoB_S450L	katG_S315T	Russia	Casali, N, et al. Nature genetics 46.3 (2014): 279-286.
ERR234631	rpoB_S428R,rpoB_H445Y	katG_S315T	Russia	Casali, N, et al. Nature genetics 46.3 (2014): 279-286.
ERR234632	rpoB_S450L	katG_S315T	Russia	Casali, N, et al. Nature genetics 46.3 (2014): 279-286.
ERR234633	rpoB_S450L	katG_S315T	Russia	Casali, N, et al. Nature genetics 46.3 (2014): 279-286.
ERR234637	rpoB_S450L	katG_S315T	Russia	Casali, N, et al. Nature genetics 46.3 (2014): 279-286.
ERR234639	rpoB_S450L	katG_S315T	Russia	Casali, N, et al. Nature genetics 46.3 (2014): 279-286.
ERR234641	rpoB_S450L	katG_S315T	Russia	Casali, N, et al. Nature genetics 46.3 (2014): 279-286.
ERR234643	rpoB_S450L	katG_S315T	Russia	Casali, N, et al. Nature genetics 46.3 (2014): 279-286.
ERR234644	rpoB_S450L	katG_S315T	Russia	Casali, N, et al. Nature genetics 46.3 (2014): 279-286.
ERR234646	rpoB_S450L	katG_S315T	Russia	Casali, N, et al. Nature genetics 46.3 (2014): 279-286.
ERR234648	rpoB_S450L	katG_S315T,fabG1_promoter_C1673425T	Russia	Casali, N, et al. Nature genetics 46.3 (2014): 279-286.
ERR234649	rpoB_S450L	katG_S315T	Russia	Casali, N, et al. Nature genetics 46.3 (2014): 279-286.
ERR234650	rpoB_S450L	katG_S315T	Russia	Casali, N, et al. Nature genetics 46.3 (2014): 279-286.
ERR234651	rpoB_L452P	katG_S315T	Russia	Casali, N, et al. Nature genetics 46.3 (2014): 279-286.
ERR234653	rpoB_S450L	katG_S315T	Russia	Casali, N, et al. Nature genetics 46.3 (2014): 279-286.
ERR234654	rpoB_S450L	katG_S315T	Russia	Casali, N, et al. Nature genetics 46.3 (2014): 279-286.
ERR234656	rpoB_S450L	katG_S315T	Russia	Casali, N, et al. Nature genetics 46.3 (2014): 279-286.
ERR234657	rpoB_S450L	fabG1_promoter_C1673425T	Russia	Casali, N, et al. Nature genetics 46.3 (2014): 279-286.
ERR234658	rpoB_S450L	katG_S315T,katG_I335V	Russia	Casali, N, et al. Nature genetics 46.3 (2014): 279-286.
ERR234660	rpoB_S450L	katG_S315T	Russia	Casali, N, et al. Nature genetics 46.3 (2014): 279-286.
ERR234661	rpoB_S450L	katG_S315T	Russia	Casali, N, et al. Nature genetics 46.3 (2014): 279-286.
ERR234666	rpoB_S450L	katG_S315T	Russia	Casali, N, et al. Nature genetics 46.3 (2014): 279-286.
ERR234668	rpoB_S450L	katG_S315T	Russia	Casali, N, et al. Nature genetics 46.3 (2014): 279-286.
ERR234669	rpoB_S450L	katG_S315T,fabG1_promoter_C1673425T	Russia	Casali, N, et al. Nature genetics 46.3 (2014): 279-286.
ERR234670	rpoB_S450L	katG_S315T	Russia	Casali, N, et al. Nature genetics 46.3 (2014): 279-286.
ERR234671	rpoB_S450L	katG_S315T	Russia	Casali, N, et al. Nature genetics 46.3 (2014): 279-286.
ERR234672	rpoB_S450L	katG_S315T	Russia	Casali, N, et al. Nature genetics 46.3 (2014): 279-286.
ERR234688	rpoB_L430P	katG_S315T	Russia	Casali, N, et al. Nature genetics 46.3 (2014): 279-286.
ERR234692	rpoB_H445L	katG_S315T	Russia	Casali, N, et al. Nature genetics 46.3 (2014): 279-286.
ERR234698	rpoB_S450L	katG_S315T	Russia	Casali, N, et al. Nature genetics 46.3 (2014): 279-286.
ERR403214	rpoB_S450L	katG_S315T	Russia	Casali, N, et al. Nature genetics 46.3 (2014): 279-286.
ERR403218	rpoB_S450L	katG_S315T	Russia	Casali, N, et al. Nature genetics 46.3 (2014): 279-286.
ERR403219	rpoB_S450L	katG_S315T	Russia	Casali, N, et al. Nature genetics 46.3 (2014): 279-286.
ERR403221	rpoB_L452P	katG_S315T	Russia	Casali, N, et al. Nature genetics 46.3 (2014): 279-286.
ERR403222	rpoB_H445Y	katG_S315T	Russia	Casali, N, et al. Nature genetics 46.3 (2014): 279-286.
ERR403224	rpoB_D435Y	katG_S315T	Russia	Casali, N, et al. Nature genetics 46.3 (2014): 279-286.
ERR403227	rpoB_S450L	katG_S315T	Russia	Casali, N, et al. Nature genetics 46.3 (2014): 279-286.
ERR403228	rpoB_S450L	katG_S315T	Russia	Casali, N, et al. Nature genetics 46.3 (2014): 279-286.
ERR403229	rpoB_S450L	katG_S315T	Russia	Casali, N, et al. Nature genetics 46.3 (2014): 279-286.
ERR403230	rpoB_S450L	katG_S315T	Russia	Casali, N, et al. Nature genetics 46.3 (2014): 279-286.
ERR403236	rpoB_S450L	inhA_S94A,katG_S315T	Russia	Casali, N, et al. Nature genetics 46.3 (2014): 279-286.
ERR403240	rpoB_S450L	katG_S315T,fabG1_promoter_C1673425T	Russia	Casali, N, et al. Nature genetics 46.3 (2014): 279-286.
ERR403242	rpoB_S450L	katG_S315T	Russia	Casali, N, et al. Nature genetics 46.3 (2014): 279-286.
ERR403243	rpoB_D435Y	katG_S315T	Russia	Casali, N, et al. Nature genetics 46.3 (2014): 279-286.
ERR403244	rpoB_S450L	katG_S315T	Russia	Casali, N, et al. Nature genetics 46.3 (2014): 279-286.
ERR403245	rpoB_H445N	katG_S315T	Russia	Casali, N, et al. Nature genetics 46.3 (2014): 279-286.
ERR403247	rpoB_S450L	katG_S315T	Russia	Casali, N, et al. Nature genetics 46.3 (2014): 279-286.
ERR403250	rpoB_S450L	katG_S315T	Russia	Casali, N, et al. Nature genetics 46.3 (2014): 279-286.
ERR403259	rpoB_S450L	katG_S315T,fabG1_promoter_C1673425T	Russia	Casali, N, et al. Nature genetics 46.3 (2014): 279-286.
ERR403266	rpoB_S450L	katG_S315T	Russia	Casali, N, et al. Nature genetics 46.3 (2014): 279-286.
ERR403268	rpoB_S450L	katG_S315T	Russia	Casali, N, et al. Nature genetics 46.3 (2014): 279-286.
ERR403271	rpoB_S450L	katG_S315T	Russia	Casali, N, et al. Nature genetics 46.3 (2014): 279-286.
ERR403273	rpoB_S450L	katG_S315T	Russia	Casali, N, et al. Nature genetics 46.3 (2014): 279-286.
ERR403276	rpoB_S450L	katG_S315T	Russia	Casali, N, et al. Nature genetics 46.3 (2014): 279-286.
ERR403277	rpoB_S450L	katG_S315T	Russia	Casali, N, et al. Nature genetics 46.3 (2014): 279-286.
ERR403279	rpoB_S450L	katG_S315T	Russia	Casali, N, et al. Nature genetics 46.3 (2014): 279-286.

ERR403282	rpoB_S450L	katG_S315T	Russia	Casali, N, et al. Nature genetics 46.3 (2014): 279-286.
ERR403284	rpoB_S450L	katG_S315T	Russia	Casali, N, et al. Nature genetics 46.3 (2014): 279-286.
ERR403286	rpoB_S450L	katG_S315T	Russia	Casali, N, et al. Nature genetics 46.3 (2014): 279-286.
ERR403289	rpoB_S450L	katG_S315T	Russia	Casali, N, et al. Nature genetics 46.3 (2014): 279-286.
ERR403290	rpoB_S450L	katG_S315T	Russia	Casali, N, et al. Nature genetics 46.3 (2014): 279-286.
ERR403295	rpoB_S450L	katG_S315T	Russia	Casali, N, et al. Nature genetics 46.3 (2014): 279-286.
ERR403296	rpoB_S450L	katG_S315T	Russia	Casali, N, et al. Nature genetics 46.3 (2014): 279-286.
ERR403297	rpoB_S450L	katG_S315T	Russia	Casali, N, et al. Nature genetics 46.3 (2014): 279-286.
ERR403301	rpoB_S450L	katG_S315T,fabG1_promoter_C1673425T	Russia	Casali, N, et al. Nature genetics 46.3 (2014): 279-286.
ERR403302	rpoB_S450L	katG_S315T,fabG1_promoter_T1673432A	Russia	Casali, N, et al. Nature genetics 46.3 (2014): 279-286.
ERR403303	rpoB_S450L	katG_S315T	Russia	Casali, N, et al. Nature genetics 46.3 (2014): 279-286.
ERR403305	rpoB_S450L	katG_S315T	Russia	Casali, N, et al. Nature genetics 46.3 (2014): 279-286.
ERR403306	rpoB_S450L	inhA_I194T,katG_S315T	Russia	Casali, N, et al. Nature genetics 46.3 (2014): 279-286.
ERR403307	rpoB_S450L	katG_S315T	Russia	Casali, N, et al. Nature genetics 46.3 (2014): 279-286.
ERR403308	rpoB_D435V	kasA_G269S,katG_S315T	Russia	Casali, N, et al. Nature genetics 46.3 (2014): 279-286.
ERR403313	rpoB_S450L	katG_S315T	Russia	Casali, N, et al. Nature genetics 46.3 (2014): 279-286.
ERR403316	rpoB_S450L	katG_S315T	Russia	Casali, N, et al. Nature genetics 46.3 (2014): 279-286.
ERR403318	rpoB_S450L	katG_S315T	Russia	Casali, N, et al. Nature genetics 46.3 (2014): 279-286.
ERR403324	rpoB_S450L	katG_S315T	Russia	Casali, N, et al. Nature genetics 46.3 (2014): 279-286.
SRR1011449	rpoB_S450L	inhA_I194T,fabG1_promoter_C1673425T	South Africa	Cohen, K.A., et al. PLoS medicine 12.9 (2015): e1001880.
SRR1011450	rpoB_S450L	katG_S315T,fabG1_promoter_C1673425T	South Africa	Cohen, K.A., et al. PLoS medicine 12.9 (2015): e1001880.
SRR1011451	rpoB_S450L	katG_S315T,fabG1_promoter_C1673425T	South Africa	Cohen, K.A., et al. PLoS medicine 12.9 (2015): e1001880.
SRR1011452	rpoB_D435G,rpoB_L452P	kasA_G269S,katG_S315T,fabG1_promoter_T1673432A	South Africa	Cohen, K.A., et al. PLoS medicine 12.9 (2015): e1001880.
SRR1011453	rpoB_D435G,rpoB_L452P	kasA_G269S,katG_S315T,fabG1_promoter_T1673432A	South Africa	Cohen, K.A., et al. PLoS medicine 12.9 (2015): e1001880.
SRR1011454	rpoB_D435G,rpoB_L452P	katG_S315T,fabG1_promoter_T1673432A	South Africa	Cohen, K.A., et al. PLoS medicine 12.9 (2015): e1001880.
SRR1011455	rpoB_D435G,rpoB_L452P	katG_S315T,fabG1_promoter_T1673432A	South Africa	Cohen, K.A., et al. PLoS medicine 12.9 (2015): e1001880.
SRR1011456	rpoB_D435G,rpoB_L452P	katG_S315T,fabG1_promoter_T1673432A	South Africa	Cohen, K.A., et al. PLoS medicine 12.9 (2015): e1001880.
SRR1011457	rpoB_D435G,rpoB_L452P	kasA_G269S,katG_S315T,fabG1_promoter_T1673432A	South Africa	Cohen, K.A., et al. PLoS medicine 12.9 (2015): e1001880.
SRR1011459	rpoB_S450L	katG_S315T	South Africa	Cohen, K.A., et al. PLoS medicine 12.9 (2015): e1001880.
SRR1011460	rpoB_S450L	katG_S315T	South Africa	Cohen, K.A., et al. PLoS medicine 12.9 (2015): e1001880.
SRR1011461	rpoB_S450L	kasA_G269S,katG_S315T,ahpC_promoter_C2726141T	South Africa	Cohen, K.A., et al. PLoS medicine 12.9 (2015): e1001880.
SRR1011462	rpoB_D435G,rpoB_L452P	kasA_G269S,katG_S315T,fabG1_promoter_T1673432A	South Africa	Cohen, K.A., et al. PLoS medicine 12.9 (2015): e1001880.
SRR1011463	rpoB_D435G,rpoB_L452P	kasA_G269S,katG_S315T,fabG1_promoter_T1673432A	South Africa	Cohen, K.A., et al. PLoS medicine 12.9 (2015): e1001880.
SRR1011466	rpoB_D435G,rpoB_L452P	kasA_G269S,katG_S315T,fabG1_promoter_T1673432A	South Africa	Cohen, K.A., et al. PLoS medicine 12.9 (2015): e1001880.
SRR1011467	rpoB_D435G,rpoB_L452P	katG_S315T,fabG1_promoter_T1673432A	South Africa	Cohen, K.A., et al. PLoS medicine 12.9 (2015): e1001880.
SRR1011468	rpoB_D435G,rpoB_L452P	katG_S315T,fabG1_promoter_T1673432A	South Africa	Cohen, K.A., et al. PLoS medicine 12.9 (2015): e1001880.
SRR1011471	rpoB_L452P	katG_S315T,fabG1_promoter_T1673432A	South Africa	Cohen, K.A., et al. PLoS medicine 12.9 (2015): e1001880.
SRR1011473	rpoB_S450L	inhA_I194T,fabG1_promoter_C1673425T	South Africa	Cohen, K.A., et al. PLoS medicine 12.9 (2015): e1001880.
SRR1011474	rpoB_D435G,rpoB_L452P	kasA_G269S,katG_S315T,fabG1_promoter_T1673432A	South Africa	Cohen, K.A., et al. PLoS medicine 12.9 (2015): e1001880.
SRR1011476	rpoB_S450L	katG_S315T,ahpC_promoter_C2726141T	South Africa	Cohen, K.A., et al. PLoS medicine 12.9 (2015): e1001880.

SRR1011478	rpoB_S450L	inhA_I194T,fabG1_promoter_C1673425T	South Africa	Cohen, K.A., et al. PLoS medicine 12.9 (2015): e1001880.
SRR1011479	rpoB_S450L	katG_S315T	South Africa	Cohen, K.A., et al. PLoS medicine 12.9 (2015): e1001880.
SRR1011480	rpoB_S450L	katG_S315T	South Africa	Cohen, K.A., et al. PLoS medicine 12.9 (2015): e1001880.
SRR1011482	rpoB_S450L	inhA_I194T,fabG1_promoter_C1673425T	South Africa	Cohen, K.A., et al. PLoS medicine 12.9 (2015): e1001880.
SRR1011484	rpoB_S450W	katG_S315T	South Africa	Cohen, K.A., et al. PLoS medicine 12.9 (2015): e1001880.
SRR1019137	rpoB_L430P	katG_S315T	South Africa	Cohen, K.A., et al. PLoS medicine 12.9 (2015): e1001880.
SRR1019139	rpoB_H445L	katG_S315T	South Africa	Cohen, K.A., et al. PLoS medicine 12.9 (2015): e1001880.
SRR1019140	rpoB_Q429H,rpoB_D435Y	ahpC_promoter_C2726141T	South Africa	Cohen, K.A., et al. PLoS medicine 12.9 (2015): e1001880.
SRR1019146	rpoB_L430P	katG_S315T	South Africa	Cohen, K.A., et al. PLoS medicine 12.9 (2015): e1001880.
SRR1019148	rpoB_S450L	katG_S315T	South Africa	Cohen, K.A., et al. PLoS medicine 12.9 (2015): e1001880.
SRR1019149	rpoB_H445L	katG_S315T	South Africa	Cohen, K.A., et al. PLoS medicine 12.9 (2015): e1001880.
SRR1019152	rpoB_S450L	katG_S315T	South Africa	Cohen, K.A., et al. PLoS medicine 12.9 (2015): e1001880.
SRR1019161	rpoB_D435V	katG_S315T,fabG1_promoter_T1673432A	South Africa	Cohen, K.A., et al. PLoS medicine 12.9 (2015): e1001880.
SRR1019170	rpoB_L430P,rpoB_D435Y	kasA_G269S,katG_S315T,ahpC_promoter_C2726141T	South Africa	Cohen, K.A., et al. PLoS medicine 12.9 (2015): e1001880.
SRR1019171	rpoB_D435G,rpoB_L452P	kasA_G269S,katG_S315T,fabG1_promoter_T1673432A	South Africa	Cohen, K.A., et al. PLoS medicine 12.9 (2015): e1001880.
SRR1019172	rpoB_D435G,rpoB_L452P	katG_S315T,fabG1_promoter_T1673432A	South Africa	Cohen, K.A., et al. PLoS medicine 12.9 (2015): e1001880.
SRR1062819	rpoB_S450L	inhA_I194T,fabG1_promoter_C1673425T	South Africa	Cohen, K.A., et al. PLoS medicine 12.9 (2015): e1001880.
SRR1062820	rpoB_S450L	katG_S315T,fabG1_promoter_C1673425T	South Africa	Cohen, K.A., et al. PLoS medicine 12.9 (2015): e1001880.
SRR1062821	rpoB_S450L	katG_S315T,fabG1_promoter_C1673425T	South Africa	Cohen, K.A., et al. PLoS medicine 12.9 (2015): e1001880.
SRR1062822	rpoB_D435G,rpoB_L452P	kasA_G269S,katG_S315T,fabG1_promoter_T1673432A	South Africa	Cohen, K.A., et al. PLoS medicine 12.9 (2015): e1001880.
SRR1062823	rpoB_D435G,rpoB_L452P	kasA_G269S,katG_S315T,fabG1_promoter_T1673432A	South Africa	Cohen, K.A., et al. PLoS medicine 12.9 (2015): e1001880.
SRR1062824	rpoB_D435G,rpoB_L452P	kasA_G269S,katG_S315T,fabG1_promoter_T1673432A	South Africa	Cohen, K.A., et al. PLoS medicine 12.9 (2015): e1001880.
SRR1062825	rpoB_D435G,rpoB_L452P	kasA_G269S,katG_S315T,fabG1_promoter_T1673432A	South Africa	Cohen, K.A., et al. PLoS medicine 12.9 (2015): e1001880.
SRR1062826	rpoB_D435G,rpoB_L452P	kasA_G269S,katG_S315T,fabG1_promoter_T1673432A	South Africa	Cohen, K.A., et al. PLoS medicine 12.9 (2015): e1001880.
SRR1062827	rpoB_D435G,rpoB_L452P	kasA_G269S,katG_S315T,fabG1_promoter_T1673432A	South Africa	Cohen, K.A., et al. PLoS medicine 12.9 (2015): e1001880.
SRR1062828	rpoB_D435G,rpoB_L452P	kasA_G269S,katG_S315T,fabG1_promoter_T1673432A	South Africa	Cohen, K.A., et al. PLoS medicine 12.9 (2015): e1001880.
SRR1062829	rpoB_S450L	katG_S315T	South Africa	Cohen, K.A., et al. PLoS medicine 12.9 (2015): e1001880.
SRR1062832	rpoB_S450L	katG_S315T	South Africa	Cohen, K.A., et al. PLoS medicine 12.9 (2015): e1001880.
SRR1062833	rpoB_S450L	kasA_G269S,katG_S315T,ahpC_promoter_C2726141T	South Africa	Cohen, K.A., et al. PLoS medicine 12.9 (2015): e1001880.
SRR1062834	rpoB_D435G,rpoB_L452P	kasA_G269S,katG_S315T,fabG1_promoter_T1673432A	South Africa	Cohen, K.A., et al. PLoS medicine 12.9 (2015): e1001880.
SRR1062835	rpoB_D435G,rpoB_L452P	kasA_G269S,katG_S315T,fabG1_promoter_T1673432A	South Africa	Cohen, K.A., et al. PLoS medicine 12.9 (2015): e1001880.
SRR1062836	rpoB_D435F	katG_S315T	South Africa	Cohen, K.A., et al. PLoS medicine 12.9 (2015): e1001880.
SRR1062837	rpoB_S450L	katG_S315T,fabG1_promoter_C1673425T	South Africa	Cohen, K.A., et al. PLoS medicine 12.9 (2015): e1001880.
SRR1062839	rpoB_D435G,rpoB_L452P	kasA_G269S,katG_S315T,fabG1_promoter_T1673432A	South Africa	Cohen, K.A., et al. PLoS medicine 12.9 (2015): e1001880.
SRR1062840	rpoB_D435G,rpoB_L452P	kasA_G269S,katG_S315T,fabG1_promoter_T1673432A	South Africa	Cohen, K.A., et al. PLoS medicine 12.9 (2015): e1001880.

SRR1062841	rpoB_S450L	katG_S315T,fabG1_promoter_C1673425T	South Africa	Cohen, K.A., et al. PLoS medicine 12.9 (2015): e1001880.
SRR1062842	rpoB_D435G,rpoB_L452P	kasA_G269S,katG_S315T,fabG1_promoter_T1673432A	South Africa	Cohen, K.A., et al. PLoS medicine 12.9 (2015): e1001880.
SRR1062843	rpoB_D435G,rpoB_L452P	kasA_G269S,katG_S315T,fabG1_promoter_T1673432A	South Africa	Cohen, K.A., et al. PLoS medicine 12.9 (2015): e1001880.
SRR1062844	rpoB_D435G,rpoB_L452P	kasA_G269S,katG_S315T,fabG1_promoter_T1673432A	South Africa	Cohen, K.A., et al. PLoS medicine 12.9 (2015): e1001880.
SRR1062845	rpoB_S450L	inhA_I194T,fabG1_promoter_C1673425T	South Africa	Cohen, K.A., et al. PLoS medicine 12.9 (2015): e1001880.
SRR1062846	rpoB_D435G,rpoB_L452P	kasA_G269S,katG_S315T,fabG1_promoter_T1673432A	South Africa	Cohen, K.A., et al. PLoS medicine 12.9 (2015): e1001880.
SRR1062847	rpoB_S450L	kasA_G269S,katG_S315T,ahpC_promoter_C2726141T	South Africa	Cohen, K.A., et al. PLoS medicine 12.9 (2015): e1001880.
SRR1062848	rpoB_D435V	katG_S315T	South Africa	Cohen, K.A., et al. PLoS medicine 12.9 (2015): e1001880.
SRR1062849	rpoB_S450L	inhA_I194T,fabG1_promoter_C1673425T	South Africa	Cohen, K.A., et al. PLoS medicine 12.9 (2015): e1001880.
SRR1062850	rpoB_S450L	katG_S315T	South Africa	Cohen, K.A., et al. PLoS medicine 12.9 (2015): e1001880.
SRR1062851	rpoB_S450L	katG_S315T	South Africa	Cohen, K.A., et al. PLoS medicine 12.9 (2015): e1001880.
SRR1062852	rpoB_S450L	katG_S315T	South Africa	Cohen, K.A., et al. PLoS medicine 12.9 (2015): e1001880.
SRR1062853	rpoB_S450L	inhA_I194T,fabG1_promoter_C1673425T	South Africa	Cohen, K.A., et al. PLoS medicine 12.9 (2015): e1001880.
SRR1062854	rpoB_S450L	kasA_G269S,katG_S315T,ahpC_promoter_C2726141T	South Africa	Cohen, K.A., et al. PLoS medicine 12.9 (2015): e1001880.
SRR1062855	rpoB_S450W	kasA_G269S,katG_S315T	South Africa	Cohen, K.A., et al. PLoS medicine 12.9 (2015): e1001880.
SRR1062856	rpoB_S450L	fabG1_promoter_C1673425T	South Africa	Cohen, K.A., et al. PLoS medicine 12.9 (2015): e1001880.
SRR1140898	rpoB_L430P	katG_S315T	South Africa	Cohen, K.A., et al. PLoS medicine 12.9 (2015): e1001880.
SRR1140899	rpoB_S450L	inhA_I21T,fabG1_promoter_C1673425T	South Africa	Cohen, K.A., et al. PLoS medicine 12.9 (2015): e1001880.
SRR1140901	rpoB_Q429H,rpoB_D435Y	ahpC_promoter_C2726141T	South Africa	Cohen, K.A., et al. PLoS medicine 12.9 (2015): e1001880.
SRR1140916	rpoB_S450L	katG_S315T	South Africa	Cohen, K.A., et al. PLoS medicine 12.9 (2015): e1001880.
SRR1140924	rpoB_S450L	inhA_I21T,fabG1_promoter_C1673425T	South Africa	Cohen, K.A., et al. PLoS medicine 12.9 (2015): e1001880.
SRR1140926	rpoB_S450L	katG_S315T	South Africa	Cohen, K.A., et al. PLoS medicine 12.9 (2015): e1001880.
SRR1140929	rpoB_S450L	katG_S315T	South Africa	Cohen, K.A., et al. PLoS medicine 12.9 (2015): e1001880.
SRR1140930	rpoB_L430P	katG_S315T	South Africa	Cohen, K.A., et al. PLoS medicine 12.9 (2015): e1001880.
SRR1140932	rpoB_L452P	kasA_G269S,katG_S315T,fabG1_promoter_T1673432A	South Africa	Cohen, K.A., et al. PLoS medicine 12.9 (2015): e1001880.
SRR1140934	rpoB_S450L	katG_S315T	South Africa	Cohen, K.A., et al. PLoS medicine 12.9 (2015): e1001880.
SRR1140936	rpoB_S450L	katG_S315T	South Africa	Cohen, K.A., et al. PLoS medicine 12.9 (2015): e1001880.
SRR1140938	rpoB_D435Y	katG_S315T,fabG1_promoter_C1673425T	South Africa	Cohen, K.A., et al. PLoS medicine 12.9 (2015): e1001880.
SRR1140941	rpoB_S450L	katG_S315T	South Africa	Cohen, K.A., et al. PLoS medicine 12.9 (2015): e1001880.
SRR1140943	rpoB_H445L	katG_S315T	South Africa	Cohen, K.A., et al. PLoS medicine 12.9 (2015): e1001880.
SRR1140944	rpoB_L430P,rpoB_D435Y	kasA_G269S,katG_S315T,ahpC_promoter_C2726141T	South Africa	Cohen, K.A., et al. PLoS medicine 12.9 (2015): e1001880.
SRR1140945	rpoB_S450L	katG_S315T	South Africa	Cohen, K.A., et al. PLoS medicine 12.9 (2015): e1001880.
SRR1140946	rpoB_S450L	kasA_G269S,katG_S315T,ahpC_promoter_C2726141T	South Africa	Cohen, K.A., et al. PLoS medicine 12.9 (2015): e1001880.
SRR1140947	rpoB_S450L	katG_S315T	South Africa	Cohen, K.A., et al. PLoS medicine 12.9 (2015): e1001880.
SRR1140949	rpoB_I491F	kasA_G269S,katG_S315T,fabG1_promoter_T1673432A	South Africa	Cohen, K.A., et al. PLoS medicine 12.9 (2015): e1001880.
SRR1140951	rpoB_S450L	katG_S315T,fabG1_promoter_C1673425T	South Africa	Cohen, K.A., et al. PLoS medicine 12.9 (2015): e1001880.
SRR1140952	rpoB_H445G	katG_D419H,fabG1_promoter_C1673425T	South Africa	Cohen, K.A., et al. PLoS medicine 12.9 (2015): e1001880.

SRR1140955	rpoB_S450L	kasA_G269S,katG_S315T,ahpC_promoter_C2726141T	South Africa	Cohen, K.A., et al. PLoS medicine 12.9 (2015): e1001880.
SRR1140957	rpoB_S450L	katG_S315T	South Africa	Cohen, K.A., et al. PLoS medicine 12.9 (2015): e1001880.
SRR1140958	rpoB_S450L	katG_S315T	South Africa	Cohen, K.A., et al. PLoS medicine 12.9 (2015): e1001880.
SRR1140960	rpoB_D435V	kasA_G269S,katG_S315T,fabG1_promoter_T1673432A	South Africa	Cohen, K.A., et al. PLoS medicine 12.9 (2015): e1001880.
SRR1140961	rpoB_H445L	katG_S315T	South Africa	Cohen, K.A., et al. PLoS medicine 12.9 (2015): e1001880.
SRR1140964	rpoB_L452P	inhA_I194T,fabG1_promoter_C1673425T	South Africa	Cohen, K.A., et al. PLoS medicine 12.9 (2015): e1001880.
SRR1140965	rpoB_S450L	katG_S315T,fabG1_promoter_C1673425T	South Africa	Cohen, K.A., et al. PLoS medicine 12.9 (2015): e1001880.
SRR1172749	rpoB_D435V	katG_S315T	South Africa	Cohen, K.A., et al. PLoS medicine 12.9 (2015): e1001880.
SRR1172782	rpoB_D435V	katG_S315T,fabG1_promoter_T1673432G	South Africa	Cohen, K.A., et al. PLoS medicine 12.9 (2015): e1001880.
SRR1173081	rpoB_D435V	katG_S315T,fabG1_promoter_T1673432G	South Africa	Cohen, K.A., et al. PLoS medicine 12.9 (2015): e1001880.
SRR1173120	rpoB_D435V	katG_S315T	South Africa	Cohen, K.A., et al. PLoS medicine 12.9 (2015): e1001880.
SRR832977	rpoB_S450L	inhA_I21T,fabG1_promoter_C1673425T	South Africa	Cohen, K.A., et al. PLoS medicine 12.9 (2015): e1001880.
SRR832978	rpoB_S450L	katG_S315T	South Africa	Cohen, K.A., et al. PLoS medicine 12.9 (2015): e1001880.
SRR832986	rpoB_D435V	katG_S315T	South Africa	Cohen, K.A., et al. PLoS medicine 12.9 (2015): e1001880.
SRR832988	rpoB_S450L	katG_S315T	South Africa	Cohen, K.A., et al. PLoS medicine 12.9 (2015): e1001880.
SRR832989	rpoB_S450L	inhA_I194T	South Africa	Cohen, K.A., et al. PLoS medicine 12.9 (2015): e1001880.
SRR832991	rpoB_D435G,rpoB_L452P	kasA_G269S,katG_S315T,fabG1_promoter_T1673432A	South Africa	Cohen, K.A., et al. PLoS medicine 12.9 (2015): e1001880.
SRR832992	rpoB_S450L	inhA_I194T	South Africa	Cohen, K.A., et al. PLoS medicine 12.9 (2015): e1001880.
SRR832993	rpoB_S450L	katG_S315T	South Africa	Cohen, K.A., et al. PLoS medicine 12.9 (2015): e1001880.
SRR832994	rpoB_D435V	katG_S315T	South Africa	Cohen, K.A., et al. PLoS medicine 12.9 (2015): e1001880.
SRR832995	rpoB_L452P	kasA_G269S,katG_S315T,fabG1_promoter_T1673432A	South Africa	Cohen, K.A., et al. PLoS medicine 12.9 (2015): e1001880.
SRR832997	rpoB_S450L	inhA_I21T,fabG1_promoter_C1673425T	South Africa	Cohen, K.A., et al. PLoS medicine 12.9 (2015): e1001880.
SRR833000	rpoB_S450L	katG_S315T	South Africa	Cohen, K.A., et al. PLoS medicine 12.9 (2015): e1001880.
SRR833001	rpoB_S450L	katG_S315N	South Africa	Cohen, K.A., et al. PLoS medicine 12.9 (2015): e1001880.
SRR833008	rpoB_S441L	katG_S315T	South Africa	Cohen, K.A., et al. PLoS medicine 12.9 (2015): e1001880.
SRR833010	rpoB_S450L,rpoB_I480V	katG_S315T	South Africa	Cohen, K.A., et al. PLoS medicine 12.9 (2015): e1001880.
SRR833013	rpoB_D435G,rpoB_L452P	kasA_G269S,katG_S315T,fabG1_promoter_T1673432A	South Africa	Cohen, K.A., et al. PLoS medicine 12.9 (2015): e1001880.
SRR833017	rpoB_S450L	inhA_I21T,fabG1_promoter_C1673425T	South Africa	Cohen, K.A., et al. PLoS medicine 12.9 (2015): e1001880.
SRR833018	rpoB_S450L	inhA_I21T,fabG1_promoter_C1673425T	South Africa	Cohen, K.A., et al. PLoS medicine 12.9 (2015): e1001880.
SRR833019	rpoB_S450L	inhA_I194T	South Africa	Cohen, K.A., et al. PLoS medicine 12.9 (2015): e1001880.
SRR833021	rpoB_S450L,rpoB_I480V	katG_S315T	South Africa	Cohen, K.A., et al. PLoS medicine 12.9 (2015): e1001880.
SRR833023	rpoB_D435G,rpoB_L452P	kasA_G269S,katG_S315T,fabG1_promoter_T1673432A	South Africa	Cohen, K.A., et al. PLoS medicine 12.9 (2015): e1001880.
SRR833024	rpoB_D435G,rpoB_L452P	kasA_G269S,katG_S315T,fabG1_promoter_T1673432A	South Africa	Cohen, K.A., et al. PLoS medicine 12.9 (2015): e1001880.
SRR833027	rpoB_D435V	katG_S315T,fabG1_promoter_G1673423T	South Africa	Cohen, K.A., et al. PLoS medicine 12.9 (2015): e1001880.
SRR833028	rpoB_D435V	katG_S315T,fabG1_promoter_C1673425T	South Africa	Cohen, K.A., et al. PLoS medicine 12.9 (2015): e1001880.
SRR833029	rpoB_D435G,rpoB_L452P	kasA_G269S,katG_S315T,fabG1_promoter_T1673432A	South Africa	Cohen, K.A., et al. PLoS medicine 12.9 (2015): e1001880.
SRR833030	rpoB_S450L	katG_S315T	South Africa	Cohen, K.A., et al. PLoS medicine 12.9 (2015): e1001880.
SRR833033	rpoB_D435G,rpoB_L452P	kasA_G269S,katG_S315T,fabG1_promoter_T1673432A	South Africa	Cohen, K.A., et al. PLoS medicine 12.9 (2015): e1001880.
SRR833034	rpoB_D435F	katG_S315T	South Africa	Cohen, K.A., et al. PLoS medicine 12.9 (2015): e1001880.
SRR833037	rpoB_S450L	kasA_G269S,katG_S315T,ahpC_promoter_C2726141T	South Africa	Cohen, K.A., et al. PLoS medicine 12.9 (2015): e1001880.

SRR833038	rpoB_H445Y	katG_S315T,fabG1_promoter_C1673425T	South Africa	Cohen, K.A., et al. PLoS medicine 12.9 (2015): e1001880.
SRR833040	rpoB_L452P	kasA_G269S,katG_S315T,fabG1_promoter_T1673432A	South Africa	Cohen, K.A., et al. PLoS medicine 12.9 (2015): e1001880.
SRR833044	rpoB_H445D	katG_S315T	South Africa	Cohen, K.A., et al. PLoS medicine 12.9 (2015): e1001880.
SRR833047	rpoB_S450L	katG_S315T	South Africa	Cohen, K.A., et al. PLoS medicine 12.9 (2015): e1001880.
SRR833048	rpoB_S450W	kasA_G269S,katG_S315T	South Africa	Cohen, K.A., et al. PLoS medicine 12.9 (2015): e1001880.
SRR833056	rpoB_S450L	katG_S315T,fabG1_promoter_C1673425T	South Africa	Cohen, K.A., et al. PLoS medicine 12.9 (2015): e1001880.
SRR833058	rpoB_D435G,rpoB_L452P	kasA_G269S,katG_S315T,fabG1_promoter_T1673432A	South Africa	Cohen, K.A., et al. PLoS medicine 12.9 (2015): e1001880.
SRR833059	rpoB_D435G,rpoB_L452P	kasA_G269S,katG_S315T,fabG1_promoter_T1673432A	South Africa	Cohen, K.A., et al. PLoS medicine 12.9 (2015): e1001880.
SRR833061	rpoB_D435Y	katG_S315T	South Africa	Cohen, K.A., et al. PLoS medicine 12.9 (2015): e1001880.
SRR833062	rpoB_S450L	kasA_G269S,katG_S315T,fabG1_promoter_T1673432A	South Africa	Cohen, K.A., et al. PLoS medicine 12.9 (2015): e1001880.
SRR833064	rpoB_D435G,rpoB_L452P	kasA_G269S,katG_S315T,fabG1_promoter_T1673432A	South Africa	Cohen, K.A., et al. PLoS medicine 12.9 (2015): e1001880.
SRR833065	rpoB_S450L	katG_S315T	South Africa	Cohen, K.A., et al. PLoS medicine 12.9 (2015): e1001880.
SRR833067	rpoB_S450L	kasA_G269S,katG_S315T,ahpC_promoter_C2726141T	South Africa	Cohen, K.A., et al. PLoS medicine 12.9 (2015): e1001880.
SRR833068	rpoB_S450L	katG_S315T,fabG1_promoter_C1673425T	South Africa	Cohen, K.A., et al. PLoS medicine 12.9 (2015): e1001880.
SRR833071	rpoB_S450L	inhA_I194T	South Africa	Cohen, K.A., et al. PLoS medicine 12.9 (2015): e1001880.
SRR833073	rpoB_S441L	katG_S315T	South Africa	Cohen, K.A., et al. PLoS medicine 12.9 (2015): e1001880.
SRR833074	rpoB_S450L	kasA_G269S,katG_S315T,fabG1_promoter_T1673432A	South Africa	Cohen, K.A., et al. PLoS medicine 12.9 (2015): e1001880.
SRR833075	rpoB_H445Y	katG_S315T,fabG1_promoter_C1673425T	South Africa	Cohen, K.A., et al. PLoS medicine 12.9 (2015): e1001880.
SRR833076	rpoB_S450L	katG_S315T	South Africa	Cohen, K.A., et al. PLoS medicine 12.9 (2015): e1001880.
SRR833077	rpoB_D435G,rpoB_L452P	kasA_G269S,katG_S315T,fabG1_promoter_T1673432A	South Africa	Cohen, K.A., et al. PLoS medicine 12.9 (2015): e1001880.
SRR833078	rpoB_D435G,rpoB_L452P	kasA_G269S,katG_S315T,fabG1_promoter_T1673432A	South Africa	Cohen, K.A., et al. PLoS medicine 12.9 (2015): e1001880.
SRR833079	rpoB_S441L	katG_S315T	South Africa	Cohen, K.A., et al. PLoS medicine 12.9 (2015): e1001880.
SRR833080	rpoB_S450W	kasA_G269S,katG_S315T	South Africa	Cohen, K.A., et al. PLoS medicine 12.9 (2015): e1001880.
SRR833081	rpoB_S450L	katG_S315T	South Africa	Cohen, K.A., et al. PLoS medicine 12.9 (2015): e1001880.
SRR833085	rpoB_H445Y	katG_S315T,katG_S315R,katG_S315T	South Africa	Cohen, K.A., et al. PLoS medicine 12.9 (2015): e1001880.
SRR833086	rpoB_D435G,rpoB_L452P	kasA_G269S,katG_S315T,fabG1_promoter_T1673432A	South Africa	Cohen, K.A., et al. PLoS medicine 12.9 (2015): e1001880.
SRR833089	rpoB_S450L	inhA_I194T	South Africa	Cohen, K.A., et al. PLoS medicine 12.9 (2015): e1001880.
SRR833091	rpoB_D435V	katG_S315T	South Africa	Cohen, K.A., et al. PLoS medicine 12.9 (2015): e1001880.
SRR833092	rpoB_S450L	fabG1_promoter_C1673425T	South Africa	Cohen, K.A., et al. PLoS medicine 12.9 (2015): e1001880.
SRR833096	rpoB_D435Y	katG_S315T	South Africa	Cohen, K.A., et al. PLoS medicine 12.9 (2015): e1001880.
SRR833097	rpoB_S450W	kasA_G269S,katG_S315T	South Africa	Cohen, K.A., et al. PLoS medicine 12.9 (2015): e1001880.
SRR833099	rpoB_L452P	kasA_G269S,katG_S315T,fabG1_promoter_T1673432A	South Africa	Cohen, K.A., et al. PLoS medicine 12.9 (2015): e1001880.
SRR833100	rpoB_I491F	kasA_G269S,katG_S315T,fabG1_promoter_T1673432A	South Africa	Cohen, K.A., et al. PLoS medicine 12.9 (2015): e1001880.
SRR833105	rpoB_D435G,rpoB_L452P	kasA_G269S,katG_S315T,fabG1_promoter_T1673432A	South Africa	Cohen, K.A., et al. PLoS medicine 12.9 (2015): e1001880.
SRR833106	rpoB_S450L	kasA_G269S,katG_S315T,ahpC_promoter_C2726141T	South Africa	Cohen, K.A., et al. PLoS medicine 12.9 (2015): e1001880.

SRR833108	rpoB_S450L	katG_S315T	South Africa	Cohen, K.A., et al. PLoS medicine 12.9 (2015): e1001880.
SRR833109	rpoB_S450L	katG_S315T	South Africa	Cohen, K.A., et al. PLoS medicine 12.9 (2015): e1001880.
SRR833110	rpoB_L452P	kasA_G269S,katG_S315T,fabG1_promoter_T1673432A	South Africa	Cohen, K.A., et al. PLoS medicine 12.9 (2015): e1001880.
SRR833114	rpoB_L452P	kasA_G269S,katG_S315T,fabG1_promoter_T1673432A	South Africa	Cohen, K.A., et al. PLoS medicine 12.9 (2015): e1001880.
SRR833115	rpoB_D435G,rpoB_L452P	kasA_G269S,katG_S315T,fabG1_promoter_T1673432A	South Africa	Cohen, K.A., et al. PLoS medicine 12.9 (2015): e1001880.
SRR833117	rpoB_S450L	inhA_I21T,fabG1_promoter_C1673425T	South Africa	Cohen, K.A., et al. PLoS medicine 12.9 (2015): e1001880.
SRR833119	rpoB_S450L	katG_S315T	South Africa	Cohen, K.A., et al. PLoS medicine 12.9 (2015): e1001880.
SRR833120	rpoB_S450L	katG_S315T	South Africa	Cohen, K.A., et al. PLoS medicine 12.9 (2015): e1001880.
SRR833121	rpoB_D435G,rpoB_L452P	kasA_G269S,katG_S315T,fabG1_promoter_T1673432A	South Africa	Cohen, K.A., et al. PLoS medicine 12.9 (2015): e1001880.
SRR833124	rpoB_L452P	kasA_G269S,katG_S315T,fabG1_promoter_T1673432A	South Africa	Cohen, K.A., et al. PLoS medicine 12.9 (2015): e1001880.
SRR833126	rpoB_S450L	inhA_I194T	South Africa	Cohen, K.A., et al. PLoS medicine 12.9 (2015): e1001880.
SRR833129	rpoB_L430P	katG_S315T	South Africa	Cohen, K.A., et al. PLoS medicine 12.9 (2015): e1001880.
SRR833130	rpoB_D435G,rpoB_L452P	kasA_G269S,katG_S315T,fabG1_promoter_T1673432A	South Africa	Cohen, K.A., et al. PLoS medicine 12.9 (2015): e1001880.
SRR833131	rpoB_H445Y	katG_S315T,fabG1_promoter_C1673425T	South Africa	Cohen, K.A., et al. PLoS medicine 12.9 (2015): e1001880.
SRR833133	rpoB_S450L,rpoB_I480V	katG_S315T	South Africa	Cohen, K.A., et al. PLoS medicine 12.9 (2015): e1001880.
SRR833134	rpoB_S450L	katG_S315T	South Africa	Cohen, K.A., et al. PLoS medicine 12.9 (2015): e1001880.
SRR833137	rpoB_L452P	kasA_G269S,katG_S315T,fabG1_promoter_T1673432A	South Africa	Cohen, K.A., et al. PLoS medicine 12.9 (2015): e1001880.
SRR833139	rpoB_S450L	katG_S315T	South Africa	Cohen, K.A., et al. PLoS medicine 12.9 (2015): e1001880.
SRR833140	rpoB_S450L	kasA_G269S,katG_S315T,ahpC_promoter_C2726141T	South Africa	Cohen, K.A., et al. PLoS medicine 12.9 (2015): e1001880.
SRR833142	rpoB_D435V	katG_S315T,fabG1_promoter_G1673423T	South Africa	Cohen, K.A., et al. PLoS medicine 12.9 (2015): e1001880.
SRR833144	rpoB_S450L	katG_S315T	South Africa	Cohen, K.A., et al. PLoS medicine 12.9 (2015): e1001880.
SRR833145	rpoB_S450L	fabG1_promoter_C1673425T	South Africa	Cohen, K.A., et al. PLoS medicine 12.9 (2015): e1001880.
SRR833146	rpoB_L430P	katG_S315T	South Africa	Cohen, K.A., et al. PLoS medicine 12.9 (2015): e1001880.
SRR833147	rpoB_H445Y	katG_S315T,katG_S315R,katG_S315T	South Africa	Cohen, K.A., et al. PLoS medicine 12.9 (2015): e1001880.
SRR833148	rpoB_S450L	katG_S315T	South Africa	Cohen, K.A., et al. PLoS medicine 12.9 (2015): e1001880.
SRR833149	rpoB_S450L	kasA_G269S,katG_S315T,ahpC_promoter_C2726141T	South Africa	Cohen, K.A., et al. PLoS medicine 12.9 (2015): e1001880.
SRR833150	rpoB_D435Y	katG_S315T,fabG1_promoter_C1673425T	South Africa	Cohen, K.A., et al. PLoS medicine 12.9 (2015): e1001880.
SRR833157	rpoB_S450L	inhA_I194T	South Africa	Cohen, K.A., et al. PLoS medicine 12.9 (2015): e1001880.
SRR833162	rpoB_S450L	kasA_G269S,katG_S315T,ahpC_promoter_C2726141T	South Africa	Cohen, K.A., et al. PLoS medicine 12.9 (2015): e1001880.
SRR833163	rpoB_S450L	kasA_G269S,katG_S315T,fabG1_promoter_T1673432A	South Africa	Cohen, K.A., et al. PLoS medicine 12.9 (2015): e1001880.
SRR833165	rpoB_H445L	katG_S315T	South Africa	Cohen, K.A., et al. PLoS medicine 12.9 (2015): e1001880.
SRR833168	rpoB_S450L	katG_S315N	South Africa	Cohen, K.A., et al. PLoS medicine 12.9 (2015): e1001880.
SRR833169	rpoB_S450L,rpoB_I480V	katG_S315T	South Africa	Cohen, K.A., et al. PLoS medicine 12.9 (2015): e1001880.
SRR833172	rpoB_D435V	katG_S315T	South Africa	Cohen, K.A., et al. PLoS medicine 12.9 (2015): e1001880.
SRR833173	rpoB_D435G,rpoB_L452P	kasA_G269S,katG_S315T,fabG1_promoter_T1673432A	South Africa	Cohen, K.A., et al. PLoS medicine 12.9 (2015): e1001880.
SRR833174	rpoB_H445L	katG_S315T	South Africa	Cohen, K.A., et al. PLoS medicine 12.9 (2015): e1001880.
SRR833175	rpoB_D435F	katG_S315T	South Africa	Cohen, K.A., et al. PLoS medicine 12.9 (2015): e1001880.
SRR833177	rpoB_S450W	kasA_G269S,katG_S315T	South Africa	Cohen, K.A., et al. PLoS medicine 12.9 (2015): e1001880.
SRR833178	rpoB_S450L	katG_S315T	South Africa	Cohen, K.A., et al. PLoS medicine 12.9 (2015): e1001880.

SRR833186	rpoB_S450L	kasA_G269S,katG_S315T,fabG1_promoter_T1673432A	South Africa	Cohen, K.A., et al. PLoS medicine 12.9 (2015): e1001880.
SRR833188	rpoB_D435V	katG_S315T,fabG1_promoter_C1673425T	South Africa	Cohen, K.A., et al. PLoS medicine 12.9 (2015): e1001880.
SRR833191	rpoB_D435G,rpoB_L452P	kasA_G269S,katG_S315T,fabG1_promoter_T1673432A	South Africa	Cohen, K.A., et al. PLoS medicine 12.9 (2015): e1001880.
SRR833194	rpoB_H445Y	katG_S315T,fabG1_promoter_C1673425T	South Africa	Cohen, K.A., et al. PLoS medicine 12.9 (2015): e1001880.
SRR833196	rpoB_I491F	kasA_G269S,katG_S315T,fabG1_promoter_T1673432A	South Africa	Cohen, K.A., et al. PLoS medicine 12.9 (2015): e1001880.
SRR847775	rpoB_S450L	kasA_G269S,katG_S315T,ahpC_promoter_C2726141T	South Africa	Cohen, K.A., et al. PLoS medicine 12.9 (2015): e1001880.
SRR847776	rpoB_D435G,rpoB_L452P	kasA_G269S,katG_S315T,fabG1_promoter_T1673432A	South Africa	Cohen, K.A., et al. PLoS medicine 12.9 (2015): e1001880.
SRR847777	rpoB_D435G,rpoB_L452P	kasA_G269S,katG_S315T,fabG1_promoter_T1673432A	South Africa	Cohen, K.A., et al. PLoS medicine 12.9 (2015): e1001880.
SRR847778	rpoB_D435G,rpoB_L452P	kasA_G269S,katG_S315T,fabG1_promoter_T1673432A	South Africa	Cohen, K.A., et al. PLoS medicine 12.9 (2015): e1001880.
SRR847779	rpoB_D435G,rpoB_L452P	kasA_G269S,katG_S315T,fabG1_promoter_T1673432A	South Africa	Cohen, K.A., et al. PLoS medicine 12.9 (2015): e1001880.
SRR847780	rpoB_H445L	katG_S315T	South Africa	Cohen, K.A., et al. PLoS medicine 12.9 (2015): e1001880.
SRR847781	rpoB_L452P	kasA_G269S,katG_S315T,fabG1_promoter_T1673432A	South Africa	Cohen, K.A., et al. PLoS medicine 12.9 (2015): e1001880.
SRR847782	rpoB_S450L	kasA_G269S,katG_S315T,ahpC_promoter_C2726141T	South Africa	Cohen, K.A., et al. PLoS medicine 12.9 (2015): e1001880.
SRR847783	rpoB_D435G,rpoB_L452P	kasA_G269S,katG_S315T,fabG1_promoter_T1673432A	South Africa	Cohen, K.A., et al. PLoS medicine 12.9 (2015): e1001880.
SRR847784	rpoB_S450W	kasA_G269S,katG_S315T	South Africa	Cohen, K.A., et al. PLoS medicine 12.9 (2015): e1001880.
SRR847785	rpoB_D435G,rpoB_L452P	kasA_G269S,katG_S315T,fabG1_promoter_T1673432A	South Africa	Cohen, K.A., et al. PLoS medicine 12.9 (2015): e1001880.
SRR847786	rpoB_S450L	katG_S315T	South Africa	Cohen, K.A., et al. PLoS medicine 12.9 (2015): e1001880.
SRR847787	rpoB_D435G,rpoB_L452P	kasA_G269S,katG_S315T,fabG1_promoter_T1673432A	South Africa	Cohen, K.A., et al. PLoS medicine 12.9 (2015): e1001880.
SRR847788	rpoB_D435G,rpoB_L452P	kasA_G269S,katG_S315T,fabG1_promoter_T1673432A	South Africa	Cohen, K.A., et al. PLoS medicine 12.9 (2015): e1001880.
SRR847789	rpoB_D435G,rpoB_L452P	kasA_G269S,katG_S315T,fabG1_promoter_T1673432A	South Africa	Cohen, K.A., et al. PLoS medicine 12.9 (2015): e1001880.
SRR847790	rpoB_S450L	katG_S315T,fabG1_promoter_C1673425T	South Africa	Cohen, K.A., et al. PLoS medicine 12.9 (2015): e1001880.
SRR847791	rpoB_D435G,rpoB_L452P	kasA_G269S,katG_S315T,fabG1_promoter_T1673432A	South Africa	Cohen, K.A., et al. PLoS medicine 12.9 (2015): e1001880.
SRR847792	rpoB_D435G,rpoB_L452P	kasA_G269S,katG_S315T,fabG1_promoter_T1673432A	South Africa	Cohen, K.A., et al. PLoS medicine 12.9 (2015): e1001880.
SRR847793	rpoB_D435G,rpoB_L452P	kasA_G269S,katG_S315T,fabG1_promoter_T1673432A	South Africa	Cohen, K.A., et al. PLoS medicine 12.9 (2015): e1001880.
SRR847794	rpoB_D435G,rpoB_L452P	kasA_G269S,katG_S315T,fabG1_promoter_T1673432A	South Africa	Cohen, K.A., et al. PLoS medicine 12.9 (2015): e1001880.
SRR847795	rpoB_S450L	katG_S315T	South Africa	Cohen, K.A., et al. PLoS medicine 12.9 (2015): e1001880.
SRR847796	rpoB_D435G,rpoB_L452P	kasA_G269S,katG_S315T,fabG1_promoter_T1673432A	South Africa	Cohen, K.A., et al. PLoS medicine 12.9 (2015): e1001880.
SRR847797	rpoB_S450L	inhA_I21T,fabG1_promoter_C1673425T	South Africa	Cohen, K.A., et al. PLoS medicine 12.9 (2015): e1001880.

SRR847798	rpoB_S450W	katG_S315T	South Africa	Cohen, K.A., et al. PLoS medicine 12.9 (2015): e1001880.
SRR847799	rpoB_D435V	katG_S315T,fabG1_promoter_G1673423T	South Africa	Cohen, K.A., et al. PLoS medicine 12.9 (2015): e1001880.
SRR847800	rpoB_D435G,rpoB_L452P	kasA_G269S,katG_S315T,fabG1_promoter_T1673432A	South Africa	Cohen, K.A., et al. PLoS medicine 12.9 (2015): e1001880.
SRR847801	rpoB_D435G,rpoB_L452P	kasA_G269S,katG_S315T,fabG1_promoter_T1673432A	South Africa	Cohen, K.A., et al. PLoS medicine 12.9 (2015): e1001880.
SRR847802	rpoB_S450L	katG_S315T	South Africa	Cohen, K.A., et al. PLoS medicine 12.9 (2015): e1001880.
SRR847803	rpoB_S450L	katG_S315T	South Africa	Cohen, K.A., et al. PLoS medicine 12.9 (2015): e1001880.
SRR924205	rpoB_D435G,rpoB_L452P	kasA_G269S,katG_S315T,fabG1_promoter_T1673432A	South Africa	Cohen, K.A., et al. PLoS medicine 12.9 (2015): e1001880.
SRR924206	rpoB_D435G,rpoB_L452P	kasA_G269S,katG_S315T,fabG1_promoter_T1673432A	South Africa	Cohen, K.A., et al. PLoS medicine 12.9 (2015): e1001880.
SRR924210	rpoB_S450L	fabG1_promoter_C1673425T	South Africa	Cohen, K.A., et al. PLoS medicine 12.9 (2015): e1001880.
SRR924211	rpoB_L452P	katG_S315T,fabG1_promoter_T1673432A	South Africa	Cohen, K.A., et al. PLoS medicine 12.9 (2015): e1001880.
SRR924212	rpoB_D435G,rpoB_L452P	kasA_G269S,katG_S315T,fabG1_promoter_T1673432A	South Africa	Cohen, K.A., et al. PLoS medicine 12.9 (2015): e1001880.
SRR924214	rpoB_L452P	kasA_G269S,katG_S315T,fabG1_promoter_T1673432A	South Africa	Cohen, K.A., et al. PLoS medicine 12.9 (2015): e1001880.
SRR924219	rpoB_D435G,rpoB_L452P	kasA_G269S,katG_S315T,fabG1_promoter_T1673432A	South Africa	Cohen, K.A., et al. PLoS medicine 12.9 (2015): e1001880.
SRR924224	rpoB_S450L	kasA_G269S,katG_S315T,ahpC_promoter_C2726141T	South Africa	Cohen, K.A., et al. PLoS medicine 12.9 (2015): e1001880.
SRR924225	rpoB_D435G,rpoB_L452P	kasA_G269S,katG_S315T,fabG1_promoter_T1673432A	South Africa	Cohen, K.A., et al. PLoS medicine 12.9 (2015): e1001880.
SRR924226	rpoB_D435G,rpoB_L452P	katG_S315T,fabG1_promoter_T1673432A	South Africa	Cohen, K.A., et al. PLoS medicine 12.9 (2015): e1001880.
SRR924227	rpoB_D435G,rpoB_L452P	kasA_G269S,katG_S315T,fabG1_promoter_T1673432A	South Africa	Cohen, K.A., et al. PLoS medicine 12.9 (2015): e1001880.
SRR924231	rpoB_D435G,rpoB_L452P	kasA_G269S,katG_S315T,fabG1_promoter_T1673432A	South Africa	Cohen, K.A., et al. PLoS medicine 12.9 (2015): e1001880.
SRR924232	rpoB_D435G,rpoB_L452P	kasA_G269S,katG_S315T,fabG1_promoter_T1673432A	South Africa	Cohen, K.A., et al. PLoS medicine 12.9 (2015): e1001880.
SRR924233	rpoB_S450L	kasA_G269S,katG_S315T,ahpC_promoter_C2726141T	South Africa	Cohen, K.A., et al. PLoS medicine 12.9 (2015): e1001880.
SRR924234	rpoB_D435G,rpoB_L452P	kasA_G269S,katG_S315T,fabG1_promoter_T1673432A	South Africa	Cohen, K.A., et al. PLoS medicine 12.9 (2015): e1001880.
SRR924240	rpoB_S450L	katG_S315T	South Africa	Cohen, K.A., et al. PLoS medicine 12.9 (2015): e1001880.
SRR924692	rpoB_D435G,rpoB_L452P	kasA_G269S,katG_S315T,fabG1_promoter_T1673432A	South Africa	Cohen, K.A., et al. PLoS medicine 12.9 (2015): e1001880.
SRR924694	rpoB_D435G,rpoB_L452P	kasA_G269S,katG_S315T,fabG1_promoter_T1673432A	South Africa	Cohen, K.A., et al. PLoS medicine 12.9 (2015): e1001880.
SRR924696	rpoB_L452P	kasA_G269S,katG_S315T,fabG1_promoter_T1673432A	South Africa	Cohen, K.A., et al. PLoS medicine 12.9 (2015): e1001880.
SRR924697	rpoB_D435G,rpoB_L452P	kasA_G269S,katG_S315T,fabG1_promoter_T1673432A	South Africa	Cohen, K.A., et al. PLoS medicine 12.9 (2015): e1001880.
SRR958195	rpoB_L452P	kasA_G269S,katG_S315T,fabG1_promoter_T1673432A	South Africa	Cohen, K.A., et al. PLoS medicine 12.9 (2015): e1001880.
SRR2101307	rpoB_H445D	katG_S315T	South Africa	Walker, T.M., et al. The Lancet infectious diseases 15.10 (2015): 1193-1202.
SRR2101344	rpoB_D435V	katG_S315T,fabG1_promoter_G1673423T	South Africa	Walker, T.M., et al. The Lancet infectious diseases 15.10 (2015): 1193-1202.

SRR2101373	rpoB_S450L	inhA_S94A	South Africa	Walker, T.M., et al. The Lancet infectious diseases 15.10 (2015): 1193-1202.
SRR2101384	rpoB_S450L	katG_S315T	South Africa	Walker, T.M., et al. The Lancet infectious diseases 15.10 (2015): 1193-1202.
SRR2101460	rpoB_S450L	fabG1_promoter_C1673425T	South Africa	Walker, T.M., et al. The Lancet infectious diseases 15.10 (2015): 1193-1202.
SRR2101470	rpoB_S450L	fabG1_promoter_C1673425T	South Africa	Walker, T.M., et al. The Lancet infectious diseases 15.10 (2015): 1193-1202.
SRR2101482	rpoB_H445Y	katG_S315T,fabG1_promoter_C1673425T	South Africa	Walker, T.M., et al. The Lancet infectious diseases 15.10 (2015): 1193-1202.
SRR2101505	rpoB_S450L	katG_S315T	South Africa	Walker, T.M., et al. The Lancet infectious diseases 15.10 (2015): 1193-1202.
SRR2101539	rpoB_L452P	kasA_G269S,katG_S315T,fabG1_promoter_T1673432A	South Africa	Walker, T.M., et al. The Lancet infectious diseases 15.10 (2015): 1193-1202.
SRR2101574	rpoB_H445Y	katG_S315T,fabG1_promoter_C1673425T	South Africa	Walker, T.M., et al. The Lancet infectious diseases 15.10 (2015): 1193-1202.
SRR2101686	rpoB_L430P	kasA_G269S,katG_S315T	South Africa	Walker, T.M., et al. The Lancet infectious diseases 15.10 (2015): 1193-1202.
SRR2101690	rpoB_F433L,rpoB_M434I,rpoB_D435H	katG_S315T	South Africa	Walker, T.M., et al. The Lancet infectious diseases 15.10 (2015): 1193-1202.
SRR2101731	rpoB_S450L	katG_S315T	South Africa	Walker, T.M., et al. The Lancet infectious diseases 15.10 (2015): 1193-1202.
SRR2101757	rpoB_S450L	inhA_I194T	South Africa	Walker, T.M., et al. The Lancet infectious diseases 15.10 (2015): 1193-1202.
SRR2101763	rpoB_S450L	inhA_I194T	South Africa	Walker, T.M., et al. The Lancet infectious diseases 15.10 (2015): 1193-1202.
SRR2101806	rpoB_H445D	katG_S315T	South Africa	Walker, T.M., et al. The Lancet infectious diseases 15.10 (2015): 1193-1202.
ERR970445	rpoB_S450L	fabG1_promoter_C1673425T	South Africa	Black, P. A., et al. BMC gemics 16.1 (2015): 857.
ERR970447	rpoB_S450L	fabG1_promoter_C1673425T	South Africa	Black, P. A., et al. BMC gemics 16.1 (2015): 857.
ERR972795	rpoB_S450L	fabG1_promoter_C1673425T	South Africa	Black, P. A., et al. BMC gemics 16.1 (2015): 857.
ERR983252	rpoB_S450L	fabG1_promoter_C1673425T	South Africa	Black, P. A., et al. BMC gemics 16.1 (2015): 857.
ERR983253	rpoB_S450L	fabG1_promoter_C1673425T	South Africa	Black, P. A., et al. BMC gemics 16.1 (2015): 857.
ERR983254	rpoB_S450L	fabG1_promoter_C1673425T	South Africa	Black, P. A., et al. BMC gemics 16.1 (2015): 857.
ERR550665	rpoB_S450L	katG_S315T	Sierra Leone	Walker, T.M., et al. The Lancet infectious diseases 15.10 (2015): 1193-1202.
ERR551126	rpoB_S450L	katG_S315T	Sierra Leone	Walker, T.M., et al. The Lancet infectious diseases 15.10 (2015): 1193-1202.
ERR551513	rpoB_S450L	katG_S315T	Sierra Leone	Walker, T.M., et al. The Lancet infectious diseases 15.10 (2015): 1193-1202.
ERR551620	rpoB_D435Y	ahpC_promoter_G2726119A	Sierra Leone	Walker, T.M., et al. The Lancet infectious diseases 15.10 (2015): 1193-1202.
ERR552260	rpoB_D435Y	kasA_G269S,katG_S315T	Sierra Leone	Walker, T.M., et al. The Lancet infectious diseases 15.10 (2015): 1193-1202.
ERR552662	rpoB_S450L	katG_S315T	Sierra Leone	Walker, T.M., et al. The Lancet infectious diseases 15.10 (2015): 1193-1202.
ERR552979	rpoB_S450L	katG_S315T	Sierra Leone	Walker, T.M., et al. The Lancet infectious diseases 15.10 (2015): 1193-1202.
ERR553000	rpoB_H445Y	inhA_V78A,katG_S315T,ahpC_promoter_C2726141T	Sierra Leone	Walker, T.M., et al. The Lancet infectious diseases 15.10 (2015): 1193-1202.
ERR553037	rpoB_H445Y	katG_S315T	Sierra Leone	Walker, T.M., et al. The Lancet infectious diseases 15.10 (2015): 1193-1202.
ERR553226	rpoB_S450L	ahpC_promoter_C2726112T	Sierra Leone	Walker, T.M., et al. The Lancet infectious diseases 15.10 (2015): 1193-1202.
ERR464719	rpoB_H445Y	katG_S315T	Switzerland	Bryant, J.M., et al. BMC infectious diseases 13.1 (2013): 110.
ERR464729	rpoB_D435Y	katG_S315N	Switzerland	Bryant, J.M., et al. BMC infectious diseases 13.1 (2013): 110.
ERR464730	rpoB_D435Y	katG_S315N	Switzerland	Bryant, J.M., et al. BMC infectious diseases 13.1 (2013): 110.
SRR2100131	rpoB_L452P	katG_S315T	UK	Walker, T.M., et al. The Lancet infectious diseases 15.10 (2015): 1193-1202.
SRR2100132	rpoB_L452P	katG_S315T	UK	Walker, T.M., et al. The Lancet infectious diseases 15.10 (2015): 1193-1202.
ERR046786	rpoB_I491F	katG_V473F	UK	Walker, T.M., et al. The Lancet infectious diseases 13.2 (2013): 137-146.
ERR046796	rpoB_L452P	katG_S315T	UK	Walker, T.M., et al. The Lancet infectious diseases 13.2 (2013): 137-146.
ERR046821	rpoB_L452P	katG_S315T	UK	Walker, T.M., et al. The Lancet infectious diseases 13.2 (2013): 137-146.
ERR046855	rpoB_H445Y	katG_S315T	UK	Walker, T.M., et al. The Lancet infectious diseases 13.2 (2013): 137-146.
ERR046903	rpoB_L452P	katG_S315T	UK	Walker, T.M., et al. The Lancet infectious diseases 13.2 (2013): 137-146.
ERR046933	rpoB_V170F	inhA_S94A	UK	Walker, T.M., et al. The Lancet infectious diseases 13.2 (2013): 137-146.
SRR2100176	rpoB_H445N	katG_S315T	UK	Walker, T.M., et al. The Lancet infectious diseases 15.10 (2015): 1193-1202.
SRR2100228	rpoB_D435Y	katG_S315T	UK	Walker, T.M., et al. The Lancet infectious diseases 15.10 (2015): 1193-1202.
SRR2100231	rpoB_S450L	katG_S315T	UK	Walker, T.M., et al. The Lancet infectious diseases 15.10 (2015): 1193-1202.
SRR2100234	rpoB_H445D	katG_S315T	UK	Walker, T.M., et al. The Lancet infectious diseases 15.10 (2015): 1193-1202.
SRR2100238	rpoB_S450L	fabG1_promoter_C1673425T	UK	Walker, T.M., et al. The Lancet infectious diseases 15.10 (2015): 1193-1202.
SRR2100246	rpoB_S450L	katG_S315T	UK	Walker, T.M., et al. The Lancet infectious diseases 15.10 (2015): 1193-1202.
SRR2100250	rpoB_H445Y	katG_S315T	UK	Walker, T.M., et al. The Lancet infectious diseases 15.10 (2015): 1193-1202.
SRR2100257	rpoB_S450L	katG_S315T	UK	Walker, T.M., et al. The Lancet infectious diseases 15.10 (2015): 1193-1202.

SRR2100259	rpoB_H445N	fabG1_promoter_C1673425T	UK	Walker, T.M., et al. The Lancet infectious diseases 15.10 (2015): 1193-1202.
SRR2100267	rpoB_S450L	katG_S315T	UK	Walker, T.M., et al. The Lancet infectious diseases 15.10 (2015): 1193-1202.
SRR2100282	rpoB_H445Y	katG_S315T	UK	Walker, T.M., et al. The Lancet infectious diseases 15.10 (2015): 1193-1202.
SRR2100283	rpoB_I491F	kasA_G269S,katG_S315T	UK	Walker, T.M., et al. The Lancet infectious diseases 15.10 (2015): 1193-1202.
SRR2100294	rpoB_D435Y	katG_S315T	UK	Walker, T.M., et al. The Lancet infectious diseases 15.10 (2015): 1193-1202.
SRR2100301	rpoB_L430P,rpoB_S431G	katG_S315T	UK	Walker, T.M., et al. The Lancet infectious diseases 15.10 (2015): 1193-1202.
SRR2100306	rpoB_S450L,rpoB_I480V	katG_S315T	UK	Walker, T.M., et al. The Lancet infectious diseases 15.10 (2015): 1193-1202.
SRR2100324	rpoB_D435F	katG_S315T	UK	Walker, T.M., et al. The Lancet infectious diseases 15.10 (2015): 1193-1202.
SRR2100331	rpoB_S450L	katG_S315T	UK	Walker, T.M., et al. The Lancet infectious diseases 15.10 (2015): 1193-1202.
SRR2100349	rpoB_S450L	katG_S315T	UK	Walker, T.M., et al. The Lancet infectious diseases 15.10 (2015): 1193-1202.
SRR2100352	rpoB_S450L	inhA_I194T	UK	Walker, T.M., et al. The Lancet infectious diseases 15.10 (2015): 1193-1202.
SRR2100368	rpoB_S450L	katG_S315T	UK	Walker, T.M., et al. The Lancet infectious diseases 15.10 (2015): 1193-1202.
SRR2100376	rpoB_S450L	katG_S315T	UK	Walker, T.M., et al. The Lancet infectious diseases 15.10 (2015): 1193-1202.
SRR2100385	rpoB_S450W	katG_S315T	UK	Walker, T.M., et al. The Lancet infectious diseases 15.10 (2015): 1193-1202.
SRR2100392	rpoB_S450L	katG_S315T	UK	Walker, T.M., et al. The Lancet infectious diseases 15.10 (2015): 1193-1202.
SRR2100227	rpoB_S450L	katG_S315T,fabG1_promoter_T1673432G	UK	Walker, T.M., et al. The Lancet infectious diseases 15.10 (2015): 1193-1202.
SRR2100387	rpoB_S450L,rpoB_I480V	katG_S315T	UK	Walker, T.M., et al. The Lancet infectious diseases 15.10 (2015): 1193-1202.
SRR2100740	rpoB_S450L	katG_S315T	UK	Walker, T.M., et al. The Lancet infectious diseases 15.10 (2015): 1193-1202.
SRR2100803	rpoB_H445Y	fabG1_promoter_C1673425T	UK	Walker, T.M., et al. The Lancet infectious diseases 15.10 (2015): 1193-1202.
SRR2100828	rpoB_H445R	katG_S315T	UK	Walker, T.M., et al. The Lancet infectious diseases 15.10 (2015): 1193-1202.
SRR2100830	rpoB_S450L	katG_S315T	UK	Walker, T.M., et al. The Lancet infectious diseases 15.10 (2015): 1193-1202.
SRR2100836	rpoB_S450L	katG_S315T	UK	Walker, T.M., et al. The Lancet infectious diseases 15.10 (2015): 1193-1202.
SRR2100838	rpoB_S450L	katG_S315T	UK	Walker, T.M., et al. The Lancet infectious diseases 15.10 (2015): 1193-1202.
SRR2100842	rpoB_S450L	katG_S315T	UK	Walker, T.M., et al. The Lancet infectious diseases 15.10 (2015): 1193-1202.
SRR2100848	rpoB_S450L	katG_S315T	UK	Walker, T.M., et al. The Lancet infectious diseases 15.10 (2015): 1193-1202.
SRR2100854	rpoB_D435V	katG_S315T	UK	Walker, T.M., et al. The Lancet infectious diseases 15.10 (2015): 1193-1202.
SRR2100855	rpoB_D435V	katG_S315T	UK	Walker, T.M., et al. The Lancet infectious diseases 15.10 (2015): 1193-1202.
SRR2100869	rpoB_S450L	katG_S315T,ahpC_promoter_G2726145A	UK	Walker, T.M., et al. The Lancet infectious diseases 15.10 (2015): 1193-1202.
SRR2100870	rpoB_S450L,rpoB_S450F	katG_S315T	UK	Walker, T.M., et al. The Lancet infectious diseases 15.10 (2015): 1193-1202.
SRR2100873	rpoB_S450L,rpoB_S450F	katG_S315T	UK	Walker, T.M., et al. The Lancet infectious diseases 15.10 (2015): 1193-1202.
SRR2100884	rpoB_D435V	katG_S315T	UK	Walker, T.M., et al. The Lancet infectious diseases 15.10 (2015): 1193-1202.
SRR2100889	rpoB_S450L,rpoB_S450F	katG_S315T	UK	Walker, T.M., et al. The Lancet infectious diseases 15.10 (2015): 1193-1202.
SRR2100890	rpoB_S450L	fabG1_promoter_C1673425T	UK	Walker, T.M., et al. The Lancet infectious diseases 15.10 (2015): 1193-1202.
SRR2100891	rpoB_H445Y	fabG1_promoter_C1673425T	UK	Walker, T.M., et al. The Lancet infectious diseases 15.10 (2015): 1193-1202.
SRR2100904	rpoB_H445R	katG_S315T	UK	Walker, T.M., et al. The Lancet infectious diseases 15.10 (2015): 1193-1202.
SRR2100906	rpoB_H445Y	fabG1_promoter_C1673425T	UK	Walker, T.M., et al. The Lancet infectious diseases 15.10 (2015): 1193-1202.
SRR2100909	rpoB_S450L	katG_S315T	UK	Walker, T.M., et al. The Lancet infectious diseases 15.10 (2015): 1193-1202.
SRR2100911	rpoB_S450L,rpoB_S450F	katG_S315T	UK	Walker, T.M., et al. The Lancet infectious diseases 15.10 (2015): 1193-1202.
SRR2100912	rpoB_S450L	katG_S315T	UK	Walker, T.M., et al. The Lancet infectious diseases 15.10 (2015): 1193-1202.
SRR2100920	rpoB_S450L	katG_S315T	UK	Walker, T.M., et al. The Lancet infectious diseases 15.10 (2015): 1193-1202.
SRR2100921	rpoB_S450L	katG_S315T	UK	Walker, T.M., et al. The Lancet infectious diseases 15.10 (2015): 1193-1202.
SRR2100925	rpoB_S450L	katG_S315T	UK	Walker, T.M., et al. The Lancet infectious diseases 15.10 (2015): 1193-1202.
SRR2100950	rpoB_S450L,rpoB_I480V	katG_S315T	UK	Walker, T.M., et al. The Lancet infectious diseases 15.10 (2015): 1193-1202.
SRR2101021	rpoB_S450L	katG_S315T	UK	Walker, T.M., et al. The Lancet infectious diseases 15.10 (2015): 1193-1202.
SRR2101023	rpoB_S450L	katG_S315T	UK	Walker, T.M., et al. The Lancet infectious diseases 15.10 (2015): 1193-1202.
SRR2101053	rpoB_S450L	katG_S315T	UK	Walker, T.M., et al. The Lancet infectious diseases 15.10 (2015): 1193-1202.
SRR2101055	rpoB_S450L	katG_S315T	UK	Walker, T.M., et al. The Lancet infectious diseases 15.10 (2015): 1193-1202.
ERR192451	rpoB_S450L	katG_S315T	UK	Köser, C.U., et al. New England journal of medicine369.3 (2013): 290-292.
ERR198708	rpoB_S450L	katG_S315T	UK	Köser, C.U., et al. New England journal of medicine369.3 (2013): 290-292.
ERR553412	rpoB_L430P	katG_S315T	UK	Witney, A.A., et al. Journal of clinical microbiology 53.5 (2015): 1473-1483.
ERR553413	rpoB_L430P	katG_S315T	UK	Witney, A.A., et al. Journal of clinical microbiology 53.5 (2015): 1473-1483.
ERR553414	rpoB_S450L	katG_S315T,fabG1_promoter_C1673425T	UK	Witney, A.A., et al. Journal of clinical microbiology 53.5 (2015): 1473-1483.
ERR553415	rpoB_S450L	katG_S315T,fabG1_promoter_C1673425T	UK	Witney, A.A., et al. Journal of clinical microbiology 53.5 (2015): 1473-1483.
ERR553416	rpoB_S450L	katG_S315T	UK	Witney, A.A., et al. Journal of clinical microbiology 53.5 (2015): 1473-1483.
ERR553417	rpoB_S450L	katG_S315T	UK	Witney, A.A., et al. Journal of clinical microbiology 53.5 (2015): 1473-1483.
ERR553418	rpoB_S450L	katG_S315T	UK	Witney, A.A., et al. Journal of clinical microbiology 53.5 (2015): 1473-1483.
ERR553419	rpoB_S450L	katG_S315T	UK	Witney, A.A., et al. Journal of clinical microbiology 53.5 (2015): 1473-1483.

ERR553420	rpoB_S450L	katG_S315T	UK	Witney, A.A., et al. Journal of clinical microbiology 53.5 (2015): 1473-1483.
ERR553421	rpoB_S450L	katG_S315T	UK	Witney, A.A., et al. Journal of clinical microbiology 53.5 (2015): 1473-1483.
ERR553422	rpoB_S450L	katG_S315T	UK	Witney, A.A., et al. Journal of clinical microbiology 53.5 (2015): 1473-1483.
ERR553423	rpoB_S450L	katG_S315T	UK	Witney, A.A., et al. Journal of clinical microbiology 53.5 (2015): 1473-1483.
ERR553424	rpoB_S450L	katG_S315T	UK	Witney, A.A., et al. Journal of clinical microbiology 53.5 (2015): 1473-1483.
ERR553425	rpoB_S450L	katG_S315T	UK	Walker, T.M., et al. The Lancet infectious diseases 15.10 (2015): 1193-1202.
ERR553426	rpoB_S450L	katG_S315T	UK	Walker, T.M., et al. The Lancet infectious diseases 15.10 (2015): 1193-1202.
ERR553427	rpoB_S450L	katG_S315T	UK	Walker, T.M., et al. The Lancet infectious diseases 15.10 (2015): 1193-1202.
ERR400382	rpoB_S450L	katG_S315T	UK	Walker, T.M., et al. The Lancet infectious diseases 15.10 (2015): 1193-1202.
ERR400517	rpoB_H445N	katG_S315T	UK	Walker, T.M., et al. The Lancet infectious diseases 15.10 (2015): 1193-1202.
ERR400553	rpoB_S450L	inhA_I194T,fabG1_promoter_C1673425T	UK	Walker, T.M., et al. The Lancet infectious diseases 15.10 (2015): 1193-1202.
SRR2100400	rpoB_S450L	katG_S315T	UK	Walker, T.M., et al. The Lancet infectious diseases 15.10 (2015): 1193-1202.
SRR2100402	rpoB_S450L	katG_P241P,katG_S315T	UK	Walker, T.M., et al. The Lancet infectious diseases 15.10 (2015): 1193-1202.
SRR2100419	rpoB_S450L	katG_S315T	UK	Walker, T.M., et al. The Lancet infectious diseases 15.10 (2015): 1193-1202.
SRR2100429	rpoB_S450L	katG_S315T	UK	Walker, T.M., et al. The Lancet infectious diseases 15.10 (2015): 1193-1202.
SRR2100432	rpoB_S450L	katG_S315T	UK	Walker, T.M., et al. The Lancet infectious diseases 15.10 (2015): 1193-1202.
SRR2100459	rpoB_H445D	katG_S315T	UK	Walker, T.M., et al. The Lancet infectious diseases 15.10 (2015): 1193-1202.
ERR550812	rpoB_S450L	katG_S315T	Uzbekistan	Walker, T.M., et al. The Lancet infectious diseases 15.10 (2015): 1193-1202.
ERR551121	rpoB_S450L	katG_S315T	Uzbekistan	Walker, T.M., et al. The Lancet infectious diseases 15.10 (2015): 1193-1202.
ERR551159	rpoB_S450L	katG_S315N	Uzbekistan	Walker, T.M., et al. The Lancet infectious diseases 15.10 (2015): 1193-1202.
ERR551290	rpoB_S450L	katG_S315T	Uzbekistan	Walker, T.M., et al. The Lancet infectious diseases 15.10 (2015): 1193-1202.
ERR551430	rpoB_S450L	katG_S315T	Uzbekistan	Walker, T.M., et al. The Lancet infectious diseases 15.10 (2015): 1193-1202.
ERR551701	rpoB_S450L	katG_S315T	Uzbekistan	Walker, T.M., et al. The Lancet infectious diseases 15.10 (2015): 1193-1202.
ERR552047	rpoB_S450L	katG_S315T	Uzbekistan	Walker, T.M., et al. The Lancet infectious diseases 15.10 (2015): 1193-1202.
ERR552274	rpoB_S450L	katG_S315T	Uzbekistan	Walker, T.M., et al. The Lancet infectious diseases 15.10 (2015): 1193-1202.
ERR552358	rpoB_S450L	katG_S315T	Uzbekistan	Walker, T.M., et al. The Lancet infectious diseases 15.10 (2015): 1193-1202.
ERR552441	rpoB_S450L	katG_S315T	Uzbekistan	Walker, T.M., et al. The Lancet infectious diseases 15.10 (2015): 1193-1202.
ERR552517	rpoB_S450L	katG_S315T,fabG1_promoter_T1673432C	Uzbekistan	Walker, T.M., et al. The Lancet infectious diseases 15.10 (2015): 1193-1202.
ERR552580	rpoB_T400A,rpoB_S450L	katG_S315T	Uzbekistan	Walker, T.M., et al. The Lancet infectious diseases 15.10 (2015): 1193-1202.
ERR552677	rpoB_S450L	katG_S315T	Uzbekistan	Walker, T.M., et al. The Lancet infectious diseases 15.10 (2015): 1193-1202.
ERR552774	rpoB_T400A,rpoB_Q432P	katG_S315N	Uzbekistan	Walker, T.M., et al. The Lancet infectious diseases 15.10 (2015): 1193-1202.
ERR552933	rpoB_S450L	katG_S315T	Uzbekistan	Walker, T.M., et al. The Lancet infectious diseases 15.10 (2015): 1193-1202.
ERR552937	rpoB_S450L	katG_S315T	Uzbekistan	Walker, T.M., et al. The Lancet infectious diseases 15.10 (2015): 1193-1202.
ERR553068	rpoB_S450L	katG_S315T	Uzbekistan	Walker, T.M., et al. The Lancet infectious diseases 15.10 (2015): 1193-1202.
ERR553139	rpoB_S450L	katG_S315I	Uzbekistan	Walker, T.M., et al. The Lancet infectious diseases 15.10 (2015): 1193-1202.
ERR550646	rpoB_S450L	katG_S315N	Uzbekistan	Walker, T.M., et al. The Lancet infectious diseases 15.10 (2015): 1193-1202.
ERR550649	rpoB_H445Y	katG_S315T	Uzbekistan	Walker, T.M., et al. The Lancet infectious diseases 15.10 (2015): 1193-1202.
ERR550651	rpoB_S450L	katG_S315T	Uzbekistan	Walker, T.M., et al. The Lancet infectious diseases 15.10 (2015): 1193-1202.
ERR550664	rpoB_S450L	katG_S315T	Uzbekistan	Walker, T.M., et al. The Lancet infectious diseases 15.10 (2015): 1193-1202.
ERR550667	rpoB_S450L	katG_S315T	Uzbekistan	Walker, T.M., et al. The Lancet infectious diseases 15.10 (2015): 1193-1202.
ERR550676	rpoB_S450L	katG_S315T	Uzbekistan	Walker, T.M., et al. The Lancet infectious diseases 15.10 (2015): 1193-1202.
ERR550682	rpoB_S450L	katG_S315T	Uzbekistan	Walker, T.M., et al. The Lancet infectious diseases 15.10 (2015): 1193-1202.
ERR550685	rpoB_S450L	katG_R104Q	Uzbekistan	Walker, T.M., et al. The Lancet infectious diseases 15.10 (2015): 1193-1202.
ERR550706	rpoB_S450L	kasA_G269S,katG_S315T	Uzbekistan	Walker, T.M., et al. The Lancet infectious diseases 15.10 (2015): 1193-1202.
ERR550733	rpoB_S441L	katG_S315T	Uzbekistan	Walker, T.M., et al. The Lancet infectious diseases 15.10 (2015): 1193-1202.
ERR550735	rpoB_S450L	katG_S315T	Uzbekistan	Walker, T.M., et al. The Lancet infectious diseases 15.10 (2015): 1193-1202.
ERR550743	rpoB_S450L	katG_S315T	Uzbekistan	Walker, T.M., et al. The Lancet infectious diseases 15.10 (2015): 1193-1202.
ERR550748	rpoB_S450L	katG_S315T	Uzbekistan	Walker, T.M., et al. The Lancet infectious diseases 15.10 (2015): 1193-1202.
ERR550763	rpoB_S450L	katG_S315T	Uzbekistan	Walker, T.M., et al. The Lancet infectious diseases 15.10 (2015): 1193-1202.
ERR550770	rpoB_S450L	katG_S315T	Uzbekistan	Walker, T.M., et al. The Lancet infectious diseases 15.10 (2015): 1193-1202.
ERR550773	rpoB_S450L	katG_S315T	Uzbekistan	Walker, T.M., et al. The Lancet infectious diseases 15.10 (2015): 1193-1202.
ERR550778	rpoB_S450L	katG_S315T	Uzbekistan	Walker, T.M., et al. The Lancet infectious diseases 15.10 (2015): 1193-1202.
ERR550784	rpoB_S450L	katG_S315T	Uzbekistan	Walker, T.M., et al. The Lancet infectious diseases 15.10 (2015): 1193-1202.
ERR550786	rpoB_H445Y	katG_S315T	Uzbekistan	Walker, T.M., et al. The Lancet infectious diseases 15.10 (2015): 1193-1202.
ERR550806	rpoB_S450L	katG_W191R,fabG1_promoter_C1673425T	Uzbekistan	Walker, T.M., et al. The Lancet infectious diseases 15.10 (2015): 1193-1202.
ERR550809	rpoB_S450L	katG_S315T	Uzbekistan	Walker, T.M., et al. The Lancet infectious diseases 15.10 (2015): 1193-1202.
ERR550830	rpoB_S450L	katG_S315T	Uzbekistan	Walker, T.M., et al. The Lancet infectious diseases 15.10 (2015): 1193-1202.
ERR550838	rpoB_S450L	katG_S315T	Uzbekistan	Walker, T.M., et al. The Lancet infectious diseases 15.10 (2015): 1193-1202.
ERR550841	rpoB_S450L	katG_S315T	Uzbekistan	Walker, T.M., et al. The Lancet infectious diseases 15.10 (2015): 1193-1202.
ERR550887	rpoB_S450L	katG_S315T	Uzbekistan	Walker, T.M., et al. The Lancet infectious diseases 15.10 (2015): 1193-1202.

ERR550895	rpoB_S450L	katG_S315T	Uzbekistan	Walker, T.M., et al. The Lancet infectious diseases 15.10 (2015): 1193-1202.
ERR550897	rpoB_H445Y	katG_S315T	Uzbekistan	Walker, T.M., et al. The Lancet infectious diseases 15.10 (2015): 1193-1202.
ERR550908	rpoB_S450L	katG_S315T	Uzbekistan	Walker, T.M., et al. The Lancet infectious diseases 15.10 (2015): 1193-1202.
ERR550914	rpoB_S450L	katG_S315T	Uzbekistan	Walker, T.M., et al. The Lancet infectious diseases 15.10 (2015): 1193-1202.
ERR550923	rpoB_S450L	katG_S315T	Uzbekistan	Walker, T.M., et al. The Lancet infectious diseases 15.10 (2015): 1193-1202.
ERR550928	rpoB_S450L	katG_S315T	Uzbekistan	Walker, T.M., et al. The Lancet infectious diseases 15.10 (2015): 1193-1202.
ERR550950	rpoB_S450L	katG_S315T	Uzbekistan	Walker, T.M., et al. The Lancet infectious diseases 15.10 (2015): 1193-1202.
ERR550960	rpoB_S450L	katG_S315T	Uzbekistan	Walker, T.M., et al. The Lancet infectious diseases 15.10 (2015): 1193-1202.
ERR550980	rpoB_S450L	katG_S315T	Uzbekistan	Walker, T.M., et al. The Lancet infectious diseases 15.10 (2015): 1193-1202.
ERR550989	rpoB_S450L	katG_S315T	Uzbekistan	Walker, T.M., et al. The Lancet infectious diseases 15.10 (2015): 1193-1202.
ERR551000	rpoB_S450L	katG_S315T	Uzbekistan	Walker, T.M., et al. The Lancet infectious diseases 15.10 (2015): 1193-1202.
ERR551016	rpoB_S450L	katG_S315T	Uzbekistan	Walker, T.M., et al. The Lancet infectious diseases 15.10 (2015): 1193-1202.
ERR551020	rpoB_D435V	katG_S315T	Uzbekistan	Walker, T.M., et al. The Lancet infectious diseases 15.10 (2015): 1193-1202.
ERR551052	rpoB_S450L	katG_S315T	Uzbekistan	Walker, T.M., et al. The Lancet infectious diseases 15.10 (2015): 1193-1202.
ERR551063	rpoB_T400A,rpoB_S450L	katG_S315T	Uzbekistan	Walker, T.M., et al. The Lancet infectious diseases 15.10 (2015): 1193-1202.
ERR551099	rpoB_S450L	katG_S315T	Uzbekistan	Walker, T.M., et al. The Lancet infectious diseases 15.10 (2015): 1193-1202.
ERR551108	rpoB_S450L	katG_S315T	Uzbekistan	Walker, T.M., et al. The Lancet infectious diseases 15.10 (2015): 1193-1202.
ERR551128	rpoB_S450L	katG_S315T	Uzbekistan	Walker, T.M., et al. The Lancet infectious diseases 15.10 (2015): 1193-1202.
ERR551130	rpoB_S450W	ahpC_promoter_C2726139T	Uzbekistan	Walker, T.M., et al. The Lancet infectious diseases 15.10 (2015): 1193-1202.
ERR551132	rpoB_S450L	katG_S315T	Uzbekistan	Walker, T.M., et al. The Lancet infectious diseases 15.10 (2015): 1193-1202.
ERR551138	rpoB_S450L	katG_S315T	Uzbekistan	Walker, T.M., et al. The Lancet infectious diseases 15.10 (2015): 1193-1202.
ERR551142	rpoB_S450L	katG_S315T	Uzbekistan	Walker, T.M., et al. The Lancet infectious diseases 15.10 (2015): 1193-1202.
ERR551211	rpoB_S450L	katG_S315T	Uzbekistan	Walker, T.M., et al. The Lancet infectious diseases 15.10 (2015): 1193-1202.
ERR551220	rpoB_S450L	katG_S315T	Uzbekistan	Walker, T.M., et al. The Lancet infectious diseases 15.10 (2015): 1193-1202.
ERR551251	rpoB_S450L	katG_S315T	Uzbekistan	Walker, T.M., et al. The Lancet infectious diseases 15.10 (2015): 1193-1202.
ERR551259	rpoB_S450L	katG_S315T	Uzbekistan	Walker, T.M., et al. The Lancet infectious diseases 15.10 (2015): 1193-1202.
ERR551285	rpoB_S450L	katG_S315T	Uzbekistan	Walker, T.M., et al. The Lancet infectious diseases 15.10 (2015): 1193-1202.
ERR551295	rpoB_H445D	katG_S315T	Uzbekistan	Walker, T.M., et al. The Lancet infectious diseases 15.10 (2015): 1193-1202.
ERR551305	rpoB_S450L	katG_S315T	Uzbekistan	Walker, T.M., et al. The Lancet infectious diseases 15.10 (2015): 1193-1202.
ERR551321	rpoB_S450L	katG_S315T	Uzbekistan	Walker, T.M., et al. The Lancet infectious diseases 15.10 (2015): 1193-1202.
ERR551352	rpoB_S450L	katG_S315T	Uzbekistan	Walker, T.M., et al. The Lancet infectious diseases 15.10 (2015): 1193-1202.
ERR551360	rpoB_S450L	katG_S315T	Uzbekistan	Walker, T.M., et al. The Lancet infectious diseases 15.10 (2015): 1193-1202.
ERR551372	rpoB_S450L	katG_S315T	Uzbekistan	Walker, T.M., et al. The Lancet infectious diseases 15.10 (2015): 1193-1202.
ERR551377	rpoB_S450L	katG_S315T	Uzbekistan	Walker, T.M., et al. The Lancet infectious diseases 15.10 (2015): 1193-1202.
ERR551380	rpoB_D435V	ahpC_promoter_C2726141T	Uzbekistan	Walker, T.M., et al. The Lancet infectious diseases 15.10 (2015): 1193-1202.
ERR551409	rpoB_S450L	katG_S315T	Uzbekistan	Walker, T.M., et al. The Lancet infectious diseases 15.10 (2015): 1193-1202.
ERR551417	rpoB_S450L	katG_S315T	Uzbekistan	Walker, T.M., et al. The Lancet infectious diseases 15.10 (2015): 1193-1202.
ERR551429	rpoB_S450L	katG_S315T	Uzbekistan	Walker, T.M., et al. The Lancet infectious diseases 15.10 (2015): 1193-1202.
ERR551431	rpoB_S450L	katG_S315T	Uzbekistan	Walker, T.M., et al. The Lancet infectious diseases 15.10 (2015): 1193-1202.
ERR551437	rpoB_S450L	katG_S315T	Uzbekistan	Walker, T.M., et al. The Lancet infectious diseases 15.10 (2015): 1193-1202.
ERR551460	rpoB_S450L	katG_S315T	Uzbekistan	Walker, T.M., et al. The Lancet infectious diseases 15.10 (2015): 1193-1202.
ERR551464	rpoB_S450L	katG_S315T	Uzbekistan	Walker, T.M., et al. The Lancet infectious diseases 15.10 (2015): 1193-1202.
ERR551466	rpoB_S450L	ahpC_promoter_G2726145A	Uzbekistan	Walker, T.M., et al. The Lancet infectious diseases 15.10 (2015): 1193-1202.
ERR551468	rpoB_H445D	inhA_I194T,fabG1_promoter_C1673425T	Uzbekistan	Walker, T.M., et al. The Lancet infectious diseases 15.10 (2015): 1193-1202.
ERR551478	rpoB_L452P	katG_S315T	Uzbekistan	Walker, T.M., et al. The Lancet infectious diseases 15.10 (2015): 1193-1202.
ERR551495	rpoB_S450L	katG_S315T	Uzbekistan	Walker, T.M., et al. The Lancet infectious diseases 15.10 (2015): 1193-1202.
ERR551539	rpoB_S450L	katG_S315T	Uzbekistan	Walker, T.M., et al. The Lancet infectious diseases 15.10 (2015): 1193-1202.
ERR551558	rpoB_S450L	katG_S315T	Uzbekistan	Walker, T.M., et al. The Lancet infectious diseases 15.10 (2015): 1193-1202.
ERR551582	rpoB_S450L	katG_S315T	Uzbekistan	Walker, T.M., et al. The Lancet infectious diseases 15.10 (2015): 1193-1202.
ERR551592	rpoB_H445D	katG_S315T	Uzbekistan	Walker, T.M., et al. The Lancet infectious diseases 15.10 (2015): 1193-1202.
ERR551596	rpoB_S450L	katG_S315T	Uzbekistan	Walker, T.M., et al. The Lancet infectious diseases 15.10 (2015): 1193-1202.
ERR551600	rpoB_S450L	katG_S315T	Uzbekistan	Walker, T.M., et al. The Lancet infectious diseases 15.10 (2015): 1193-1202.
ERR551603	rpoB_S450L	katG_S315T	Uzbekistan	Walker, T.M., et al. The Lancet infectious diseases 15.10 (2015): 1193-1202.
ERR551611	rpoB_S450L	katG_S315T	Uzbekistan	Walker, T.M., et al. The Lancet infectious diseases 15.10 (2015): 1193-1202.
ERR551624	rpoB_S450L	katG_S315T	Uzbekistan	Walker, T.M., et al. The Lancet infectious diseases 15.10 (2015): 1193-1202.
ERR551638	rpoB_S450L	katG_S315T	Uzbekistan	Walker, T.M., et al. The Lancet infectious diseases 15.10 (2015): 1193-1202.
ERR551643	rpoB_L452P	katG_S315T	Uzbekistan	Walker, T.M., et al. The Lancet infectious diseases 15.10 (2015): 1193-1202.
ERR551645	rpoB_S450L	katG_S315T	Uzbekistan	Walker, T.M., et al. The Lancet infectious diseases 15.10 (2015): 1193-1202.
ERR551648	rpoB_S450L	katG_S315T	Uzbekistan	Walker, T.M., et al. The Lancet infectious diseases 15.10 (2015): 1193-1202.
ERR551660	rpoB_S450L	katG_S315T	Uzbekistan	Walker, T.M., et al. The Lancet infectious diseases 15.10 (2015): 1193-1202.
ERR551667	rpoB_S450L	katG_S315T	Uzbekistan	Walker, T.M., et al. The Lancet infectious diseases 15.10 (2015): 1193-1202.

ERR551697	rpoB_S450L	katG_S315T	Uzbekistan	Walker, T.M., et al. The Lancet infectious diseases 15.10 (2015): 1193-1202.
ERR551708	rpoB_A286V,rpoB_S450L	katG_S315T	Uzbekistan	Walker, T.M., et al. The Lancet infectious diseases 15.10 (2015): 1193-1202.
ERR551727	rpoB_S450L	katG_S315T	Uzbekistan	Walker, T.M., et al. The Lancet infectious diseases 15.10 (2015): 1193-1202.
ERR551735	rpoB_S450L	katG_S315T	Uzbekistan	Walker, T.M., et al. The Lancet infectious diseases 15.10 (2015): 1193-1202.
ERR551738	rpoB_S450L	katG_S315T	Uzbekistan	Walker, T.M., et al. The Lancet infectious diseases 15.10 (2015): 1193-1202.
ERR551745	rpoB_S450L	katG_S315T	Uzbekistan	Walker, T.M., et al. The Lancet infectious diseases 15.10 (2015): 1193-1202.
ERR551749	rpoB_S450L	katG_S315T	Uzbekistan	Walker, T.M., et al. The Lancet infectious diseases 15.10 (2015): 1193-1202.
ERR551767	rpoB_S450L	katG_S315T	Uzbekistan	Walker, T.M., et al. The Lancet infectious diseases 15.10 (2015): 1193-1202.
ERR551771	rpoB_S450L	katG_S315T	Uzbekistan	Walker, T.M., et al. The Lancet infectious diseases 15.10 (2015): 1193-1202.
ERR551776	rpoB_S450L	katG_S315T	Uzbekistan	Walker, T.M., et al. The Lancet infectious diseases 15.10 (2015): 1193-1202.
ERR551780	rpoB_S450L	katG_S315T,fabG1_promoter_T1673432C	Uzbekistan	Walker, T.M., et al. The Lancet infectious diseases 15.10 (2015): 1193-1202.
ERR551793	rpoB_S450L	katG_S315T	Uzbekistan	Walker, T.M., et al. The Lancet infectious diseases 15.10 (2015): 1193-1202.
ERR551798	rpoB_S450L	katG_S315T	Uzbekistan	Walker, T.M., et al. The Lancet infectious diseases 15.10 (2015): 1193-1202.
ERR551806	rpoB_S450L	katG_S315T	Uzbekistan	Walker, T.M., et al. The Lancet infectious diseases 15.10 (2015): 1193-1202.
ERR551811	rpoB_S450L	katG_S315T	Uzbekistan	Walker, T.M., et al. The Lancet infectious diseases 15.10 (2015): 1193-1202.
ERR551816	rpoB_S450L	katG_S315T	Uzbekistan	Walker, T.M., et al. The Lancet infectious diseases 15.10 (2015): 1193-1202.
ERR551822	rpoB_S450L	katG_S315T	Uzbekistan	Walker, T.M., et al. The Lancet infectious diseases 15.10 (2015): 1193-1202.
ERR551827	rpoB_H445Y	katG_S315T	Uzbekistan	Walker, T.M., et al. The Lancet infectious diseases 15.10 (2015): 1193-1202.
ERR551828	rpoB_H445R	ahpC_promoter_G2726145A	Uzbekistan	Walker, T.M., et al. The Lancet infectious diseases 15.10 (2015): 1193-1202.
ERR551844	rpoB_S450L	katG_S315T	Uzbekistan	Walker, T.M., et al. The Lancet infectious diseases 15.10 (2015): 1193-1202.
ERR551862	rpoB_S450L	katG_S315T	Uzbekistan	Walker, T.M., et al. The Lancet infectious diseases 15.10 (2015): 1193-1202.
ERR551877	rpoB_S450L	katG_S315T	Uzbekistan	Walker, T.M., et al. The Lancet infectious diseases 15.10 (2015): 1193-1202.
ERR551894	rpoB_S450L	katG_S315T	Uzbekistan	Walker, T.M., et al. The Lancet infectious diseases 15.10 (2015): 1193-1202.
ERR551910	rpoB_V170F	katG_S315T	Uzbekistan	Walker, T.M., et al. The Lancet infectious diseases 15.10 (2015): 1193-1202.
ERR551918	rpoB_S450L	katG_Q461P,fabG1_promoter_C1673425T	Uzbekistan	Walker, T.M., et al. The Lancet infectious diseases 15.10 (2015): 1193-1202.
ERR551923	rpoB_S450L	katG_S315T	Uzbekistan	Walker, T.M., et al. The Lancet infectious diseases 15.10 (2015): 1193-1202.
ERR551925	rpoB_S450L	kasA_G269S,katG_S315T	Uzbekistan	Walker, T.M., et al. The Lancet infectious diseases 15.10 (2015): 1193-1202.
ERR551945	rpoB_S450L	katG_S315T	Uzbekistan	Walker, T.M., et al. The Lancet infectious diseases 15.10 (2015): 1193-1202.
ERR551947	rpoB_Q432P	katG_S315T	Uzbekistan	Walker, T.M., et al. The Lancet infectious diseases 15.10 (2015): 1193-1202.
ERR551948	rpoB_S450L	katG_R104Q	Uzbekistan	Walker, T.M., et al. The Lancet infectious diseases 15.10 (2015): 1193-1202.
ERR551952	rpoB_S450L	katG_S315T	Uzbekistan	Walker, T.M., et al. The Lancet infectious diseases 15.10 (2015): 1193-1202.
ERR551974	rpoB_S450L	katG_S315T	Uzbekistan	Walker, T.M., et al. The Lancet infectious diseases 15.10 (2015): 1193-1202.
ERR551985	rpoB_S450L	kasA_G269S,katG_S315T,fabG1_promoter_C1673425T	Uzbekistan	Walker, T.M., et al. The Lancet infectious diseases 15.10 (2015): 1193-1202.
ERR551986	rpoB_T400A,rpoB_S450L	katG_S315T	Uzbekistan	Walker, T.M., et al. The Lancet infectious diseases 15.10 (2015): 1193-1202.
ERR551996	rpoB_S450L	katG_S315T	Uzbekistan	Walker, T.M., et al. The Lancet infectious diseases 15.10 (2015): 1193-1202.
ERR552007	rpoB_S450L	katG_S315T	Uzbekistan	Walker, T.M., et al. The Lancet infectious diseases 15.10 (2015): 1193-1202.
ERR552012	rpoB_S450L	katG_S315T	Uzbekistan	Walker, T.M., et al. The Lancet infectious diseases 15.10 (2015): 1193-1202.
ERR552033	rpoB_S450L	kasA_G269S,katG_S315T	Uzbekistan	Walker, T.M., et al. The Lancet infectious diseases 15.10 (2015): 1193-1202.
ERR552042	rpoB_S450L	katG_S315T	Uzbekistan	Walker, T.M., et al. The Lancet infectious diseases 15.10 (2015): 1193-1202.
ERR552065	rpoB_S450L	katG_S315T	Uzbekistan	Walker, T.M., et al. The Lancet infectious diseases 15.10 (2015): 1193-1202.
ERR552068	rpoB_S450L	katG_S315T	Uzbekistan	Walker, T.M., et al. The Lancet infectious diseases 15.10 (2015): 1193-1202.
ERR552080	rpoB_S450L	kasA_G269S,katG_S315T	Uzbekistan	Walker, T.M., et al. The Lancet infectious diseases 15.10 (2015): 1193-1202.
ERR552099	rpoB_S450L	katG_S315T	Uzbekistan	Walker, T.M., et al. The Lancet infectious diseases 15.10 (2015): 1193-1202.
ERR552102	rpoB_S450L	katG_S315T	Uzbekistan	Walker, T.M., et al. The Lancet infectious diseases 15.10 (2015): 1193-1202.
ERR552104	rpoB_S450L	katG_S315T	Uzbekistan	Walker, T.M., et al. The Lancet infectious diseases 15.10 (2015): 1193-1202.
ERR552106	rpoB_S450L	katG_S315T	Uzbekistan	Walker, T.M., et al. The Lancet infectious diseases 15.10 (2015): 1193-1202.
ERR552112	rpoB_S450L	kasA_G269S,katG_S315T	Uzbekistan	Walker, T.M., et al. The Lancet infectious diseases 15.10 (2015): 1193-1202.
ERR552123	rpoB_S450L	katG_L141F,kasA_G269S	Uzbekistan	Walker, T.M., et al. The Lancet infectious diseases 15.10 (2015): 1193-1202.
ERR552142	rpoB_S450L	katG_S315T,fabG1_promoter_T1673432C	Uzbekistan	Walker, T.M., et al. The Lancet infectious diseases 15.10 (2015): 1193-1202.
ERR552148	rpoB_S450L	katG_S315T	Uzbekistan	Walker, T.M., et al. The Lancet infectious diseases 15.10 (2015): 1193-1202.
ERR552164	rpoB_S450L	katG_S315T	Uzbekistan	Walker, T.M., et al. The Lancet infectious diseases 15.10 (2015): 1193-1202.
ERR552200	rpoB_S450L	katG_S315T	Uzbekistan	Walker, T.M., et al. The Lancet infectious diseases 15.10 (2015): 1193-1202.
ERR552215	rpoB_S450L	katG_S315T,fabG1_promoter_T1673432C	Uzbekistan	Walker, T.M., et al. The Lancet infectious diseases 15.10 (2015): 1193-1202.
ERR552226	rpoB_S450L	katG_S315T	Uzbekistan	Walker, T.M., et al. The Lancet infectious diseases 15.10 (2015): 1193-1202.
ERR552238	rpoB_S450L	katG_S315T	Uzbekistan	Walker, T.M., et al. The Lancet infectious diseases 15.10 (2015): 1193-1202.

ERR552244	rpoB_S450L	katG_S315T	Uzbekistan	Walker, T.M., et al. The Lancet infectious diseases 15.10 (2015): 1193-1202.
ERR552258	rpoB_S450L	katG_S315T	Uzbekistan	Walker, T.M., et al. The Lancet infectious diseases 15.10 (2015): 1193-1202.
ERR552286	rpoB_S450L	katG_S315T	Uzbekistan	Walker, T.M., et al. The Lancet infectious diseases 15.10 (2015): 1193-1202.
ERR552298	rpoB_S450L	kasA_G269S,katG_S315T	Uzbekistan	Walker, T.M., et al. The Lancet infectious diseases 15.10 (2015): 1193-1202.
ERR552301	rpoB_D435F	katG_S315T	Uzbekistan	Walker, T.M., et al. The Lancet infectious diseases 15.10 (2015): 1193-1202.
ERR552322	rpoB_S450L	katG_S315T	Uzbekistan	Walker, T.M., et al. The Lancet infectious diseases 15.10 (2015): 1193-1202.
ERR552347	rpoB_S450L	katG_S315T	Uzbekistan	Walker, T.M., et al. The Lancet infectious diseases 15.10 (2015): 1193-1202.
ERR552363	rpoB_S450L	katG_S315T	Uzbekistan	Walker, T.M., et al. The Lancet infectious diseases 15.10 (2015): 1193-1202.
ERR552373	rpoB_S450L	katG_S315T	Uzbekistan	Walker, T.M., et al. The Lancet infectious diseases 15.10 (2015): 1193-1202.
ERR552377	rpoB_S450L	katG_S315T	Uzbekistan	Walker, T.M., et al. The Lancet infectious diseases 15.10 (2015): 1193-1202.
ERR552384	rpoB_S450L	katG_S315T	Uzbekistan	Walker, T.M., et al. The Lancet infectious diseases 15.10 (2015): 1193-1202.
ERR552390	rpoB_S450L	katG_S315T	Uzbekistan	Walker, T.M., et al. The Lancet infectious diseases 15.10 (2015): 1193-1202.
ERR552398	rpoB_S450L	katG_S315T	Uzbekistan	Walker, T.M., et al. The Lancet infectious diseases 15.10 (2015): 1193-1202.
ERR552401	rpoB_S450L	katG_S315T	Uzbekistan	Walker, T.M., et al. The Lancet infectious diseases 15.10 (2015): 1193-1202.
ERR552413	rpoB_S450L	katG_S315T,fabG1_promoter_C1673425T	Uzbekistan	Walker, T.M., et al. The Lancet infectious diseases 15.10 (2015): 1193-1202.
ERR552418	rpoB_S450L	katG_S315T	Uzbekistan	Walker, T.M., et al. The Lancet infectious diseases 15.10 (2015): 1193-1202.
ERR552433	rpoB_H445Y	katG_S315T	Uzbekistan	Walker, T.M., et al. The Lancet infectious diseases 15.10 (2015): 1193-1202.
ERR552443	rpoB_S450W	ahpC_promoter_C2726139T	Uzbekistan	Walker, T.M., et al. The Lancet infectious diseases 15.10 (2015): 1193-1202.
ERR552447	rpoB_S450L	katG_S315T	Uzbekistan	Walker, T.M., et al. The Lancet infectious diseases 15.10 (2015): 1193-1202.
ERR552458	rpoB_L452P	katG_S315T	Uzbekistan	Walker, T.M., et al. The Lancet infectious diseases 15.10 (2015): 1193-1202.
ERR552499	rpoB_S450L	kasA_G269S,katG_S315T,fabG1_promoter_C1673425T	Uzbekistan	Walker, T.M., et al. The Lancet infectious diseases 15.10 (2015): 1193-1202.
ERR552501	rpoB_S450L	katG_S315T	Uzbekistan	Walker, T.M., et al. The Lancet infectious diseases 15.10 (2015): 1193-1202.
ERR552504	rpoB_L452P	katG_S315T	Uzbekistan	Walker, T.M., et al. The Lancet infectious diseases 15.10 (2015): 1193-1202.
ERR552510	rpoB_S450L	katG_S315T	Uzbekistan	Walker, T.M., et al. The Lancet infectious diseases 15.10 (2015): 1193-1202.
ERR552513	rpoB_S450L	katG_S315T	Uzbekistan	Walker, T.M., et al. The Lancet infectious diseases 15.10 (2015): 1193-1202.
ERR552519	rpoB_S450L	katG_S315T	Uzbekistan	Walker, T.M., et al. The Lancet infectious diseases 15.10 (2015): 1193-1202.
ERR552531	rpoB_S450L	katG_S315T	Uzbekistan	Walker, T.M., et al. The Lancet infectious diseases 15.10 (2015): 1193-1202.
ERR552545	rpoB_S450L	katG_S315T	Uzbekistan	Walker, T.M., et al. The Lancet infectious diseases 15.10 (2015): 1193-1202.
ERR552559	rpoB_D435V	katG_S315T	Uzbekistan	Walker, T.M., et al. The Lancet infectious diseases 15.10 (2015): 1193-1202.
ERR552570	rpoB_S450L	katG_S315T	Uzbekistan	Walker, T.M., et al. The Lancet infectious diseases 15.10 (2015): 1193-1202.
ERR552586	rpoB_S450L	katG_S315T	Uzbekistan	Walker, T.M., et al. The Lancet infectious diseases 15.10 (2015): 1193-1202.
ERR552603	rpoB_S450L	katG_S315T	Uzbekistan	Walker, T.M., et al. The Lancet infectious diseases 15.10 (2015): 1193-1202.
ERR552607	rpoB_S450L	katG_S315T	Uzbekistan	Walker, T.M., et al. The Lancet infectious diseases 15.10 (2015): 1193-1202.
ERR552611	rpoB_S450L	inhA_I21T,kasA_G269S,fabG1_promoter_C1673425T	Uzbekistan	Walker, T.M., et al. The Lancet infectious diseases 15.10 (2015): 1193-1202.
ERR552626	rpoB_H445D	katG_S315T	Uzbekistan	Walker, T.M., et al. The Lancet infectious diseases 15.10 (2015): 1193-1202.
ERR552631	rpoB_S450L	katG_S315T	Uzbekistan	Walker, T.M., et al. The Lancet infectious diseases 15.10 (2015): 1193-1202.
ERR552653	rpoB_M434I,rpoB_D435Y	katG_S315T	Uzbekistan	Walker, T.M., et al. The Lancet infectious diseases 15.10 (2015): 1193-1202.
ERR552661	rpoB_S450L	katG_S315T	Uzbekistan	Walker, T.M., et al. The Lancet infectious diseases 15.10 (2015): 1193-1202.
ERR552708	rpoB_S450L	katG_S315T	Uzbekistan	Walker, T.M., et al. The Lancet infectious diseases 15.10 (2015): 1193-1202.
ERR552711	rpoB_S450L	katG_S315T	Uzbekistan	Walker, T.M., et al. The Lancet infectious diseases 15.10 (2015): 1193-1202.
ERR552719	rpoB_S450L	fabG1_promoter_C1673425T	Uzbekistan	Walker, T.M., et al. The Lancet infectious diseases 15.10 (2015): 1193-1202.
ERR552734	rpoB_S450L	katG_S315T	Uzbekistan	Walker, T.M., et al. The Lancet infectious diseases 15.10 (2015): 1193-1202.
ERR552738	rpoB_D435V	katG_S315T,fabG1_promoter_G1673423T	Uzbekistan	Walker, T.M., et al. The Lancet infectious diseases 15.10 (2015): 1193-1202.
ERR552741	rpoB_S450L	katG_S315T	Uzbekistan	Walker, T.M., et al. The Lancet infectious diseases 15.10 (2015): 1193-1202.
ERR552771	rpoB_Q429H,rpoB_H445R	katG_S315T	Uzbekistan	Walker, T.M., et al. *The Lancet infectious diseases* 15.10 (2015): 1193-1202.
ERR552791	rpoB_S450L	katG_S315T	Uzbekistan	Walker, T.M., et al. The Lancet infectious diseases 15.10 (2015): 1193-1202.
ERR552793	rpoB_S450L	katG_S315T	Uzbekistan	Walker, T.M., et al. The Lancet infectious diseases 15.10 (2015): 1193-1202.
ERR552816	rpoB_S450L	katG_S315T	Uzbekistan	Walker, T.M., et al. The Lancet infectious diseases 15.10 (2015): 1193-1202.
ERR552819	rpoB_S450L	katG_S315T	Uzbekistan	Walker, T.M., et al. The Lancet infectious diseases 15.10 (2015): 1193-1202.
ERR552834	rpoB_S450L	katG_S315T	Uzbekistan	Walker, T.M., et al. The Lancet infectious diseases 15.10 (2015): 1193-1202.
ERR552842	rpoB_S450L	katG_S315T	Uzbekistan	Walker, T.M., et al. The Lancet infectious diseases 15.10 (2015): 1193-1202.
ERR552872	rpoB_Q429H,rpoB_H445Y	katG_S700P,ahpC_promoter_C2726112T	Uzbekistan	Walker, T.M., et al. The Lancet infectious diseases 15.10 (2015): 1193-1202.
ERR552882	rpoB_S450L	katG_S315T	Uzbekistan	Walker, T.M., et al. The Lancet infectious diseases 15.10 (2015): 1193-1202.
ERR552897	rpoB_S450L	katG_S315T	Uzbekistan	Walker, T.M., et al. The Lancet infectious diseases 15.10 (2015): 1193-1202.
ERR552906	rpoB_S450L	katG_S315T	Uzbekistan	Walker, T.M., et al. The Lancet infectious diseases 15.10 (2015): 1193-1202.
ERR552926	rpoB_S450L	katG_S315T	Uzbekistan	Walker, T.M., et al. The Lancet infectious diseases 15.10 (2015): 1193-1202.

ERR552931	rpoB_D435V	katG_S315T	Uzbekistan	Walker, T.M., et al. The Lancet infectious diseases 15.10 (2015): 1193-1202.
ERR552940	rpoB_S450L	katG_S315T	Uzbekistan	Walker, T.M., et al. The Lancet infectious diseases 15.10 (2015): 1193-1202.
ERR552950	rpoB_S450L	katG_S315T	Uzbekistan	Walker, T.M., et al. The Lancet infectious diseases 15.10 (2015): 1193-1202.
ERR552955	rpoB_S450L	katG_S315T	Uzbekistan	Walker, T.M., et al. The Lancet infectious diseases 15.10 (2015): 1193-1202.
ERR552978	rpoB_S450L	katG_S315T	Uzbekistan	Walker, T.M., et al. The Lancet infectious diseases 15.10 (2015): 1193-1202.
ERR552982	rpoB_S450L	katG_S315T	Uzbekistan	Walker, T.M., et al. The Lancet infectious diseases 15.10 (2015): 1193-1202.
ERR553023	rpoB_S450L	kasA_G269S,katG_S315T	Uzbekistan	Walker, T.M., et al. The Lancet infectious diseases 15.10 (2015): 1193-1202.
ERR553051	rpoB_S450L	katG_S315T	Uzbekistan	Walker, T.M., et al. The Lancet infectious diseases 15.10 (2015): 1193-1202.
ERR553098	rpoB_S450L	katG_S315N	Uzbekistan	Walker, T.M., et al. The Lancet infectious diseases 15.10 (2015): 1193-1202.
ERR553153	rpoB_S450L	katG_S315T	Uzbekistan	Walker, T.M., et al. The Lancet infectious diseases 15.10 (2015): 1193-1202.
ERR553163	rpoB_S450W	katG_S315N	Uzbekistan	Walker, T.M., et al. The Lancet infectious diseases 15.10 (2015): 1193-1202.
ERR553169	rpoB_S450L	katG_S315T	Uzbekistan	Walker, T.M., et al. The Lancet infectious diseases 15.10 (2015): 1193-1202.
ERR553179	rpoB_S450L	katG_S315T	Uzbekistan	Walker, T.M., et al. The Lancet infectious diseases 15.10 (2015): 1193-1202.
ERR553180	rpoB_S450L	katG_S315T	Uzbekistan	Walker, T.M., et al. The Lancet infectious diseases 15.10 (2015): 1193-1202.
ERR553184	rpoB_S450L	katG_S315T	Uzbekistan	Walker, T.M., et al. The Lancet infectious diseases 15.10 (2015): 1193-1202.
ERR553188	rpoB_S450L	katG_S315T	Uzbekistan	Walker, T.M., et al. The Lancet infectious diseases 15.10 (2015): 1193-1202.
ERR553192	rpoB_D435V	katG_S315T	Uzbekistan	Walker, T.M., et al. The Lancet infectious diseases 15.10 (2015): 1193-1202.
ERR553194	rpoB_S450L	katG_S315T,fabG1_promoter_T1673432C	Uzbekistan	Walker, T.M., et al. The Lancet infectious diseases 15.10 (2015): 1193-1202.
ERR553198	rpoB_S450L	katG_G297V	Uzbekistan	Walker, T.M., et al. The Lancet infectious diseases 15.10 (2015): 1193-1202.
ERR553217	rpoB_S450L	katG_S315T	Uzbekistan	Walker, T.M., et al. The Lancet infectious diseases 15.10 (2015): 1193-1202.
ERR553246	rpoB_S450L	katG_S315T	Uzbekistan	Walker, T.M., et al. The Lancet infectious diseases 15.10 (2015): 1193-1202.
ERR553394	rpoB_S450L	katG_S315T	Uzbekistan	Walker, T.M., et al. The Lancet infectious diseases 15.10 (2015): 1193-1202.

Supplementary table 4. Putative compensatory mutations in *rpoA, rpoB and rpoC* genes identified in this study and in comparison with previous studies

Gene	Polymorphism	Hit number	Idependent events (NJ)	Idependent events (ML)	Comas et al	de Vos et al	Casali et al	Song et al	Cohen et al
rpoA	G31S	4	3	3			yes^		yes
rpoA	R182L	5	2	2	yes				
rpoA	T181A	2	2	2	yes		yes		
rpoA	T187A	23	8	8	yes*		yes^		yes
rpoA	T187P	3	3	3	yes*		yes^		
rpoA	V183G	12	5	5	yes		yes		yes
rpoB	A451V	3	2	2					
rpoB	E481A	3	2	2					
rpoB	E761D	216	4	6			yes		
rpoB	F503S	5	2	2	yes			yes	
rpoB	H835R	5	3	3			yes^		
rpoB	I488V	17	2	2			yes		
rpoB	I491V	3	3	3			yes^		
rpoB	L731P	6	3	3			yes		
rpoB	M434V	3	2	2					
rpoB	P45L	4	2	2					yes
rpoB	P45R	2	2	2					
rpoB	P45S	5	5	5			yes		
rpoB	R219C	2	2	2					
rpoB	R827C	9	3	3	yes				yes
rpoB	S874Y	2	2	2					
rpoB	T676P	2	2	2					
rpoC	A521D	2	2	2	yes		yes^		
rpoC	D485N	24	6	6	yes*		yes^		
rpoC	D485Y	5	4	4	yes		yes^		
rpoC	D747A	6	2	2			yes		
rpoC	E1033K	2	2	2					
rpoC	E750Q	5	2	2					
rpoC	F452C	2	2	2	yes		yes		
rpoC	G332C	2	2	2			yes^		
rpoC	G332R	9	6	6	yes		yes^		
rpoC	G332S	2	2	2	yes		yes^		yes
rpoC	G433S	6	5	5	yes		yes^		
rpoC	G519D	4	4	4	yes		yes^		
rpoC	G519R	2	2	2					
rpoC	H525Q	7	4	4	yes	yes			yes
rpoC	I491V	22	14	13	yes*	yes#			yes
rpoC	I885V	6	3	3	yes				yes
rpoC	K445R	21	6	6			yes^		
rpoC	L449V	12	5	5	yes		yes^		
rpoC	L516P	7	5	5	yes		yes		
rpoC	L527V	19	4	4	yes		yes^		
rpoC	N416S	3	3	3	yes			yes	
rpoC	N698H	5	3	3	yes*	yes			
rpoC	N698K	5	4	4	yes*				yes
rpoC	N698S	80	7	7	yes*	yes			
rpoC	N826K	3	2	2	yes				yes
rpoC	P1040R	4	4	4	yes	yes			yes
rpoC	P1040S	28	8	8	yes				yes
rpoC	P434Q	3	3	3			yes^		
rpoC	P434R	4	2	2	yes*		yes^		
rpoC	Q1125H	2	2	2	yes				yes
rpoC	R459W	2	2	2					yes
rpoC	T825A	2	2	2					
rpoC	V1252L	15	5	5	yes	yes#			yes
rpoC	V431M	9	5	5			yes^		
rpoC	V483A	22	13	13	yes*	yes	yes^		

rpoC	V483G	55	26	26	yes*	yes#	yes^	yes	yes
rpoC	V517L	3	2	2	yes		yes^		
rpoC	W484G	13	9	9	yes		yes^		

*: these mutations have effect on protein function using SIFT scores and competitive experiment

#: these mutations evovled independently in phylogenetically distant strain families as defined by spoligotyping

^: these mutations emerged independently multiple times in isolates with RRDR mutations

Supplementary Table 5. Comparison of ratios of compenstaory mutations in clustered and non-clustered MDR-TB strains withiout excluding M type clusters.

Groups	Total	With CMs	Without CMs	χ2	P value
Clustered MDR-TB	94	42 (44.7%)	52 (55.3%)	4.557	0.033
Non-clustered MDR-TB	133	41 (30.8%)	92 (69.2%)		

CMs: Compensatory mutations
